# Fluorohydrin Synthesis via Formal C–H Fluorination of Cyclic Alcohols

**DOI:** 10.1021/acs.joc.5c02401

**Published:** 2026-01-13

**Authors:** Helen M.J. Edens, Julian G. West

**Affiliations:** Department of Chemistry, Rice University, Houston, Texas 77030, United States

## Abstract

Installation of C–F bonds, particularly at the expense of C–H bonds, is of exceptional importance in the development of pharmaceuticals, agrochemicals, and advanced materials. Toward introducing this moiety, here we show that cyclic fluorohydrins can be directly generated via formal C–H fluorination of 5-, 6-, 7-, and 8-membered cyclic alcohols using Selectfluor. Mechanistic studies are consistent with fluorohydrin formation via an ionic elimination-hydroxyfluorination cascade proceeding through an alkene intermediate.

## Graphical abstract

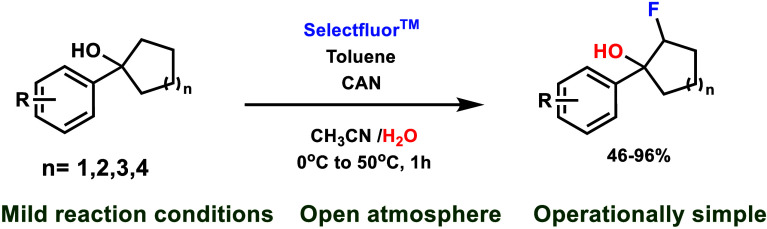

## Introduction

Fluorine chemistry has become increasingly relevant in the modern age due to the valuable characteristics that C–F bonds impart to materials, agrochemicals, and pharmaceuticals.^1,2^ The unique properties of the fluorine atom, compared to hydrogen, can create large differences in molecular properties, such as lipophilicity, polarizability, and biological longevity, with a relatively small change in connectivity and steric demand. As a result, significant efforts continue to be devoted to installing this versatile atom site selectively in organic molecules.

Early work in our group and others^3–8^ sought to achieve C–F bond formation via oxidative cleavage of cyclic alcohols to yield distal fluoroketones (Figure 1). These methods take advantage of the high level of ring strain in cyclobutyl (26.4 kcal/mol)^9^ and cyclopropyl (27.6 kcal/mol)^9^ ring systems to enable C–C bond cleavage under mild reaction conditions, using bench-stable reagents and a range of stoichiometric and catalytic metals, including earth-abundant cerium^3^ and manganese^4^ (Figure 1). Based on the success of this approach across a range of conditions, we wondered if less-strained 5- (6.5 kcal/mol),^9^ 6- (0 kcal/mol),^9^ 7- (6.3 kcal/mol),^9^ and 8- (9.6 kcal/mol)^9^ membered ring system cyclic alcohols might also be amenable to this strategy and allow generation of extended distal fluoroketones.

**Figure 1:**
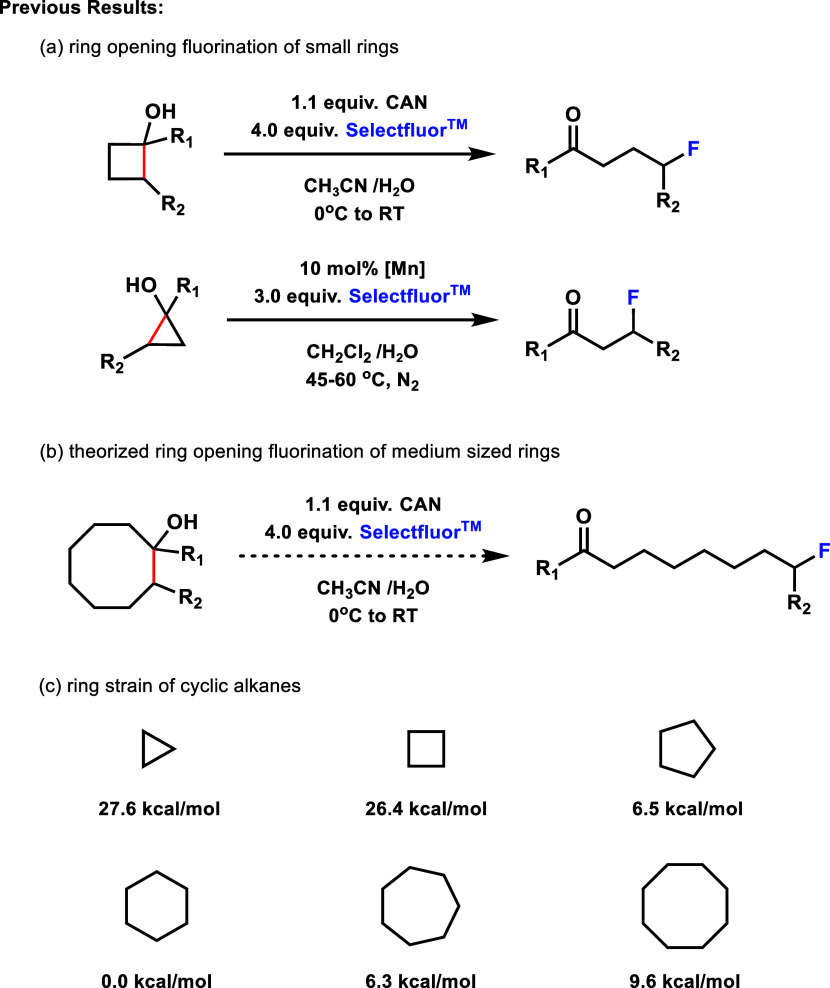
Successful ring-opening fluorination of strained cyclobutyl and cyclopropyl alcohols led us to hypothesize that larger ring cyclic alcohols might also undergo this reaction; however, the ring strain of these alcohols is significantly reduced.

Initial exploration in this vein revealed that 5-, 6-, 7-, and 8-member cyclic alcohols do not undergo the expected C–C fluorination process and instead produce fluorohydrin products via a formal C–H fluorination process. As these motifs are synthetically valuable due to their use as precursors in the production of biologically active analogs of natural products,^10^ we continued exploration into this sudden change in reactivity and sought to better understand the basis of this divergent reactivity compared to cyclobutanols and cyclopropanols. We were further driven to explore this finding to understand the complementary properties and potential advantages it has compared to current fluorohydrin syntheses.

Current synthetic routes for fluorohydrins proceed predominantly through α-fluoro carbonyl reduction, epoxide ring-opening strategies, and carbon–carbon bond formation to append prefluorinated building blocks.^10^ Alternate methodologies stem from olefins, particularly styrene precursors, and rely upon strong electron-donating groups on one or both sides of the double bond to stabilize a key carbocation intermediate formed after electrophilic fluorination. This carbocation can then be captured by water cosolvent to give synthetically relevant yields of the fluorohydrin (Figure 2).^11–18^ Early work from Desmarteaux and Stavber demonstrated this approach utilizing Selectfluor and NFTh (Accufluor) to convert styrene and styrene analogs under elevated temperatures.^19–21^ Intriguingly, Stavber found that some tertiary alcohols could be converted to fluorohydrins using Selectfluor;^19^ however, this method requires forcing temperatures and focused on methyl fluorination. Further, no mechanistic investigation was conducted, preventing rational elaboration of this approach.

**Figure 2:**
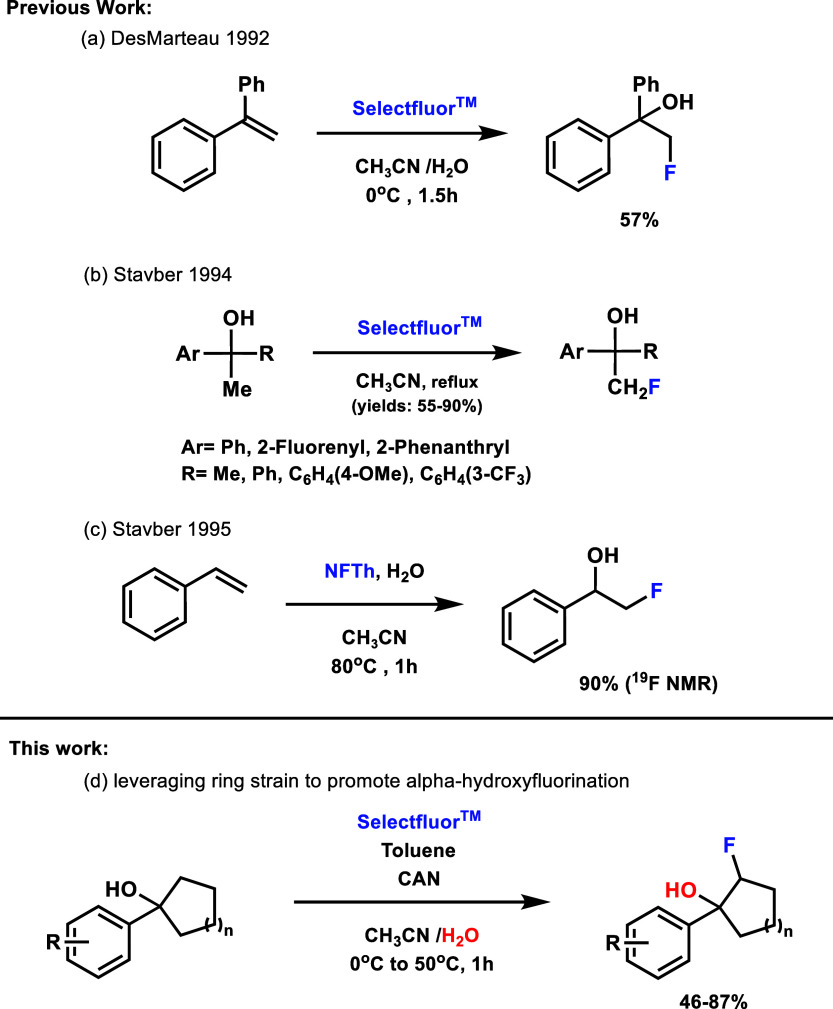
Previous reports on fluorohydrin formation using electrophilic fluorine sources starting from alkenes and alcohols, along with our serendipitously discovered system.

Our investigation intersects and expands upon this early observation and led to our development of a broad fluorohydrin formation from 5-, 6-, 7-, and 8-member cyclic alcohols (Figure 2). Fortuitous screening toward ring-opening fluorination revealed the presence of a Lewis acid additive to be critical for the reaction of unstrained 6-member ring alcohols, and the addition of toluene to be broadly beneficial for product formation. These observations, in combination with pronounced aromatic ring substitution effects, intermediate studies, and trapping experiments, strongly support the fluorohydrin formation occurring via an ionic dehydration/hydroxyfluorination mechanism. This result is in contrast to the radical mechanism we observed for ring-opening fluorination of cyclobutanols and cyclopropyl alcohols and supports ring strain to be a key differentiator in the fluorination reactions of cyclic alcohols.

Our initial study toward fluorohydrin synthesis began with the 7-member cyclic alcohol 1-(4-ethylphenyl)­cycloheptan-1-ol (**1a**), where we found that treatment with Selectfluor in the presence of toluene in mixed acetonitrile/water solvent at 50 °C formed the desired fluorohydrin in 77% yield (Table 1, entry 1). Running the reaction at room temperature produced only trace amounts of the product (Table 1, entry 2), indicating the key role of heat in driving the reaction. Exploring the equivalents of Selectfluor slowed the reaction slightly (Table 1, entries 3–4). Increasing the reaction concentration increased the reaction yield to 82%, while decreasing the concentration lowered product yields (Table 1, entries 5–6). While CAN was not necessary for the reaction, adding 1.1 equiv drove the reaction forward, yielding 91% (Table 1, entry 7). Removal of the toluene additive was found to impede the reaction (66% vs 77% yield, Table 1, entries 8 and 1), and observation of reaction products suggests that toluene promotes alcohol dehydration, as dehydrated alkene is a significant side product in its presence. Attempting to use toluene as a solvent or altering the equivalents (see Table S4 in SI) appeared to impede the reaction, leading to 2.0 equiv being used for subsequent reactions.

**Table 1:**
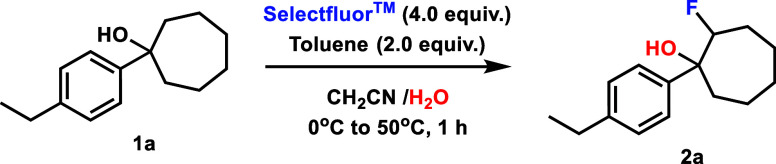
Fluorination Reaction Optimization Using 1-(4-Ethylphenyl)­cycloheptan-1-ol **1a** ^a^

Entry	Variation from Standard Conditions	Yield^b^ (%)
1	None	77 (63^c^)
2	RT, 2 h	trace
3	Selectfluor (2.0 equiv)	47
4	Selectfluor (8.0 equiv)	59
5	0.25 M	82
6	0.0625 M	28
7	1.1 equiv. CAN added	91
8	No toluene added	66

a Reactions were conducted on the 0.06 or 0.1 mmol scale. They were stirred for 10 min at 0 °C, then heated to 50 °C and stirred for 1 h.

b Yields were determined by ^1^H NMR spectroscopy using 1,3,5-trimethoxybenzene as an internal standard and represent a combination of diastereomers.

c The product was isolated through preparative TLC (10% ethyl ether/hexanes).

1-(4-Ethylphenyl)­cyclooctan-1-ol (**3a**) was then synthesized to explore whether these same reactivity trends would be observed for 8-membered ring systems. The behavior of the reaction was largely analogous, generating fluorohydrin product **4a** in 73% yield under the standard conditions (Figure 3). The main point of divergence between these systems is that the 8-membered ring reaction reached completion in 1 h with no starting material recovered (see Table S2 in SI for further details).

**Figure 3:**
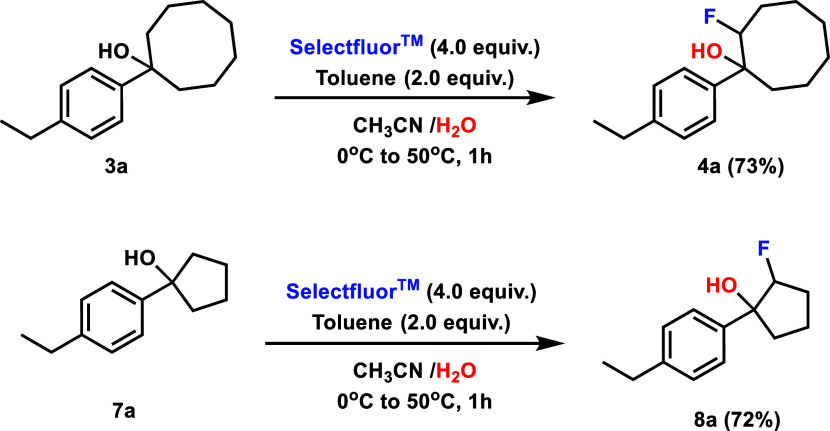
Efficient fluorohydrin synthesis was possible for both 8- and 5-membered ring alcohols using the same conditions as those used for 7-membered ring alcohols. Yields were determined by ^1^H NMR spectroscopy using 1,3,5-trimethoxybenzene as an internal standard and represent a combination of diastereomers.

Next, we wondered whether the absence of significant ring strain in 6-membered rings might change the amenability of these systems to fluorohydrin formation. Beginning with the 6-membered ring alcohol 1-(4-ethylphenyl)­cyclohexan-1-ol (**5a**), we discovered that conditions akin to our CAN-mediated ring-opening fluorination^3^ led exclusively to fluorohydrin **6a** formation in 70% yield (Table 2, entry 1). Interestingly, the reaction would not proceed without CAN present (Table 2, entry 2), a result in contrast to the fluorohydrin formation of 7- and 8-membered ring alcohols. Altering the equivalents of Selectfluor had a stronger impact on the yield in this more stable ring system, decreasing the reaction yield markedly with both higher and lower loadings (Table 2, entries 3–4). A similar effect was observed for reaction concentration, with both raising and decreasing this parameter leading to decreased product formation (Table 2, entries 5–6). Similar to the 7- and 8-membered ring systems, the yield was again increased with the inclusion of a small amount of toluene (Table 2, entry 7).

**Table 2:**
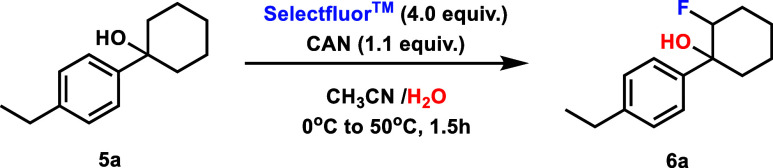
Fluorination Reaction Optimization Using 1-(4-Ethylphenyl)­cyclohexan-1-ol **5a** ^a^

Entry	Variation from Standard Conditions	Yield^b^ (%)
1	None	70
2	No CAN	0
3	Selectfluor (2.0 equiv)	35
4	Selectfluor (8.0 equiv)	62
5	0.25 M	34
6	0.0625 M	27
7	2.0 equiv. toluene added	75

a Reactions were conducted on the 0.025 mmol scale. They were stirred for 10 min at 0 °C then heated to 50 °C and stirred for 1 h.

b Yields were determined by ^1^H NMR spectroscopy using 1,3,5-trimethoxybenzene as an internal standard and represent a combination of diastereomers.

Following this result from the 6-membered ring system, we hypothesized that 5-membered ring alcohols would behave more similarly to the 7-membered ring case due to the comparable ring strain of these two systems (6.5 vs 6.3 kcal/mol).^9^ Putting this theory to the test, we subjected 1-(4-ethylphenyl)­cyclopentan-1-ol (**7a**) to standard conditions analogous to the cyclooctanol system and observed a comparable 72% yield of fluorohydrin product **8a** in 1 h of reaction (Figure 3, see Table S1 in SI for additional details). Interestingly, CAN again proved unnecessary for product formation in this case, leaving unstrained 6-membered ring alcohols as the only substrate class requiring this additive for product formation and suggesting ring strain to be important for this divergence of reactivity.

### Reaction Scope Study

With conditions for fluorohydrin formation across 5- to 8-membered ring alcohols, we next proceeded to examine the substrate scope and the effect of aryl group substituents and ring size on the reaction (Table 3). Reaction efficiency was strongly affected by aryl substitution, with electron-donating substituents in the *para* position (**2a**, **2d**, **4a**, **4d**, **6a**, **6d**, **8a**, and **8d**) leading to efficient fluorohydrin formation. In the case of exceptionally donating *para*-methoxy substitution (**2d**, **4d**, **6d**, and **8d**), the reaction proceeded rapidly at room temperature across all ring sizes and overreacted when heated, yielding many unidentified fluorinated side products. In addition, these strongly activated starting materials demonstrated some product loss due to competitive ring-opening aldehyde formation when reacted with CAN (see the SI). Similarly, *para* electron-withdrawing groups led to a decrease in product formation, with 1-(4-(trifluoromethyl)­phenyl)­cycloheptan-1-ol **1e** only able to form a 34% yield of product **2e** after adding CAN and increasing the reaction time to 24 h. Electron-neutral and weakly donating substrates (**2b**, **2c**, **4b**, **4c**, **6b**, **6c**, **8b**, and **8c**) led to slightly reduced product formation compared to the electron-donating examples, while an attempt to react a tertiary alcohol with no aryl substitution (**5e**) was unsuccessful.

**Table 3:**
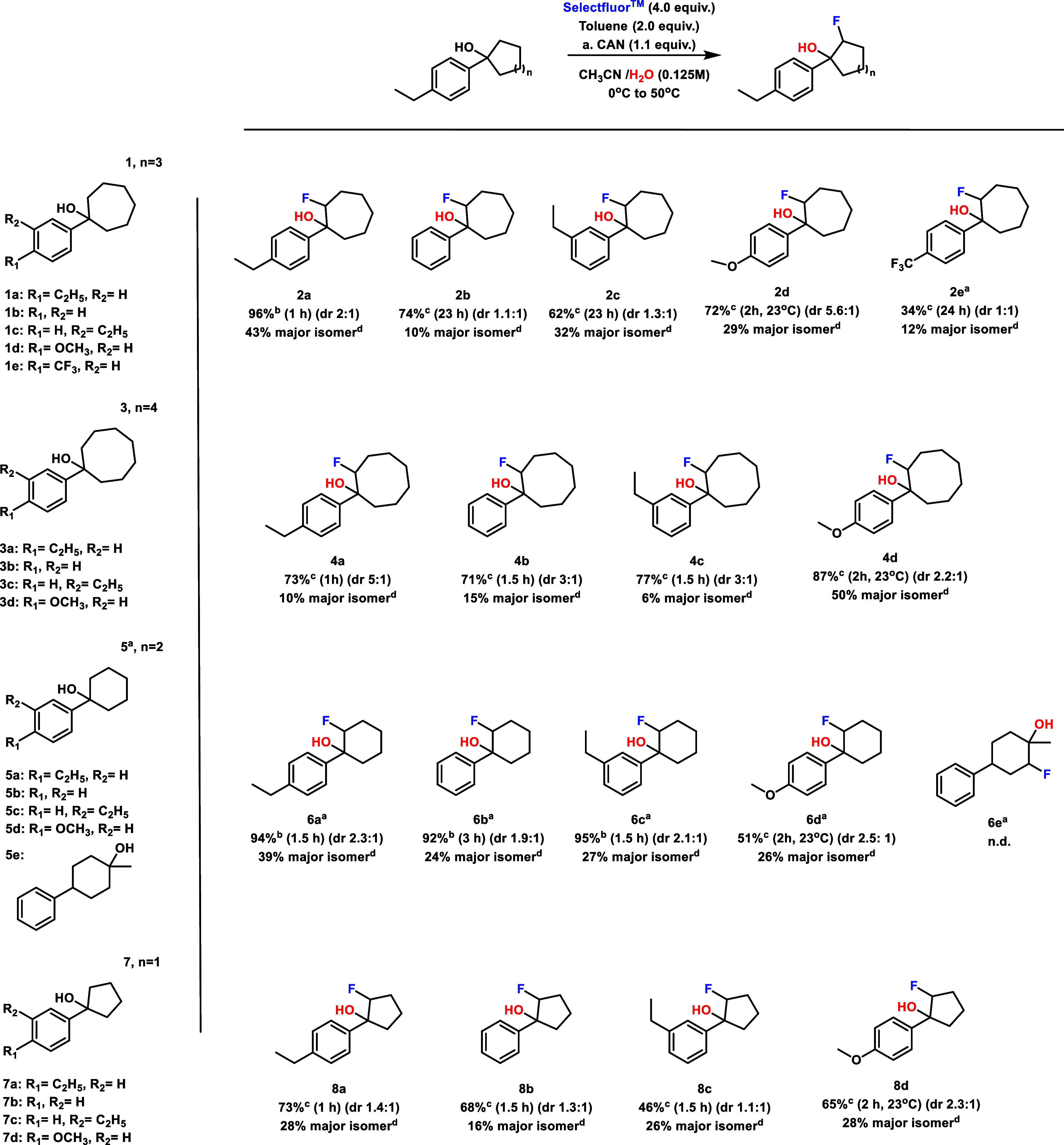
Scope of Cyclic Fluorohydrin Formation^a^ ^b^ ^c^ ^d^

a Reactions requiring CAN (1.1 equiv).

b The isolated yield of combined diastereomers.

c Yield determined by ^1^H NMR spectroscopy using 1,3,5-trimethoxybenzene as an internal standard and represents a mixture of two diastereomers.

d The isolated yield of the major diastereomer.

Finally, the reaction of 1.0 mmol of 1-(4-ethylphenyl)­cycloheptan-1-ol **1a** resulted in a comparable isolated yield of fluorohydrin **2a** (81%), suggesting that the reaction might be performed successfully on larger scales.

### Mechanistic Investigation

We next sought to better understand the dramatic mechanistic divergence leading to fluorohydrin formation for 5- to 8-membered rings compared to the ring-opening fluorination of more highly strained 3- and 4-membered ring alcohols under equivalent conditions. Given that the ring-opening fluorination is known to have significant radical character, we first investigated the likelihood of radical reactivity through the inclusion of known radical inhibitors 2,2,6,6-Tetramethylpiperidine-N-oxyl (TEMPO) and 2,4-di-*tert*-butyl-4-methylphenol (BHT). Reactions of alcohol **3a** appeared inhibited with TEMPO but functioned essentially unchanged with BHT (Figure 4a), providing mixed support for a radical mechanism. We were further suspicious of whether these results truly supported a radical process, given the ability of TEMPO to inhibit ionic reactions as well in some cases. Our skepticism was further increased by the beneficial effect of toluene on the reaction, as alkyl benzenes are known to form benzyl fluorides in the presence of free radicals capable of HAT and Selectfluor,^22^ without the formation of benzyl fluoride. Considering these results (Figure 4a), we decided to explore an alternate ionic pathway, especially as ionic reactivity was previously proposed by Stavber and DesMarteau for the alkene hydroxyfluorination reactivity seen using electrophilic fluorinating reagents (Figure 2).^11,19–21^

**Figure 4:**
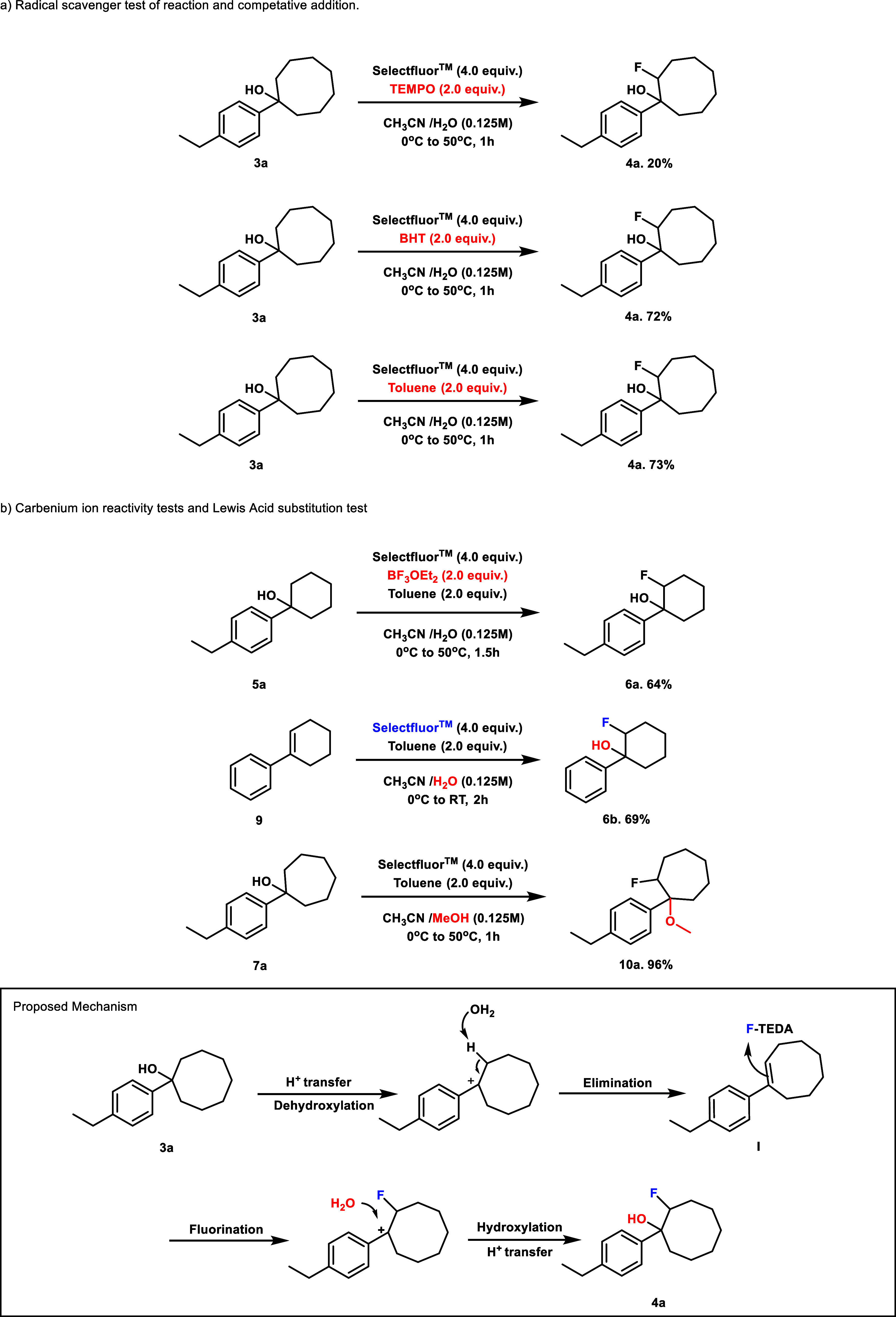
Preliminary mechanistic studies support an ionic elimination/hydroxyfluorination mechanism. Yields were determined by ^1^H NMR spectroscopy using 1,3,5-trimethoxybenzene as an internal standard.

The reaction may instead proceed ionically through elimination and subsequent hydroxyfluorination (Figure 4, proposed mechanism). In this mechanism, an acidic solution would protonate the alcohol, allowing ionization via the loss of water. The carbocation created would then be quenched by the removal of a proton on the neighboring carbon, leading to the elimination product **I**. A reaction of the alkene intermediate with electrophilic fluorine would regenerate the carbocation, which could then be quenched by the addition of water, which, after proton exchange, would lead to our final Markovnikov product **4a.** In accordance with the reactivity demonstrated by the Capilato group, this fluorination occurs at the less substituted position.^23^

To test this potential ionic pathway, we first examined the role of CAN. Though demonstrated to be necessary in the unstrained cyclohexanol rings, removing it from strained 5-, 7-, and 8-membered ring systems had little effect. We thus theorized that CAN may function as a Lewis acid to accelerate the dehydration step in the 6-membered ring system. We proceeded to exchange CAN for an alternate Lewis acid BF_3_OEt_2_ in the reaction of 1-(4-ethylphenyl)­cyclohexan-1-ol (**5a**) under otherwise identical standard reaction conditions (Figure 4b) obtaining comparable yields. Additional support for the role of CAN was obtained through subjecting the proposed dehydrated intermediate 2,3,4,5-tetrahydro-1,1’-biphenyl to reaction conditions without CAN, resulting in hydroxyfluorination proceeding smoothly to yield 69% of product **6a** at room temperature. Together, these data are consistent with CAN functioning as a Lewis acid to aid in the dehydration of unstrained 6-membered ring alcohols.

Having determined a probable role for CAN consistent with our proposed ionic pathway, we next sought to gain further support for the intermediacy of the carbocation intermediates. We first replaced water with methanol as a cosolvent and obtained the methoxy addition product **10a** in an excellent 96% isolated yield (Figure 4b). This result is consistent with nucleophilic solvent attack on a carbocation intermediate and correlates well with prior experimentation by Stavber toward understanding alkene hydroxyfluorination.^21^ Additional support for the presence of carbocation intermediates was found through the attempted elaboration of the substrate scope. 1-Methyl-4-phenylcyclohexan-1-ol (Table 3, **5e**), a tertiary alcohol without direct substitution of an aromatic ring, was unreactive under our conditions, a result consistent with the need for an aromatic moiety to stabilize a carbocation intermediate. This supposition is further supported by the ease of reaction found in compounds containing aryl groups with electron-donating capabilities (**1d**, **3d**, **5d**, and **7d**) contrasting with the sluggishness of electron-withdrawing groups (**1e**). Together, these results are consistent with the observed formal C–H fluorination proceeding via an ionic tandem dehydration/hydroxyfluorination reaction that is promoted by moderate ring strain and/or Lewis acid additives.

## Conclusion

In conclusion, we have demonstrated a simple method for directly producing fluorohydrins from alcohol precursors. Proceeding under mild conditions, ranging from room temperature to 50 °C and tolerating ambient air, this reaction offers a modular approach to installing fluorine while maintaining the versatile alcohol functional group handle. Intriguingly, these fluorohydrin-forming conditions are nearly identical to ring-opening fluorination conditions previously developed for cyclopropyl and cyclobutyl rings, suggesting ring strain to be a key determinant of the reaction outcome. Preliminary mechanistic studies support an ionic dehydration/hydroxyfluorination cascade occurring for cyclic alcohols with moderate ring strain, as opposed to the radical ring-opening reaction of cyclopropyl and cyclobutyl systems, with the unstrained cyclohexyl system requiring further Lewis acid activation to proceed. Together, these results support ring strain as an important parameter for designing cyclic alcohol functionalization reactions that can allow complementary product classes to be accessed.

## Supplementary Material



## Data Availability

The data underlying this study are available in the published article and its online Supporting Information.

## Notes

Experimental procedures, tables of additional experiments, and NMR spectra of all new products (PDF)
