# Supplementary material for: Fluorohydrin Synthesis via Formal C–H Fluorination of Cyclic Alcohols
Source: J Org Chem. 2026 Jan 13;91(4):1848–55. doi: 10.1021/acs.joc.5c02401 (PMC12865773; doi:10.1021/acs.joc.5c02401)
Supplement: Supplementary file 1 [file jo5c02401_si_001.pdf]

Supporting Information

Fluorohydrin Synthesis via Formal C–H Fluorination of Cyclic Alcohols

Helen M.J. Edens, Julian G. West*

Department of Chemistry, Rice University, Houston, Texas, 77005, United States

Table of Contents

Materials and Instrumentation **S1**

Tables of Additional Experiments **S2**

Preparation of Starting Material via Grignard Reactions **S6**

Standard Procedure **S6**

Characterization of Cyclic Alcohols **S7**

Fluorination of Cyclic Alcohols **S13**

Standard Fluorination Procedure **S13**

Characterization of Fluorinated Cyclic Alcohols **S14**

Characterization of Methoxy Addition Products **S23**

Characterization of Isolated Ring Opened Aldehydes **S24**

NMR spectra **S25**

References **S75**

Materials and Instrumentation

Reagents were purchased from commercially available sources and were used without purification unless otherwise noted. All reactions were run in an open atmosphere unless otherwise noted. Reactions noted as being run under nitrogen used degassed solvents and positive N_2 pressure. Reactions were monitored by ^{1}H NMR. TLC was performed using either 0.25 mm pre-coated silica plates (F-254) purchased from Silicycle or neutral aluminum oxide 60 F_{254} from millipore sigma. TLC was visualized using shortwave UV light and/or KMnO4 as a developing agent. Prep TLC was performed using either SiliCycle glass backed silica prep TLC plates, Supelco glass backed silica plates, or Sorbent Technologies Neutral Alumina plates with UV254. Flash column chromatography was performed using SiliaFlash-P60 silica gel (40 – 63 μm) purchased from Silicycle or Teledyne basic alumina Redisep Columns purchased from Fisher Scientific. ^{1}H, ^{13}C and ^{19}F NMR spectra were taken on Bruker DRX-600 spectrometers operating at 600 MHz for proton nuclei, 151 MHz for carbon, and 565 MHz for fluorine. Results were calibrated using residual undeuterated solvent as an internal reference (C_6D_5H: 7.16 ppm ^{1}H NMR and 128.0 ppm ^{13}C NMR) or

(CHCl$_3$: 7.26 ppm 1 H NMR and 77.20 ppm 13C NMR). 1,3,5-trimethoxybenzene was used as standard (C_6D_5H: 6.25 ppm (s, 3H), 3.32 ppm (s, 9H)) for NMR yield calculations.

Tables of Additional Experiments

Table S1. Fluorination Reaction Optimization Using 1-(4-ethylphenyl)cyclopentan-1-ol (7a)[a]

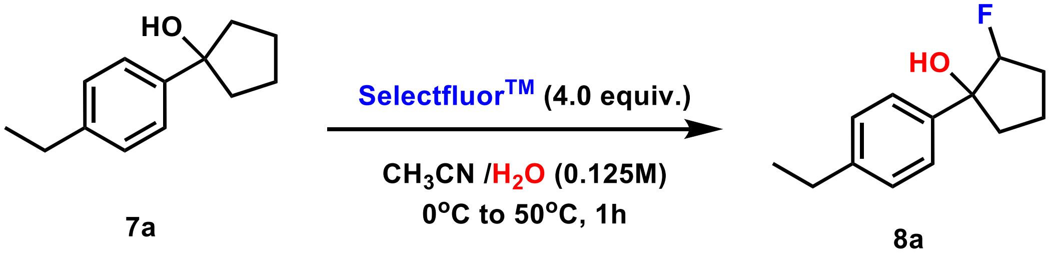

Entry	Variation from Standard Conditions	Yield[b] (%)
1	None	73
2	Selectfluor™ (2.0 equiv.)	72
3	Selectfluor™ (8.0 equiv.)	72
4	0.25M	73
5	0.0625M	76
6	CAN (1.1 equiv.)	62
7	TEMPO (2.0 equiv.)	0
8	BHT (2.0 equiv.)	78
9	Toluene (2.0 equiv.)	72
10	CH_3CN/MeOH (1:1)	78

[a] Reactions were conducted on the 0.06 mmol scale. They were stirred for 10 min at 0ºC then heated to 50 ºC and stirred for 1 hour. [b] Yields were determined by ^{1}H NMR spectroscopy using 1,3,5-trimethoxybenzene as an internal standard and represent a combination of diastereomers.

Table S2. Fluorination Reaction Optimization Using 1-(4-ethylphenyl)cyclooctan-1-ol (3a)[a]

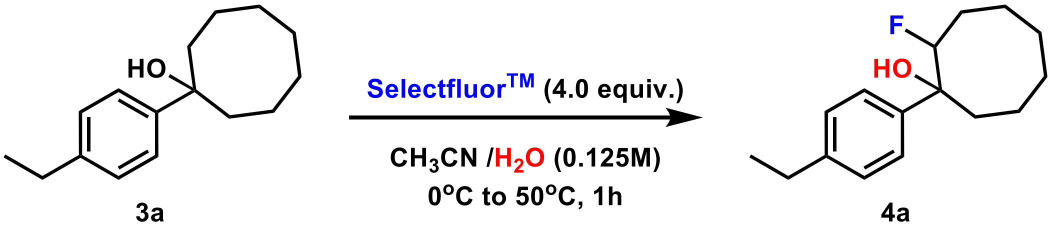

3a 4a

Entry	Variation from Standard Conditions	Yield[b] (%)
1	None	68
3	Selectfluor™ (2.0 equiv.)	70
4	Selectfluor™ (8.0 equiv.)	59
5	0.25M	64
6	0.0625M	64
7	CAN (1.1 equiv.)	61
8	TEMPO (2.0 equiv.)	20
9	BHT (2.0 equiv.)	72
10	Toluene (2.0 equiv.)	73
11	CH_3CN/MeOH (1:1)	36

[a] Reactions were conducted on the 0.1 mmol scale. They were stirred for 10 min at 0ºC then heated to 50 ºC and stirred for 1 hour. [b] Yields were determined by ^{1}H NMR spectroscopy using 1,3,5-trimethoxybenzene as an internal standard and represent a combination of diastereomers.

Table S3. Flourination Reaction Optimization Using 1-(4-methoxyphenyl)cyclopentan-1-ol (7d)[a]

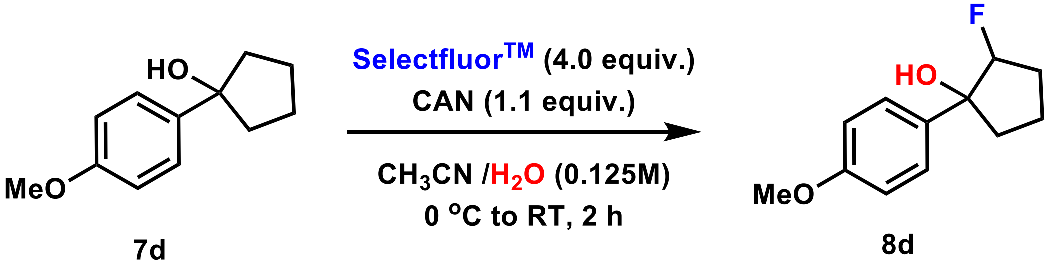

7d 8d

Entry	Variation from Standard Conditions	Yield[b] (%)
1	None	25
3	TEMPO (2.0 equiv.)	12
4	BHT (2.0 equiv.)	40
5	Toluene (1.0 equiv.)	40[c], 22[d]

[a] Reaction conditions: Substrate (0.06 mmol), CAN (0.07 mmol), Selectfluor™ (0.24 mmol), MeCN: H_2O (0.24 mL: 0.24 mL), 0 ºC for 10 min then gradually warmed to RT and run for 2 h. [b]Yields were determined by ^{1}H NMR spectroscopy using 1,3,5-trimethoxybenzene as an internal standard and represent a combination of diastereomers. [c] Yield is an average of two runs. [d] Isolated via column chromatography 15% EA/Hexanes.

Table S4. Fluorination Reaction Optimization using 1-(4-methoxyphenyl)cyclohexan-1-ol (5d)[a]

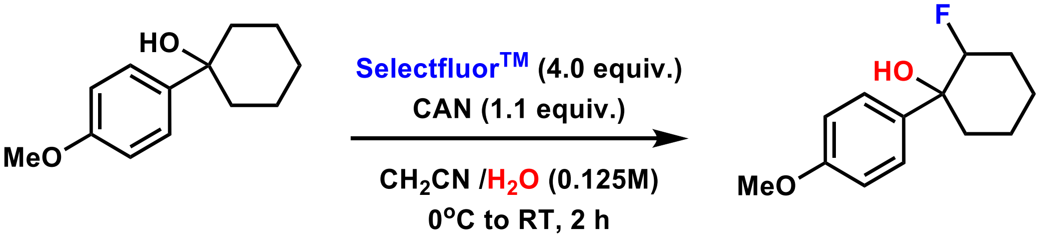

5d

6d

Entry	Variation from Standard Conditions	Yield[b] (%)
1	none	25[c]
2	HFIP/H_2O (1:1)	3
3	Mn (III) acetate (0.1 equiv.)	2
4	Mn (III) acetate (0.1 equiv.); HFIP/H_2O (1:1)	0
5	60°C	0
6	Degassed; run under N_2	11
7	Selectfluor™ (2.0 equiv.)	16
8	No CAN	3
9	Toluene (1.0 equiv.)	26
10	Toluene (2.0 equiv.)	51
11	Toluene (3.0 equiv.)	29
12	Toluene/ H_2O (1:1)	2
13	BF_3OEt_2 (2.0 equiv.) instead of CAN	33
14	Fe(II) Fluoride (1.1 equiv.)	4
15	Zn Acetate (1.1 equiv.)	4
16	Cu(II) Acetylacetonate (1.1 equiv.)	5
17	Mn (II) Acetate tetrahydrate (1.1 equiv.)	3
18	TFA (0.7 equiv.)	13

[a] Reactions were conducted on the 0.06 mmol scale, 0 ºC for 10 min then gradually warmed to RT and run for 2 h. [b] Yields were determined by ^1H NMR spectroscopy using 1,3,5-trimethoxybenzene as an internal standard and represent a combination of diastereomers. [c] Product was isolated through prep TLC (15% ethyl acetate/hexanes).

Table S5. Fluorination Reaction Optimization Using 1-(4-methoxyphenyl)cycloheptan-1-ol (1d)[a]

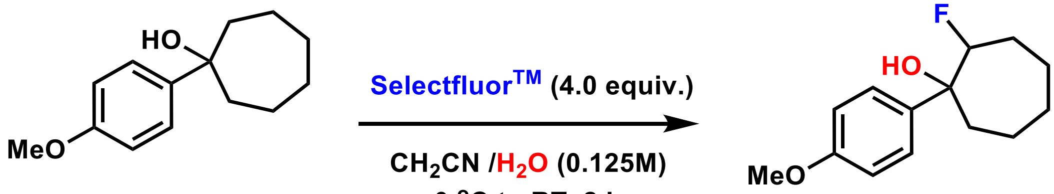

1d

2d

Entry	Variation from Standard Conditions	Yield[b] (%)
1	none	55, 41[c]
2	CAN (1.1 equiv.)	42
3	CAN (1.1 equiv.); HFIP/H_2O (1:1)	9
4	Mn (III) acetate (0.1 equiv.)	36
5	Mn (III) acetate (0.1 equiv.); HFIP/H_2O (1:1)	16
6	CAN (1.1 equiv.); 60°C	0
7	CAN (1.1 equiv.); Degassed; run under N_2	58
8	Selectfluor™ (1.0 equiv.)	50+SM
11	Selectfluor™ (2.0 equiv.)	48+SM
12	Selectfluor™ (2.0 equiv.); 3h	54
13	Selectfluor™ (3.0 equiv.)	51
14	Selectfluor™ (6.0 equiv.)	38

[a] Reactions were conducted on the 0.06 mmol scale, 0 ºC for 10 min then gradually warmed to RT and run for 2 h. [b] Yields were determined by ^{1}H NMR spectroscopy using 1,3,5-trimethoxybenzene as an internal standard and represent a combination of diastereomers. [c] Product was isolated through prep TLC (15% ethyl acetate/hexanes).

Table S6. Fluorination Reaction Optimization Using 1-(4-methoxyphenyl)cyclooctan-1-ol (3a)[a]

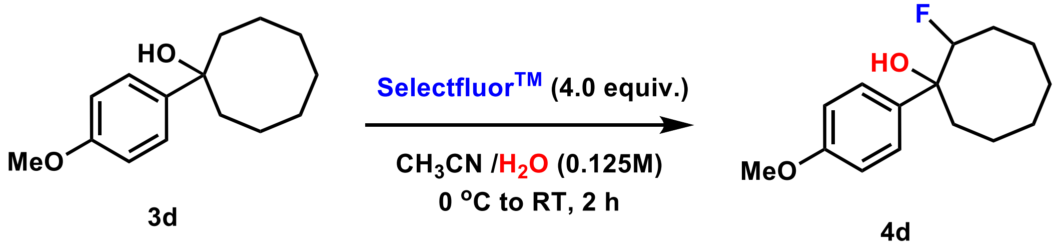

Entry	Variation from Standard Conditions	Yield[b] (%)
1	none	56, 48[c]
2	HFIP/H_2O (1:1)	0
3	CAN (1.1 equiv.); 60 °C	0
4	CAN (1.1 equiv.); Degassed, run under N_2	45
5	CAN (1.1 equiv.); Selectfluor™ (6.0 equiv.)	52
6	Toluene (1.0 equiv.)	60[d]
7	TEMPO (2.0 equiv.)	36

[a] Reaction conditions: Substrate (0.06 mmol), Selectfluor™ (0.24 mmol), MeCN: H_2O (0.24 mL: 0.24 mL), 0 ºC for 10 min then gradually warmed to RT and run for 2 h. [b] Yields were determined by ^1H NMR spectroscopy using 1,3,5-trimethoxybenzene as an internal standard and represent a combination of diastereomers. [c] Isolated via column chromatography 15% EA/Hexanes. [d] Average of two runs.

Preparation of Starting Material via Grignard Reactions

Standard Procedure

Grignard Reaction

A two-neck oven dried round bottom flask equipped with stir bar and septum was flame dried under high vacuum then allowed to cool under N_2. Ketone (8.5 mmol, 1.0 eq) was then added along with THF (43.5 mL). Mg chips (27.2 mmol, 1.6 eq) were then placed under N_2 in a separate round bottom flask equipped with stir bar, and septum. A final 20 mL vial with septum lid was then evacuated and filled with N_2 to which aryl bromide (17.0 mmol, 2.0 eq) was added. THF (15.0 mL) was then split evenly between the Mg chips and the aryl bromide vial. 1-3 drops of dibromoethane were then added to the Mg chip flask and a small amount of the aryl bromide dissolved in THF. The reaction became slightly warm and the remainder of the aryl bromide was added in small portions. Once the solution became cloudy and was no longer warm to the touch it was added to the three-neck flask. Once reaction completion was verified by NMR, the reaction was quenched with saturated ammonium chloride. The resulting mixture was then diluted with ethyl acetate, extracted three times with 20.0 mL portions of ethyl acetate, washed 3x with 20.0 mL portions of water then brine, then dried with sodium sulfate, and concentrated under vacuum. Product was purified via column chromatography using a basic aluminum oxide column and an ethyl ether/hexanes solvent system.

Characterization of Cyclic Alcohols

1-(4-ethylphenyl)cycloheptan-1-ol (1a)

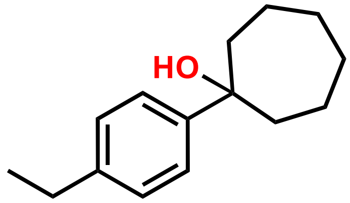

Synthesized according to Grignard reaction method using 1-bromo-4-ethylbenzene (2.4 mL, 17 mmol, 2.0 eq), cycloheptanone (1.0 mL, 8.5 mmol, 1.0 eq), and magnesium chips (661.2 mg, 27.2 mmol, 1.6 eq) with 2-3 drops dibromoethane as initiator. Mixture was purified via CombiFlash on Basic Aluminum Oxide 10% ethyl acetate in hexanes.
Colorless oil; **Mass:** 1.32 g **Yield:** 71.0% **R*f*:** 0.39 (20% Ethyl Ether/Hexanes). **^{1}H NMR (600MHz, C_6D_6):** δ 7.42 (d, J= 7.9 Hz, 2H), 7.10 (d, J= 7.9 Hz, 2H), 2.51 (q, J= 7.6 Hz, 2H), 1.91 (dd, J= 8.4 Hz, 2H), 1.82-1.72 (m, 4H), 1.61 (br s, 2H), 1.49-1.38 (m, 5H), 1.14 (t, J= 7.6 Hz, 3H). **^{13}C{^{1}H} NMR (150MHz, C_6D_6):** δ 148.8, 141.9, 127.5, 124.7, 76.1, 43.3, 29.0, 28.4, 22.4, 15.7. **HRMS** (ESI) *m/z*: [M+Na]+ Calcd for $C_{15}H_{22}ONa$: 241.1563, found: 241.1561.

1-phenylcycloheptan-1-ol (1b)[1]

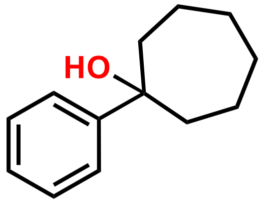

Synthesized according to Grignard reaction method using bromobenzene (1.81 mL, 17 mmol, 2.0 eq), cycloheptanone (1.0 mL, 8.5 mmol, 1.0 eq), and magnesium chips (661.2 mg, 27.2 mmol, 1.6 eq) with 2-3 drops dibromoethane as initiator. Mixture was purified via CombiFlash on Basic Aluminum Oxide 10% ethyl acetate in hexanes.
Colorless oil; **Mass:** 1.19 g **Yield:** 73.8% **R*f*:** 0.36 (20% Ethyl Ether/Hexanes). **^{1}H NMR (600MHz, C_6D_6):** δ 7.44 (d, J= 7.8 Hz, 2H), 7.21 (t, J= 7.7 Hz, 2H), 7.10 (t, J= 7.3 Hz, 1H), 1.86 (t, J= 12.7 Hz, 2H), 1.76-1.70 (m, 4H), 1.60 (br s, 2H), 1.47-1.39 (m, 4H). **^{13}C{^{1}H} NMR (150MHz, C_6D_6):** δ 151.4, 126.2, 124.6, 76.21, 43.1, 28.9, 22.4. **HRMS** (ESI) *m/z*: [M-H_2O+H]+ Calcd for $C_{13}H_{17}$: 173.1325, found: 173.1324. This data is analogous to reported literature.[1]

1-(3-ethylphenyl)cycloheptan-1-ol (1c)

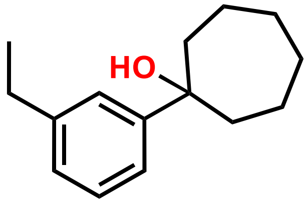

Synthesized according to Grignard reaction method using 1-bromo-3-ethylbenzene (2.3 mL, 17.0 mmol, 2.0 eq), cycloheptanone (1.0 mL, 8.5 mmol, 1.0 eq), and magnesium chips (667.0 mg, 27.2 mmol, 1.6 eq) with 2-3 drops dibromoethane as initiator. Mixture was purified via CombiFlash on Basic Aluminum Oxide 10% ethyl acetate in hexanes.
Colorless oil; **Mass:** 1.01 g **Yield:** 54.4% **R*f*:** 0.36 (20% Ethyl Ether/Hexanes). **^{1}H NMR (600MHz, C_6D_6):** δ 7.42 (s, 1H), 7.29 (d, J= 7.8 Hz, 1H), 7.21 (t, J= 7.6 Hz, 1H), 7.00 (d, J= 7.5 Hz, 1H), 2.55 (q, J= 7.6 Hz, 2H), 1.91 (ddd, J= 14.9, 10.6, 3.9 Hz, 2H), 1.80-1.72 (m, 4H), 1.64-1.57 (m, 2H), 1.49-1.41 (m, 4H), 1.16 (t, J= 7.6 Hz, 3H), 1.13-1.10 (br d, 1H). **^{13}C{^{1}H} NMR (150MHz, C_6D_6):** δ 151.6, 143.7, 128.1, 125.8, 124.1, 122.0, 76.1, 43.3, 29.2, 28.8, 22.4, 15.7. **HRMS** (ESI) *m/z*: [M-H_2O+H]- Calcd for $C_{15}H_{21}$: 201.1638, found: 201.1636.

1-(4-methoxyphenyl)cycloheptan-1-ol (1d)[2]

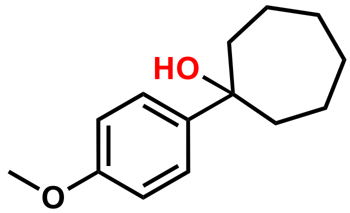

Synthesized according to Grignard reaction method using 4-bromoanisole (600mg, 3.21 mmol, 1.5 eq), cycloheptanone (180 mg, 2.14 mmol, 1.0 eq), and magnesium chips (85.8 mg, 3.53 mmol, 1.65 eq) with 2-3 drops dibromoethane as initiator. Mixture was purified via CombiFlash on Silica gel with15% ethyl acetate in hexanes.
^{1}H NMR (600MHz, $CDCl_3$): δ 7.42 (d, J= 8.6 Hz, 2H), 6.87 (d, J= 8.6 Hz, 2H), 3.80 (s, 3H), 2.07-2.03 (m, 2H), 1.91-1.87 (m, 2H), 1.81-1.76 (m, 2H), 1.73-1.68 (m, 2H), 1.62-1.53 (m, 4H). These results are in accordance with published literature.[2]

1-(4-(trifluoromethyl)phenyl)cycloheptan-1-ol (1e)

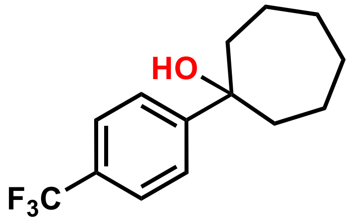

Synthesized according to Grignard reaction method using 4-bromobenzotrifluoride (2.4 mL, 17.0 mmol, 2.0 eq), cycloheptanone (1.0 mL, 8.5 mmol, 1.0 eq), and magnesium chips (661.2 mg, 27.2 mmol, 1.6 eq) with 2-3 drops dibromoethane as initiator. Mixture was purified via CombiFlash on Basic Aluminum Oxide 40% ethyl acetate in hexanes.
Colorless oil; **Mass:** 289.2 mg **Yield:** 13.2% **R*f*:** 0.33 (20% Ethyl Ether/Hexanes). **^{1}H NMR (600MHz, C_6D_6):** δ 7.42 (d, J= 8.3 Hz, 2H), 7.26 (d, J= 8.2 Hz, 2H), 1.67-1.53 (m, 8H), 1.43-1.31 (m, 4H), 1.04-1.03 (d, 1H). **^{13}C{^{1}H} NMR (150MHz, C_6D_6):** δ 155.3, 124.9 (m), 75.9, 42.9, 28.6, 22.2. **^{19}F NMR (565 MHz, C_6D_6):** δ -61.9. **HRMS** (ESI) *m/z*: [M-H_2O+H]- Calcd for $C_{14}H_{16}F_3$: 241.1199, found: 241.1197.

1-(4-ethylphenyl)cyclooctane-1-ol (3a)

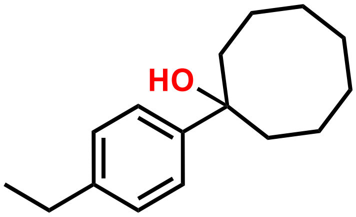

Synthesized according to Grignard reaction method using 1-bromo-4-ethylbenzene (1.8 mL, 13.0 mmol, 1.5 eq), cyclooctanone (1.11 g, 8.7 mmol, 1.0 eq), and magnesium chips (506.6 mg, 20.8 mmol, 1.6 eq) with 2-3 drops dibromoethane as initiator. Mixture was purified via CombiFlash on Basic Aluminum Oxide 10% ethyl acetate in hexanes.

White solid, **Melting Point**: 53.3°C; **Mass:** 1.14 g **Yield:** 56.3% **R*f*:** 0.35 (20% Ethyl Ether/Hexanes). **^{1}H NMR (600MHz, C_6D_6):** δ 7.45 (d, J= 7.3 Hz, 2H), 7.11 (d, J= 7.8 Hz, 2H), 2.51 (q, J= 7.5 Hz, 2H), 1.95 (dd, J= 14.7, 8.5 Hz, 2H), 1.84 (t, J= 12.1 Hz, 2H), 1.69 (br s, 2H), 1.61-1.56 (m, 3H), 1.43-1.40 (m, 5H), 1.14 (t, J= 7.4 Hz, 3H). **^{13}C{^{1}H} NMR (150MHz, C_6D_6):** δ 147.2, 142.1, 127.4, 125.2, 75.8, 37.9, 28.4, 28.3, 24.4, 21.9, 15.6. **HRMS** (ESI) *m/z*: [M-H_2O+H]+ Calcd for $C_{16}H_{23}$: 215.1794, found: 215.1793.

1-phenylcyclooctan-1-ol (3b)[1]

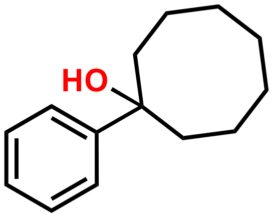

Synthesized according to Grignard reaction method using bromobenzene (1.4 mL, 13 mmol, 1.5 eq), cyclooctanone (730 mg, 5.78 mmol, 1.0 eq), and magnesium chips 510.5 mg, 20.8 mmol, 1.6 eq) with 2-3 drops dibromoethane as initiator. Mixture was purified via CombiFlash on Basic Aluminum Oxide 10% ethyl acetate in hexanes.

^{1}H NMR (600MHz, C_6D_6): δ 7.47 (d, J= 7.9 Hz, 2H), 7.22 (t, J= 7.7 Hz, 2H), 7.11 (t, J= 7.3 Hz, 1H), 1.89 (dd, J= 14.6, 8.5 Hz, 2H), 1.79 (dd, J= 14.7, 9.9 Hz, 2H), 1.71-1.65 (m, 2H), 1.61-1.53 (m, 3H), 1.42-1.37 (m, 5H), 1.14 (s, 1H). **^{13}C{^{1}H} NMR (150MHz, C_6D_6):** δ 149.9, 127.9, 126.4, 125.1, 75.8, 37.9, 28.3, 24.3, 21.8. **HRMS** (ESI) *m/z*: [M-H_2O+H]-Calcd for $C_{14}H_{19}$: 187.1481, found: 187.1480. This data is analogous to reported literature.[1]

1-(3-ethylphenyl)cyclooctane-1-ol (3c)

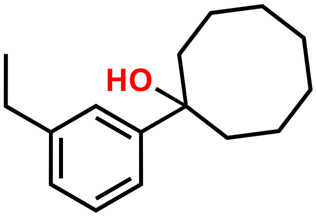

Synthesized according to Grignard reaction method using 1-bromo-3-ethylbenzene (1.2 mL, 8.8 mmol, 1.0 eq), cyclooctanone (1.11 g, 8.7 mmol, 1.0 eq), and magnesium chips (508.1 mg, 20.8 mmol, 2.4 eq to Br) with 2-3 drops dibromoethane as initiator. Mixture was purified via CombiFlash on Basic Aluminum Oxide 10% ethyl acetate in hexanes.
Colorless oil; **Mass:** 392.0 mg **Yield:** 19.3% **R*f*:** 0.42 (20% Ethyl Ether/Hexanes). **^{1}H NMR (600MHz, C_6D_6):** δ 7.46 (s, 1H), 7.31 (d, J= 7.7 Hz, 1H), 7.2 (t, J= 7.6 Hz, 1H), 7.01 (d, J= 7.4 Hz, 1H), 2.55 (q, J= 7.6 Hz, 2H), 1.96 (dd, J= 14.7, 8.6 Hz, 2H), 1.83 (dd, J= 14.6, 10.0 Hz, 2H), 1.74-1.67 (m, 2H), 1.62-1.56 (m, 3H), 1.47-1.39 (m, 5H), 1.16 (t, J= 7.6 Hz, 3H), 1.08 (s, 1H). **^{13}C{^{1}H} NMR (150MHz, C_6D_6):** δ 150.0, 143.7, 125.9, 124.6, 122.5, 75.9, 38.0, 29.2, 28.4, 24.3, 21.9, 15.8. **HRMS** (ESI) *m/z*: [M-H_2O+H]+ Calcd for $C_{16}H_{23}$: 215.1794, found: 215.1793.

1-(4-methoxyphenyl)cyclooctan-1-ol (3d)[2]

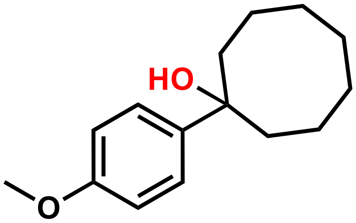

Synthesized according to Grignard reaction method using 4-bromoanisole (600mg, 3.21 mmol, 1.5 eq), cyclooctanone (180 mg, 2.14 mmol, 1 eq), and magnesium chips (85.8 mg, 3.53 mmol, 1.65 eq) with 2-3 drops dibromoethane as initiator. Mixture was purified via CombiFlash on Basic Aluminum Oxide 10% ethyl acetate in hexanes.
^{1}H NMR (600MHz, $CDCl_3$): δ 7.44 (d, J= 8.8 Hz, 2H), 6.88 (d, J= 8.8 Hz, 2H), 3.81 (s, 3H), 2.06-2.02 (m, 2H), 1.98-1.94 (m, 2H), 1.76-1.68 (m, 5H), 1.55-1.50 (m, 6H). These results are in accordance with published literature.[2]

1-(4-ethylphenyl)cyclohexan-1-ol (5a)

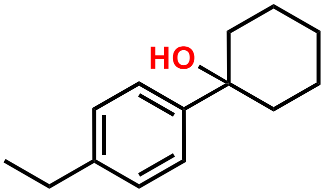

Synthesized according to Grignard reaction method using 1-bromo-4-ethylbenzene (2.35mL, 17 mmol, 2 eq), cyclohexanone (.88mL, 8.5 mmol, 1 eq), and magnesium chips 694.7 mg, 27.2 mmol, 1.6 eq to Br) with 2-3 drops dibromoethane as initiator. Mixture was purified via CombiFlash on Basic Aluminum Oxide 10% ethyl acetate in hexanes.
Colorless oil; **Mass**: 1.26 g **Yield**: 72.5% **R*f*:** 0.43 (20% Ethyl Ether/Hexanes). **^{1}H NMR (600MHz, C_6D_6):** δ 7.40 (d, J=8.2 Hz, 2H), 7.10 (d, J= 8.1 Hz, 2H), 2.50 (q, J= 7.6 Hz, 2H), 1.80-1.73 (m, 2H), 1.67-1.60 (m, 5H), 1.49-1.45 (m, 2H), 1.16-1.09 (m, 5H). **^{13}C{^{1}H} NMR (150MHz, C_6D_6):** δ 147.4, 142.1, 127.5, 72.4, 38.9, 28.4, 25.6, 22.1, 15.6. **HRMS** (ESI) *m/z*: [M-H_2O+H]+ Calcd for $C_{14}H_{19}$: 187.1481, found: 187.1481.

1-phenylcyclohexan-1-ol (5b)[1]

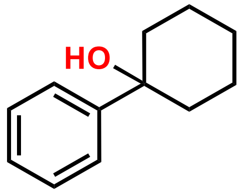

Synthesized according to Grignard reaction method using bromobenzene (1.81 mL, 17 mmol, 2.0 eq), cyclohexanone (.88 mL, 8.5 mmol, 1.0 eq), and magnesium chips 661.2 mg, 27.2 mmol, 1.6 eq to Br) with 2-3 drops dibromoethane as initiator. Mixture was purified via CombiFlash on Basic Aluminum Oxide 10% ethyl acetate in hexanes.
White solid, **Melting Point**: 44.0°C; **Mass**: 1.08 g **Yield**: 69.5% **R*f*:** 0.37 (20% Ethyl Ether/Hexanes). **^{1}H NMR (600MHz, C_6D_6):** δ 7.42 (d, J=7.5 Hz, 2H), 7.22 (t, J= 7.7 Hz, 2H), 7.11 (t, J= 7.3 Hz, 1H), 1.77-1.69 (m, 2H), 1.62-1.60 (m, 5H), 1.46-1.43 (m, 2H). 1.14-1.06 (m, 2H). **^{13}C{^{1}H} NMR (150MHz, C_6D_6):** δ150.0, 126.4, 124.7, 72.5, 38.8, 25.6, 22.1. **HRMS** (ESI) *m/z*: [M-H_2O+H]+ Calcd for $C_{12}H_{15}$: 159.1168, found: 159.1168. This data is analogous to reported literature.[1]

1-(3-ethylphenyl)cyclohexan-1-ol (5c)

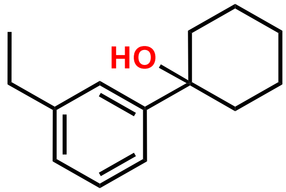

Synthesized according to Grignard reaction method using 1-bromo-3-ethylbenzene (2.33 mL, 17 mmol, 1.5 eq), cyclohexanone (.88 mL, 8.5 mmol, 1.0 eq), and magnesium chips (661.2 mg, 27.2 mmol, 1.6 eq) with 2-3 drops dibromoethane as initiator. Mixture was purified via CombiFlash on Basic Aluminum Oxide 10% ethyl acetate in hexanes.
Colorless oil; **Mass:** 1.35 g **Yield:** 77.5% **R*f*:** 0.43 (20% Ethyl Ether/Hexanes) **^{1}H NMR (600MHz, C_6D_6):** δ 7.39 (s, 1H), 7.28 (d, J= 7.4 Hz, 1H), 7.21 (t, J=7.5 Hz, 1H), 7.00 (d, J= 7.3 Hz, 1H), 2.54 (q, J= 7.6 Hz, 2H), 1.77 (br s, 2H), 1.67-1.62 (m, 5H), 1.47 (br d, 2H, J= 12.7 Hz), 1.17-1.10 (m, 4H). **^{13}C{^{1}H} NMR (150MHz, C_6D_6):** δ 150.2, 143.8, 128.1, 126.0, 124.2, 122.1, 72.7, 38.9, 29.2, 25.6, 22.1, 15.8. **HRMS** (ESI) *m/z*: [M+Na]+ Calcd for $C_{14}H_{20}$ONa: 227.1406, found 227.1404.

1-(4-methoxyphenyl)cyclohexan-1-ol (5d)[2,3]

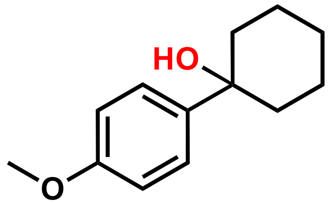

Synthesized according to Grignard reaction method using 4-bromoanisole (600mg, 3.21 mmol, 1.5 eq), cyclohexanone (270 mg, 2.13 mmol, 1.0 eq), and magnesium chips (85.8 mg, 3.53 mmol, 1.65 eq) with 2-

3 drops dibromoethane as initiator. Mixture was purified via CombiFlash on Silica gel with15% ethyl acetate in hexanes.
^{1}H NMR (600MHz, $CDCl_3$): δ 7.43 (d, J= 8.83 Hz, 2H), 6.88 (d, J= 8.83 Hz, 2H), 3.80 (s, 3H), 1.85-1.70 (m, 7H), 1.64-1.61 (m, 2H), 1.53 (s, 1H), 1.32-1.25 (m, 1H). This data matches published literature.[2,3]

1-(4-ethylphenyl)cyclopentan-1-ol (7a)

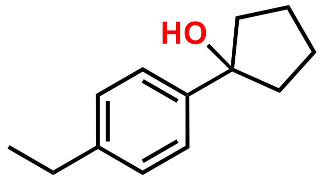

Synthesized according to Grignard reaction method using 1-bromo-4-ethylbenzene (1.79mL, 13 mmol, 1.5 eq), cyclopentanone (.77mL, 8.7 mmol, 1 eq), and magnesium chips 512.8 mg, 20.8 mmol, 1.6 eq to Br) with 2-3 drops dibromoethane as initiator. Mixture was purified via CombiFlash on Basic Aluminum Oxide 10% ether in hexanes.
Colorless oil; **Mass**: 923.8 mg **Yield**: 55.8% **R*f*:** 0.32 (20% Ethyl Ether/Hexanes). **^{1}H NMR (600MHz, C_6D_6):** δ 7.38 (d, J= 7.9, 2H), 7.08 (d, J= 7.9 Hz, 2H), 2.50 (q, J= 7.6Hz, 2H), 1.91 (br s, 2H), 1.83 (br s, 4H), 1.63 (br s, 2H), 1.13 (t, J= 7.61Hz, 3H). **^{13}C{^{1}H} NMR (150MHz, C_6D_6):** δ 144.9, 142.2, 127.5, 125.2, 41.9, 28.5, 23.9, 15.7. **HRMS** (ESI) *m/z*: [M-H_2O+H]+ Calcd for $C_{13}H_{17}$: 173.1325, found: 173.1325.

1-phenylcyclopentan-1-ol (7b)[4]

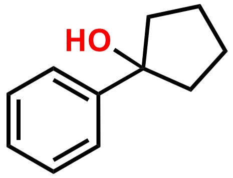

Synthesized according to Grignard reaction method using bromobenzene (1.37mL, 13 mmol, 1.5 eq), cyclopentanone (.77mL, 8.7 mmol, 1 eq), and magnesium chips 505.6 mg, 20.8 mmol, 1.6 eq to Br) with 2-3 drops dibromoethane as initiator. Mixture was purified via CombiFlash on Basic Aluminum Oxide 10% Ethyl Ether in hexanes.
Colorless oil; **Mass**: 914.6 mg **Yield**: 64.8% **R*f*:** 0.32 (20% Ethyl Ether/Hexanes). **^{1}H NMR (600MHz, C_6D_6):** δ 7.40 (d, J= 7.3 Hz, 2H), 7.20 (t, J= 7.7 Hz, 2H), 7.10 (t, J= 7.3 Hz, 1H), 1.93-1.86 (m, 2H), 1.81-1.75 (m, 4H), 1.64-1.57 (m, 2H). **^{13}C{^{1}H} NMR** (150 MHz, **C_6D_6):** δ 147.6, 126.5, 125.1, 82.8, 41.9, 23.9. **HRMS** (ESI) *m/z*: [M-H_2O+H]+ Calcd for $C_{11}H_{13}$: 146.1045, found: 146.1044. This data matches reported literature.[4]

1-(3-ethylphenyl)cyclcopentan-1-ol (7c)

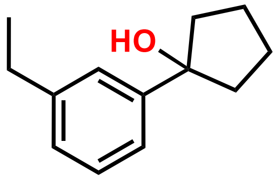

Synthesized according to Grignard reaction method using 1-bromo-3-ethylbenzene (2.33mL, 17 mmol, 2 eq), cyclopentanone (.77mL, 8.7 mmol, 1 eq), and magnesium chips (660.8 mg, 27.2 mmol, 1.6 eq to Br)

with 2-3 drops dibromoethane as initiator. Mixture was purified via CombiFlash on Basic Aluminum Oxide 10% ethyl acetate in hexanes.
Colorless oil; Mass: 389.5 mg **Yield**: 21.5% **R*f*:** 0.39 (20% Ethyl Ether/Hexanes). **^{1}H NMR (600MHz, C_6D_6):** δ 7.37 (s, 1H), 7.26 (d, J= 7.7 Hz, 1H), 7.19 (t, J= 7.6 Hz, 1H), 7.00 (d, J= 7.4 Hz, 1H), 2.53 (q, J= 7.6 Hz, 2H), 1.92 (br s, 2H), 1.83 (br s, 4H), 1.63 (br s, 2H), 1.15 (t, J= 7.6 Hz, 3H). **^{13}C{^{1}H} NMR (150MHz, C_6D_6):** δ 147.6, 143.7, 128.1, 126.0, 124.8, 122.6, 83.0, 42.0, 29.1, 23.9, 15.7. **HRMS** (ESI) *m/z*: [M-H_2O+H]+ Calcd for $C_{13}H_{17}$: 173.1325, found: 173.1325.

1-(4-methoxyphenyl)cyclopentan-1-ol (7d)[2,3]

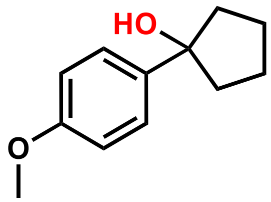

Synthesized according to Grignard reaction method using 4-bromoanisole (600mg, 3.21 mmol, 1.5 eq), cyclopentanone (135 mg, 1.61 mmol, 1.0 eq), and magnesium chips (85.8 mg, 3.53 mmol, 1.65 eq) with 2-3 drops dibromoethane as initiator. Mixture was purified via CombiFlash on Silica gel with 15% ethyl acetate in hexanes.
^{1}H NMR (600MHz, $CDCl_3$): δ 7.42 (d, J= 8.82 Hz, 2H), 6.88 (d, J= 8.82 Hz, 2H), 3.81 (s, 3H), 1.98 (br s, 6H), 1.82 (br s, 2H). This data matches reported literature.[2,3]

Fluorination of Cyclic Alcohols

Standard Fluorination Procedure

In an 8.0 mL vial equipped with stirbar, Selectfluor™ (0.24 mmol, 4.0 eq) was combined with the starting material (0.06 mmol, 1.0 eq) in a MeCN/H_2O (0.24 mL: 0.24 mL) solution. The resulting mixture was then stirred at 0°C for 10 min before the addition of CAN (0.07 mmol, 1.1 eq). The mixture was then either left for 2 h at room temperature or heated to 50°C via sand bath before being washed and extracted with 3 x 1.0 mL portions EA, dried with $NaSO_4$, and concentrated under vacuum. The crude reaction mixture was then assessed via ^{1}H and ^{19}F NMR acquired in C_6D_6. Product was purified via Prep TLC with an ethyl ether/hexanes solvent system (10-15%).

Characterization of Fluorinated Cyclic Alcohols

Two diastereomers are synthesized during the reaction. Where possible, these diastereomers were separated and characterized and experimental data and NMR spectra have been included for each isolated diastereomer and sub labeled "a" or "b" when applicable (e.g. "2aa" and "2ab"). In all other instances, data is reported for the major isomer.

1-(4-ethylphenyl)-2-fluorocycloheptan-1-ol major isomer (2aa)

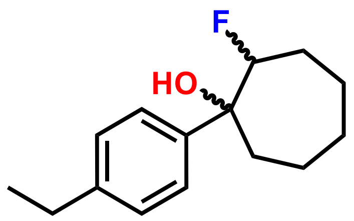

Synthesized according to cyclic fluorination method using (**1a**) (65.5 mg, 0.3 mmol, 1.0 eq) with Selectfluor™ (422.6 mg, 1.2 mmol, 4.0 eq) and Toluene (0.06 mL, 0.6 mmol, 2.0 eq). Purified via prep TLC on neutral aluminum oxide 20% ethyl ether in hexanes.
Colorless Oil; **Mass:** 30.3 mg **Yield:** 42.7% **R*f*:** 0.32 (20% Ethyl Ether/Hexanes). **^{1}H NMR (600MHz, C_6D_6):** δ 7.36 (d, J= 7.9 Hz, 2H), 7.10 (d, J= 7.9 Hz, 2H). 4.67 (dd, J= 45.0, 10.7 Hz, 1H), 2.50 (q, J= 7.6 Hz, 2H), 2.37-2.34 (br m, 1H), 2.19 (q, J= 12.1 Hz, 1H), 1.90-1.72 (m, 3H), 1.60-1.52 (m, 2H), 1.43-1.36 (m, 1H), 1.31-1.11 (m, 6H). **^{13}C{^{1}H} NMR (150MHz, C_6D_6):** δ 145.2, 142.3, 127.6, 124.8, 99.1, 97.9, 76.4, 76.3, 39.0 (d), 28.4, 28.1, 28.0, 26.5, 21.4, 21.3, 19.8, 15.5. **^{19}F NMR (565 MHz, C_6D_6):** δ -176.3 (m). **HRMS** (ESI) *m/z*: [M+Na]+: Calcd for $C_{15}H_{21}FONa$: 259.1469, found: 259.1467.

1-(4-ethylphenyl)-2-fluorocycloheptan-1-ol minor isomer (2ab)

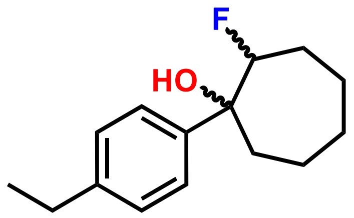

Synthesized according to cyclic fluorination method using (**1a**) (65.5 mg, 0.3 mmol, 1.0 eq) with Selectfluor™ (422.6 mg, 1.2 mmol, 4.0 eq) and Toluene (0.06 mL, 0.6 mmol, 2.0 eq). Purified via prep TLC on neutral aluminum oxide 20% ethyl ether in hexanes.
Colorless Oil; **Mass:** 14.1 mg **Yield:** 19.9% **R*f*:** 0.32 (20% Ethyl Ether/Hexanes). **^{1}H NMR (600MHz, C_6D_6):** δ 7.50 (d, J= 8.0 Hz, 2H), 7.10 (d, J= 8.2 Hz, 2H), 4.57 (dd, J= 46.3, 6.20 Hz, 1H), 2.49 (q, J= 7.6 Hz, 2H), 2.23 (t, J= 13.1 Hz, 1H), 1.97-1.85 (m, 2H), 1.78-1.71 (m, 1H), 1.64-1.52 (m, 3H), 1.46-1.33 (m, 4H), 1.12 (t, J= 7.6 Hz, 3H). **^{13}C{^{1}H} NMR (150MHz, C_6D_6):** δ 144.2, 142.7, 127.3, 126.0, 125.9, 76.8, 76.7, 36.8 (d), 29.3, 29.2, 28.4, 27.2, 21.6 (d), 21.1, 15.5. **^{19}F NMR (565 MHz, C_6D_6):** δ -181.9 (t, J= 40.2 Hz). **HRMS** (ESI) *m/z*: [M+Na]+: Calcd for $C_{15}H_{21}FONa$: 259.1469, found: 259.1466.

2-fluoro-1-phenylcycloheptan-1-ol major isomer (2ba)

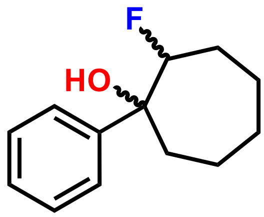

Synthesized according to cyclic fluorination method using (**1b**) (191.9 mg, 1.0 mmol, 1.0 eq) with Selectfluor™ (1.40 g, 4.0 mmol, 4.0 eq) and Toluene (0.21 mL, 2.0 mmol, 2.0 eq). Purified via prep TLC on neutral aluminum oxide 10% ethyl ether in hexanes.
Colorless Oil; **Mass:** 20.8 mg **Yield:** 10.0% **R*f*:** 0.32 (20% Ethyl Ether/Hexanes). **^{1}H NMR (600MHz, C_6D_6):** δ 7.36 (d, J= 7.8 Hz, 2H), 7.21 (t, J= 7.6 Hz, 2H), 7.11 (t, J= 7.3 Hz, 1H), 4.62 (dd, J= 45.2, 10.9 Hz, 1H), 2.31 (br s, 1H), 2.16 (q, J= 12.1 Hz, 1H), 1.89-1.81 (m, 1H), 1.78-1.70 (m, 2H), 1.52 (t, J= 12.7 Hz, 2H), 1.41-1.35 (m, 1H), 1.29-1.11 (m, 3H). **^{13}C{^{1}H} NMR (150MHz, C_6D_6):** δ 147.8, 128.1, 126.6, 124.7, 99.0, 97.9, 76.5, 76.4, 38.9 (d), 28.1, 28.0, 26.4, 21.3, 21.2, 19.8. **^{19}F NMR (565 MHz, C_6D_6):** δ -176.4 (dd, J= 46.3, 18.7 Hz). **HRMS** (ESI) *m/z*: [M+NH$_4$]+: Calcd for $C_{13}H_{21}FON$: 226.1602, found: 226.1600.

2-fluoro-1-phenylcycloheptan-1-ol minor isomer (2bb)

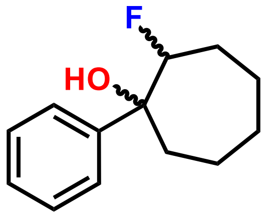

Synthesized according to cyclic fluorination method using (**1b**) (191.9 mg, 1.0 mmol, 1.0 eq) with Selectfluor™ (1.40 g, 4.0 mmol, 4.0 eq) and Toluene (0.21 mL, 2.0 mmol, 2.0 eq). Purified via prep TLC on neutral aluminum oxide 10% ethyl ether in hexanes.
Colorless Oil; **Mass:** 19.2 mg **Yield:** 9.2% **R*f*:** 0.23 (20% Ethyl Ether/Hexanes). **^{1}H NMR (600MHz, C_6D_6):** δ 7.52 (d, J= 7.8 Hz, 2H), 7.21 (t, J= 7.7 Hz, 2H), 7.12 (t, J= 7.3 Hz, 1H), 4.53 (dd, J= 46.6, 6.9 Hz, 1H), 2.18 (t, J= 13.2 Hz, 1H), 1.94-1.89 (m, .5H), 1.87-1.80 (m, 1.5H), 1.76-1.69 (m, 1H), 1.62-1.50 (m, 3H), 1.44-1.31 (m, 4H). **^{13}C{^{1}H} NMR (150MHz, C_6D_6):** δ 146.8, 127.8, 126.9, 125.9 (d), 98.5, 97.3, 76.9, 76.7, 36.6, 29.3, 29.2, 27.2, 21.6, 21.5, 21.1. **^{19}F NMR (565 MHz, C_6D_6):** δ -182.0 (m). **HRMS** (ESI) *m/z*: [M+Na]+: Calcd for $C_{13}H_{17}FONa$: 231.1156, found: 231.1155.

1-(3-ethylphenyl)-2-fluorocycloheptan-1-ol major isomer (2c)

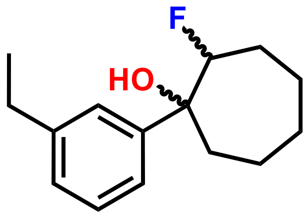

Synthesized according to cyclic fluorination method using (**1c**) (59.6 mg, 0.27 mmol, 1.0 eq) with Selectfluor™ (383 mg, 1.0 mmol, 4.0 eq) and Toluene (49.8 mg, 0.54 mmol, 2.0 eq). Purified via prep TLC on neutral aluminum oxide 10% ethyl ether in hexanes.
Colorless Oil; **Mass:** 20.8 mg **Yield:** 32.2% **R*f*:** 0.38 (20% Ethyl Ether/Hexanes). **^{1}H NMR (600MHz, C_6D_6):** δ 7.38 (s, 1H), 7.21 (m, 2H), 7.01 (m, 1H), 4.70 (dd, J= 44.6, 10.8, 2.50 Hz, 1H), 2.52 (q, J= 7.6 Hz, 2H), 2.28 (br s, 1H), 2.19 (q, J= 13.2 Hz, 1H), 1.92-1.72 (m, 3H), 1.60-1.50 (m, 2H), 1.43-1.35 (m, 1H), 1.30-1.21 (m, 2H), 1.18-1.12 (m, 4H). **^{13}C{^{1}H} NMR (150MHz, C_6D_6):** δ 148.0, 144.0, 128.2, 126.2, 124.3, 122.1, 99.1, 97.9, 76.5, 76.4, 39.0 (d), 29.1, 28.1, 28.0, 26.4, 21.3 (d), 19.8, 15.6. **^{19}F NMR (565 MHz, C_6D_6):** δ -176.3 (dd, J= 45.2, 20.7 Hz). **HRMS** (ESI) *m/z*: [M+Na]+: Calcd for $C_{15}H_{21}FONa$: 259.1469, found: 259.1467.

2-fluoro-1-(4-methoxyphenyl)cycloheptan-1-ol major isomer (2da)

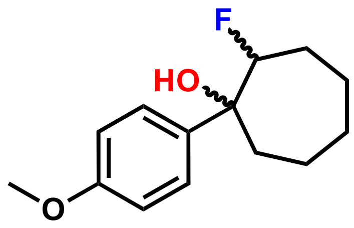

Synthesized according to cyclic fluorination method using (**1d**) (65.5 mg, 0.3 mmol, 1.0 eq) with Selectfluor™ (414.6 mg, 1.2 mmol, 4.0 eq) and Toluene (0.06 mL, 0.6 mmol, 2.0 eq). Purified via prep TLC on neutral aluminum oxide 10% ethyl ether in hexanes.
White solid, **Melting Point**: 63.0°C; **Mass:** 20.7 mg **Yield:** 29.2% **R*f*:** 0.32 (20% Ethyl Ether/Hexanes). **^{1}H NMR (600MHz, C_6D_6):** δ 7.31 (d, J= 8.7 Hz, 2H), 6.84 (d, J= 8.7 Hz, 2H), 4.63 (ddd, J= 44.8, 10.8, 2.4 Hz, 1H), 3.36 (s, 3H), 2.29 (d, J= 2.9 Hz, 1H), 2.19 (q, J= 12.5 Hz, 1H), 1.91-1.72 (m, 3H), 1.59-1.52 (m, 2H), 1.44-1.37 (m, 1H), 1.32-1.14 (m, 3H). **^{13}C{^{1}H} NMR (150MHz, C_6D_6):** δ 158.7, 139.9, 128.2, 125.9, 113.6, 99.0, 97.9, 76.2, 76.0 54.5, 39.2, 39.1, 28.1, 27.9, 26.5, 21.4, 21.3, 19.8.**^{19}F NMR (565 MHz, C_6D_6):** δ -176.4 (dd, J= 44.9, 21.5 Hz). **HRMS** (ESI) *m/z*: [M+Na]+: Calcd for $C_{14}H_{19}FO_2Na$: 261.1261, found: 261.1258.

2-fluoro-1-(4-methoxyphenyl)cycloheptan-1-ol minor isomer (2db)

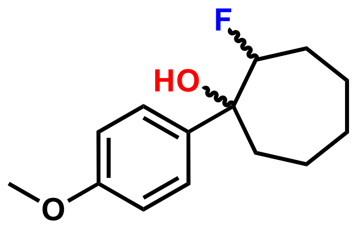

Synthesized according to cyclic fluorination method using (**1d**) (65.5 mg, 0.3 mmol, 1.0 eq) with Selectfluor™ (414.6 mg, 1.2 mmol, 4.0 eq) and Toluene (0.06 mL, 0.6 mmol, 2.0 eq). Purified via prep TLC on neutral aluminum oxide 10% ethyl ether in hexanes.
Colorless oil; **Mass:** 2.8 mg **Yield:** 4.0% **R*f*:** 0.09 (20% Ethyl Ether/Hexanes). **^{1}H NMR (600MHz, C_6D_6):** δ 7.46 (d, J= 8.7 Hz, 2H), 6.84 (d, J= 8.8 Hz, 2H), 4.57 (dd, J= 46.3, 6.5 Hz, 1H), 3.35 (s, 3H), 2.22 (t, J= 13.2 Hz, 1H), 1.98-1.83 (m, 2H), 1.78-1.72 (m, 1H), 1.65-1.52 (m, 3H), 1.47-1.33 (m, 3H), 1.29 (s, 1H). **^{13}C{^{1}H} NMR (150MHz, C_6D_6):** δ 158.9, 138.9, 128.2, 127.1 (d), 113.3, 98.7, 97.5, 54.4, 36.7 (d), 29.3,

29.2, 27.2, 21.5 (d), 21.0. **^{19}F NMR (565 MHz, C_6D_6):** δ -181.9 (m). **HRMS** (ESI) *m/z*: [M+Na]+: Calcd for $C_{14}H_{19}FO_2Na$: 261.1261, found: 261.1258.

2-fluoro-1-(4-(trifluoromethyl)phenyl)cycloheptan-1-ol major isomer (2e)

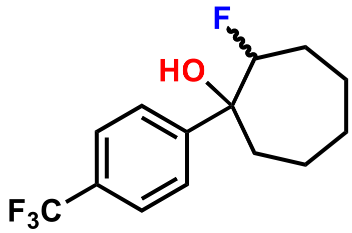

Synthesized according to cyclic fluorination method using (**1e**) (915.8 mg, 3.39 mmol, 1.0 eq) with CAN (2.0 g, 3.72 mmol, 1.1 eq), Selectfluor™ (4.79 g, 13.6 mmol, 4.0 eq), and Toluene (0.72 mL, 6.76 mmol, 2.0 eq). Purified via prep TLC on neutral aluminum oxide 10% ethyl ether in hexanes.
White Solid, **Melting Point**: 51.4°C; **Mass:** 115.2mg **Yield:** 12.3% **R*f*:** 0.28 (20% Ethyl Ether/Hexanes). **^{1}H NMR (600MHz, C_6D_6):** δ 7.42 (d, J= 8.2 Hz, 2H), 7.17 (d, 2H [overlaps with residual benzene signal]), 4.43 (ddd, J= 45.0, 10.8, 2.8 Hz, 1H), 2.10-2.04 (m, 2H), 1.81- 1.65 (m, 2H), 1.59-1.54 (m, 1H), 1.52-1.46 (m, 1H), 1.40-1.28 (m, 2H), 1.25-1.07 (m, 3H). **^{13}C{^{1}H} NMR (150MHz, C_6D_6):** δ 151.7, 125.1, 125.0 (d), 98.7, 97.5, 76.5, 76.3, 38.4 (d), 27.9, 27.8, 26.1, 21.0 (d), 19.6. **^{19}F NMR (565 MHz, C_6D_6):** δ -62.0 (s), -176.4 (dd, J= 45.3, 21.1 Hz). GC-MS confirms mass of 276. High Resolution Mass spectrometry analysis of this compound using ESI was unable to identify a molecular ion species. Analysis by GC/MS using EI allowed the following data to be obtained: a single large peak at 10.86 min with a parent ion of 276 closely matching the expected GC/MS m/z value for $C_{14}H_{16}F_4O$ of 276.11.

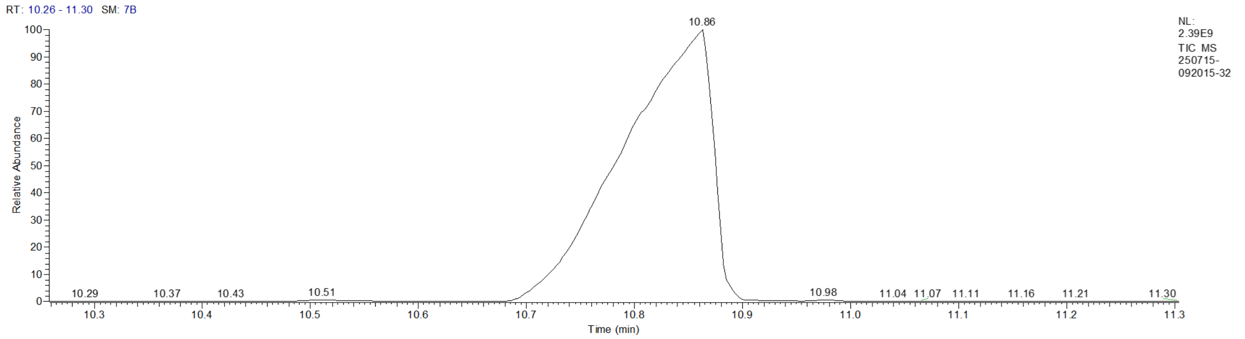

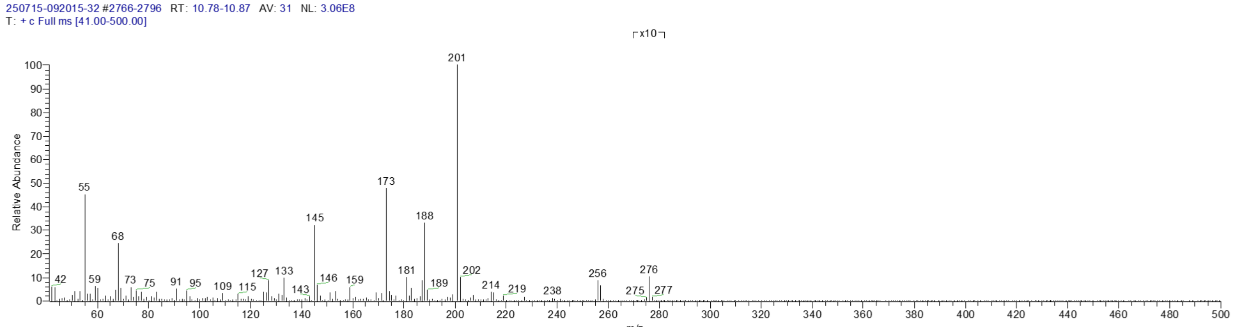

1-(4-ethylphenyl)-2-fluorocyclooctan-1-ol major isomer (4a)

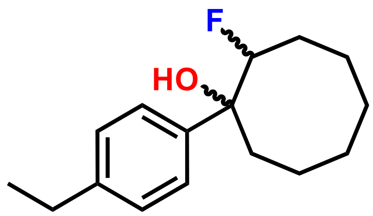

Synthesized according to cyclic fluorination method using (**3a**) (647.0 mg, 2.8 mmol, 1.0 eq) with Selectfluor™ (3.95 g, 11.2 mmol, 4.0 eq) and Toluene (0.58 mL, 5.58 mmol, 2.0 eq). Purified via prep TLC on neutral aluminum oxide 10% ethyl ether in hexanes.

Colorless Oil; **Mass:** 72.3 mg **Yield:** 10.3% **R*f*:** 0.37 (20% Ethyl Ether/Hexanes). **^{1}H NMR (600MHz, C_6D_6):** δ 7.45 (d, J= 8.2 Hz, 2H), 7.11 (d, J= 8.2 Hz, 2H), 5.19 (dd, J= 46.1, 9.2 Hz, 1H), 2.50 (q, J= 7.6 Hz, 2H), 2.45-2.40 (m, 1H), 2.06 (t, J= 8.8 Hz, 1H), 1.84 (ddt, J= 34.1, 15.0, 4.9 Hz, 1H), 1.74-1.69 (m, 1H), 1.56-1.52 (m, 1H), 1.46-1.30 (m, 4H), 1.24-1.18 (m, 1H), 1.14-1.08 (m, 4H), 1.03-0.97 (m, 1H). **^{13}C{^{1}H} NMR (150MHz, C_6D_6):** δ 143.2, 142.5, 127.6, 125.5, 97.1, 96.0, 77.2, 77.1, 36.5 (d), 28.4, 27.5, 27.4, 26.4, 24.0, 21.0, 15.5. **^{19}F NMR (565 MHz, C_6D_6):** δ -170.9 (t, J= 39.4 Hz). **HRMS** (ESI) *m/z*: [M+Na]+: Calcd for $C_{16}H_{23}FONa$: 273.1625, found: 273.1622.

2-fluoro-1-phenylcyclooctan-1-ol major isomer (4b)

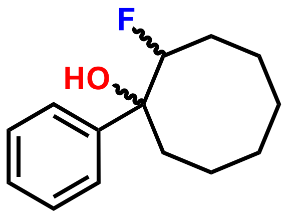

Synthesized according to cyclic fluorination method using (3b) (173.1 mg, 0.85 mmol, 1.0 eq) with Selectfluor™ (1.20 g, 3.4 mmol, 4.0 eq) and Toluene (156.9 mg, 1.7 mmol, 2.0 eq). Purified via prep TLC on neutral aluminum oxide 10% ethyl ether in hexanes.

Colorless Oil; **Mass:** 28.0 mg **Yield:** 14.8% **R*f*:** 0.29 (20% Ethyl Ether/Hexanes). **^{1}H NMR (600MHz, C_6D_6):** δ 7.46 (d, J= 7.8 Hz, 2H), 7.22 (t, J= 7.6 Hz, 2H), 7.20 (t, J= 7.3 Hz, 1H), 5.14 (dd, J= 45.6, 9.2 Hz, 1H), 2.45-2.39 (m, 2H), 2.02 (t, J= 13.6 Hz, 1H), 1.82 (ddt, J= 34.4, 15.2, 4.9 Hz, 1H), 1.67-1.65 (m, 1H), 1.53 (m, 1H), 1.44-1.30 (m, 4H), 1.20-1.07 (m, 2H), 1.01-0.95 (m, 1H). **^{13}C{^{1}H} NMR (150MHz, C_6D_6):** δ 145.9, 128.1, 126.8, 125.4, 96.0, 77.3, 77.2, 36.5, 36.4, 29.4, 29.2, 27.4, 27.3, 26.4, 23.9, 20.9. **^{19}F NMR (565 MHz, C_6D_6):** δ -171.0 (t, J= 38.2 Hz). **HRMS** (ESI) *m/z*: [M+Na]+: Calcd for $C_{14}H_{19}FONa$: 245.1312, found: 245.1310.

1-(3-ethylphenyl)-2-fluorocyclooctan-1-ol major isomer (4c)

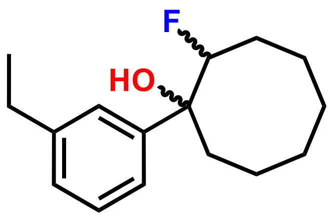

Synthesized according to cyclic fluorination method using (**3c**) (122.1 mg, 0.53 mmol, 1.0 eq) with Selectfluor™ (740.3 mg, 2.1 mmol, 4.0 eq) and Toluene (97.7 mg, 1.06 mmol, 2.0 eq). Purified via prep TLC on neutral aluminum oxide 10% ethyl ether in hexanes.
Colorless Oil; **Mass:** 7.3 mg **Yield:** 5.5% **R*f*:** 0.32 (20% Ethyl Ether/Hexanes). **^{1}H NMR (600MHz, C_6D_6):** δ 7.49 (s, 1H), 7.30 (d, J= 7.8 Hz, 1H), 7.22 (t, J= 7.6 Hz, 1H), 7.02 (d, J= 7.4 Hz, 1H), 5.21 (dd, J= 45.1, 9.2 Hz, 1H), 2.53 (q, J= 7.6 Hz, 2H), 2.48-2.42 (m, 1H), 2.39 (d, J= 5.0 Hz, 1H), 2.06 (t, J= 13.6 Hz, 1H), 1.85 (ddt, J= 34.2, 15.1, 4.8 Hz, 1H), 1.74-1.70 (m, 1H), 1.55-1.53 (m, 1H), 1.47-1.31 (m, 4H), 1.27-1.21 (m, 1H), 1.15-1.08 (m, 4H), 1.06-0.99 (m, 1H). **^{13}C{^{1}H} NMR (150MHz, C_6D_6):** δ 146.1, 144.0, 128.1, 126.4, 125.0, 122.8, 97.2, 96.1, 77.4, 77.2, 36.5 (d), 29.4, 29.3, 29.1, 27.4, 27.3, 26.4, 24.0, 21.0, 15.6. **^{19}F NMR (565 MHz, C_6D_6):** δ -170.9 (t, J= 39.3 Hz). **HRMS** (ESI) *m/z*: [M+Na]+: Calcd for $C_{16}H_{23}FONa$: 273.1625, found: 273.1621.

2-fluoro-1-(4-methoxyphenyl)cyclooctan-1-ol major isomer (4d)

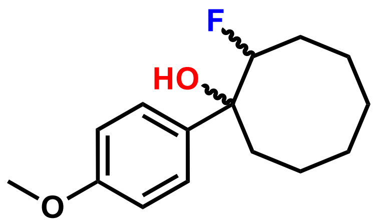

Synthesized according to cyclic fluorination method using (**3d**) (73.6 mg, 0.3 mmol, 1.0 eq) and Selectfluor™ (419.5 mg, 1.2 mmol, 4.0 eq) and Toluene (0.06 mL, 0.6 mmol, 2.0 eq). Purified via prep TLC on neutral aluminum oxide 10% ethyl ether in hexanes.
White solid, **Melting Point**: 73.3°C; **Mass:** 39.2 mg **Yield:** 49.5% **R*f*:** 0.18 (20% Ethyl Ether/Hexanes). **^{1}H NMR (600MHz, C_6D_6):** δ 7.40 (d, J= 7.6 Hz, 2H), 6.58 (d, J= 7.9 Hz, 2H), 5.16 (dd, J= 45.9, 9.2 Hz, 1H), 3.46 (s, 3H), 2.49-2.42 (m, 1H), 2.07 (t, J= 13.5 Hz, 1H), 1.85 (ddt, J= 34.4, 15.1, 4.6 Hz, 1H), 1.72-1.70 (m, 1H), 1.57-1.55 (m, 1H), 1.48-1.33 (m, 4H), 1.27-1.21 (m, 1H), 1.14-1.10 (m, 1H), 1.04-0.97 (m, 1H). **^{13}C{^{1}H} NMR (150MHz, C_6D_6):** δ 158.8, 137.8, 128.2, 126.7, 113.6, 97.1, 95.9, 77.1, 76.9, 54.5, 36.6, 36.5, 29.5, 29.4, 27.5 (d), 26.4, 24.0, 21.1. **^{19}F NMR (565 MHz, C_6D_6):** δ -170.9 (t, J= 39.3 Hz). **HRMS** (ESI) *m/z*: [M+Na]+: Calcd for $C_{15}H_{21}FO_2Na$: 275.1418, found: 275.1413.

1-(4-ethylphenyl)-2-fluorocyclohexan-1-ol major isomer (6a)

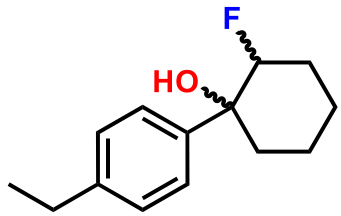

Synthesized according to cyclic fluorination method using (**5a**) (61.5 mg, 0.30 mmol, 1.0 eq) with CAN (181.6 mg, 0.33 mmol, 1.1 eq), Selectfluor™ (418.7 mg, 1.2 mmol, 4.0 eq), and Toluene (0.06 mL, 0.6 mmol, 2.0 eq). Purified via prep TLC on neutral aluminum oxide 10% ethyl ether in hexanes.
Colorless Oil; **Mass:** 26.2 mg **Yield:** 39.2% **R*f*:** 0.32 (20% Ethyl Ether/Hexanes). **^{1}H NMR (600MHz, C_6D_6):** δ 7.35 (d, J= 8.2 Hz, 2H), 7.09 (d, J= 8.1 Hz, 2H), 4.64 (ddd, J= 47.7, 11.8, 4.7 Hz, 1H), 2.49 (q, J= 7.6 Hz, 2H), 2.12-2.04 (br m, 1H), 2.02-1.93 (m, 1H), 1.87-1.82 (m, 1H), 1.81-1.76 (m, 1H), 1.74-1.66 (qt, J= 13.2, 3.8 Hz, 1H), 1.52 (br d, J= 13.5 Hz, 1H), 1.29 (br t, J= 13.7 Hz, 1H), 1.18 (br d, J= 12.9 Hz, 1H), 1.12 (t, J= 7.6 Hz, 3H), 0.96 (br q, J= 13.5 Hz, 1H). **^{13}C{^{1}H} NMR (150MHz, C_6D_6):** δ 143.3, 142.6, 127.7, 125.1, 95.1, 93.9, 74.6, 74.5, 39.1 (d), 28.4, 27.6, 27.5, 23.6, 23.5, 20.7, 15.5. **^{19}F NMR (565 MHz, C_6D_6):** δ -185.2 (d, J= 46.1 Hz). **HRMS** (ESI) *m/z*: [M+Na]+: Calcd for $C_{14}H_{19}FONa$: 245.1312, found: 245.1311.

2-fluoro-1-phenylcyclohexan-1-ol major isomer (6b)[5]

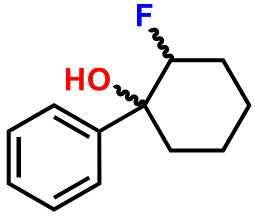

Synthesized according to cyclic fluorination method using (**5b**) (52.8 mg, 0.3 mmol, 1.0 eq) with CAN (181.0 mg, 0.33 mmol, 1.1 eq), Selectfluor™ (423.2 mg, 1.2 mmol, 4.0 eq), and Toluene (0.06 mL, 0.6 mmol, 0.2 eq). Purified via prep TLC on neutral aluminum oxide 10% ethyl ether in hexanes.
White Solid, **Melting Point**: 73.4°C; **Mass:** 14.0 mg **Yield:** 24.1% **R*f*:** 0.32 (20% Ethyl Ether/Hexanes). **^{1}H NMR (600MHz, C_6D_6):** δ 7.36 (d, J= 7.6 Hz, 2H), 7.21 (t, J= 7.7 Hz, 2H), 7.11 (t, J= 7.3 Hz, 1H), 4.60 (ddd, J= 47.2, 11.18, 5.04 Hz, 1H), 2.00-1.90 (m, 2H), 1.82-1.74 (m, 1H), 1.71-1.63 (qt, J= 13.1, 3.8 Hz, 1H), 1.50 (br m, 1H), 1.23 (br t, J= 14.1 Hz, 1H), 1.15 (br m, 1H), 0.94 (q, J= 13.4 Hz, 1H). **^{13}C{^{1}H} NMR (150MHz, C_6D_6):** δ 145.9, 128.1, 126.8, 125.0, 95.0, 93.8, 74.7, 74.5, 39.0 (d), 27.6, 27.5, 23.6, 23.5, 20.6. **^{19}F NMR (565 MHz, C_6D_6):** δ -185.3 (d, J= 46.1 Hz). Spectra taken in $CDCl_3$ match reported spectra.[5] **HRMS** (ESI) *m/z*: [M+Na]+: Calcd for $C_{12}H_{15}FONa$ 217.0999, found: 217.0998.

1-(3-ethylphenyl)-2-fluorocyclohexan-1-ol major isomer (6c)

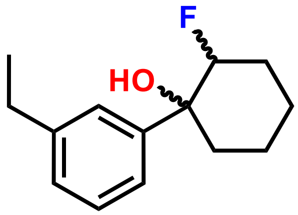

Synthesized according to cyclic fluorination method using (**5c**) (61.8 mg, 0.3 mmol, 1.0 eq) with CAN (183.4 mg, 0.33 mmol, 1.1 eq), Selectfluor™ (425.0 mg, 1.2 mmol, 4.0 eq), and Toluene (0.06 mL, 0.6 mmol, 2.0 eq). Purified via prep TLC on neutral aluminum oxide 10% ethyl ether in hexanes.
Colorless Oil; **Mass:** 18.3 mg **Yield:** 27.2% **R*f*:** 0.35 (20% Ethyl Ether/Hexanes). **^{1}H NMR (600MHz, C_6D_6):** δ 7.36 (s, 1H), 7.23-7.19 (m, 2H), 7.01 (d, J= 6.9 Hz, 1H), 4.67 (ddd, J= 47.2, 11.2, 5.0 Hz, 1H), 2.52 (q, J= 7.6 Hz, 2H), 2.08-1.93 (m, 2H), 1.87-1.77 (m, 2H), 1.70 (qt, J= 13.2, 3.8 Hz, 1H), 1.52 (br d, J= 13.2 Hz, 1H), 1.29 (br t, J= 13.7 Hz, 1H), 1.19-1.12 (m, 4H), 0.96 (br q, J= 13.4 Hz, 1H). **^{13}C{^{1}H} NMR (150MHz, C_6D_6):** δ 146.0, 144.0, 128.2, 126.4, 124.6, 122.5, 95.0, 93.9, 74.7, 74.6, 39.1 (d), 29.1, 27.6,

27.5, 23.6, 23.5, 20.7, 15.6. **^{19}F NMR (565 MHz, C_6D_6):** δ -185.1 (d, J= 46.7 Hz). **HRMS** (ESI) *m/z*: [M+Na]+: Calcd for $C_{14}H_{19}FONa$: 245.1312, found: 245.1310.

2-fluoro-1-(4-methoxyphenyl)cyclohexan-1-ol major isomer (6d)

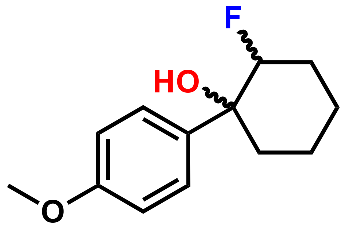

Synthesized according to cyclic fluorination method using (**5d**) (13.4 mg, 0.06 mmol, 1.0 eq) with CAN (38.6 mg, 0.07 mmol, 1.1 eq), and Selectfluor™ (90.7 mg, 0.26 mmol, 4.0 eq). Purified via prep TLC on Silica 10% ethyl acetate in hexanes.
White Solid, **Melting Point**: 105.0°C**; Mass:** 23.6 mg **Yield:** 25.8% **R*f*:** 0.31 (20% Ethyl Ether/Hexanes). **^{1}H NMR (600MHz, C_6D_6):** δ 7.31 (d, J= 8.7 Hz, 2H), 6.84 (d, J= 8.9 Hz, 2H), 4.61 (ddd, J= 47.1, 11.1, 4.9, Hz, 1H), 3.35 (s, 3H), 2.04-1.94 (m, 2H), 1.87-1.77 (m, 2H), 1.71 (qt, J= 21.8, 3.8 Hz, 1H), 1.56-1.52 (m, 1H), 1.27 (t, J= 13.7 Hz, 1H), 1.21-1.18 (m, 1H), 0.97 (q, J= 13.4 Hz, 1H). **^{13}C{^{1}H} NMR (150MHz, C_6D_6):** δ 158.9, 138.0, 128.2, 126.3, 113.6, 95.1, 93.9, 74.3, 74.2, 54.5, 39.2, 39.1, 27.7, 27.6, 23.6, 23.5, 20.8. **^{19}F NMR (565 MHz, C_6D_6):** δ -185.3 (d, J= 46.9 Hz). **HRMS** (ESI) *m/z*: [M+Na]+: Calcd for $C_{13}H_{17}FO_2Na$: 247.1105, found: 247.1102.

1-(4-ethylphenyl)-2-fluorocyclopentan-1-ol major isomer (8a)

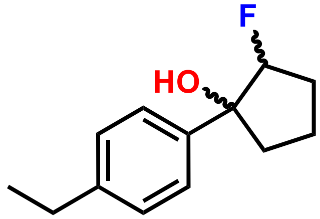

Synthesized according to cyclic fluorination method using (**7a**) (57.6 mg, 0.3 mmol, 1.0 eq) with CAN (181.9 mg, 0.33 mmol, 1.1 eq), Selectfluor™ (422.4 mg, 1.2 mmol, 4.0 eq), and Toluene (0.06 mL, 0.6 mmol, 2.0 eq). Filtered through plug of neutral aluminum oxide prior to purification. Purified via prep TLC on neutral aluminum oxide 10% ethyl ether in hexanes.
Colorless Oil; **Mass:** 17.3 mg **Yield:** 27.7% **R*f*:** 0.21 (20% Ethyl Ether/Hexanes). **^{1}H NMR (600MHz, C_6D_6):** δ 7.43 (d, J= 7.9 Hz, 2H), 7.08 (d, J= 7.9 Hz, 2H), 4.69 (dd, J= 52.2, 4.32 Hz, 1H), 2.48 (q, J= 7.6 Hz, 2H), 2.32-2.27 (m, 1H), 2.21-2.08 (m, 1H), 1.91-1.82 (m, 1H), 1.78-1.73 (m, 2H), 1.65 (br m, 1H), 1.11 (t, J= 7.6 Hz, 3H). **^{13}C{^{1}H} NMR (150MHz, C_6D_6):** δ 143.3, 139.4, 127.5, 126.9, 99.7, 98.5, 83.2, 83.0, 35.5, 30.8, 30.6, 28.5, 20.6, 15.5. **^{19}F NMR (565 MHz, C_6D_6):** δ -173.5 (m). **HRMS** (ESI) *m/z*: [M+Na]+: Calcd for $C_{13}H_{17}FONa$: 231.1156, found: 231.1154.

2-fluoro-1-phenylcyclopentan-1-ol major isomer (8b)

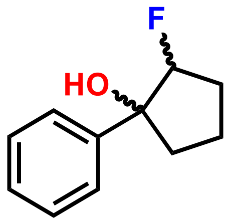

Synthesized according to cyclic fluorination method using (**7b**) (49.4 mg, 0.3 mmol, 1.0 eq) with CAN (181.9 mg, 0.33 mmol, 1.1 eq), Selectfluor™ (422.7 mg, 1.2 mmol, 4.0 eq), and Toluene (0.06 mL, 0.6 mmol, 2.0 eq). Filtered through plug of neutral aluminum oxide prior to purification. Purified via prep TLC on neutral aluminum oxide 10% ethyl ether in hexanes.
Colorless Oil; **Mass:** 8.7 mg **Yield:** 16.1% **R*f*:** 0.15 (20% Ethyl Ether/Hexanes). **^{1}H NMR (600MHz, C_6D_6):** δ 7.45 (d, J= 7.7 Hz, 2H), 7.20 (d, J= 7.3 Hz, 2H), 7.12 (t, J= 7.3 Hz, 1H), 4.66 (dd, J= 51.9, 4.4 Hz, 1H), 2.29-2.23 (m, 1H), 2.18-2.05 (m, 1H), 1.89-1.80 (m, 1H), 1.75-1.70 (m, 2H), 1.61-1.58 (m, 1H), 0.92 (br s, 1H). **^{13}C{^{1}H} NMR (150MHz, C_6D_6):** δ 142.0, 127.4, 126.8 (d), 99.6, 98.4, 83.2, 83.0, 35.5, 30.8, 30.6, 20.6. **^{19}F NMR (565 MHz, C_6D_6):** δ -173.7 (m). **HRMS** (ESI) *m/z*: [M+Na]+: Calcd for $C_{11}H_{13}FONa$ 203.0843, found: 203.0843.

1-(3-ethylphenyl)-2-fluorocyclopentan-1-ol major isomer (8c)

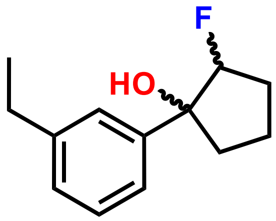

Synthesized according to cyclic fluorination method using (**7c**) (57.8 mg, 0.3 mmol, 1.0 eq) with CAN (181.0 mg, 0.33 mmol, 1.1 eq), Selectfluor™ (420.3 mg, 1.2 mmol, 4.0 eq), and Toluene (0.06 mL, 0.6 mmol, 2.0 eq). Purified via prep TLC on neutral aluminum oxide 10% ethyl ether in hexanes.
Colorless Oil; **Mass:** 16.7 mg **Yield:** 26.4% **R*f*:** 0.21 (20% Ethyl Ether/Hexanes). **^{1}H NMR (600MHz, C_6D_6):** δ 7.40 (s, 1H), 7.33 (d, J= 7.5 Hz, 1H), 7.20 (t, J= 7.6 Hz, 1H), 7.02 (d, J= 7.5 Hz, 1H), 4.71 (dd, J= 52.4, 4.6 Hz, 1H), 2.51 (q, J= 7.6 Hz, 2H), 2.34-2.28 (m, 1H), 2.21-2.08 (m, 1H), 1.91-1.82 (m, 1H), 1.78-1.73 (m, 2H), 1.67-1.64 (m, 1H), 1.13 (t, J= 7.6 Hz, 3H), 1.00-0.94 (br s, 1H). **^{13}C{^{1}H} NMR (150MHz, C_6D_6):** δ 143.8, 142.1, 128.1, 127.0, 126.4 (d), 124.3 (d), 99.7, 98.5, 83.3, 83.2, 35.5, 30.8, 30.7, 29.0, 20.6, 15.6. **^{19}F NMR (565 MHz, C_6D_6):** δ -173.5 (m). **HRMS** (ESI) *m/z*: [M+Na]+: Calcd for $C_{13}H_{17}FONa$: 231.1156, found: 231.1153.

2-fluoro-1-(4-methoxyphenyl)cyclopentan-1-ol major isomer (8d)

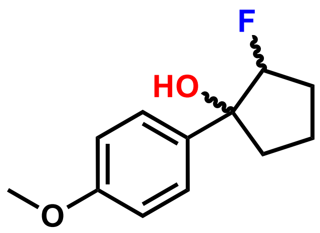

Synthesized according to cyclic fluorination method using (**7d**) (9.8 mg, 0.05 mmol, 1.0 eq) with CAN (30.7 mg, 0.06 mmol, 1.1 eq), and Selectfluor™ (73.0 mg, 0.20 mmol, 4.0 eq). Purified via prep TLC on Silica 15% ethyl acetate in hexanes.
Colorless oil; **Mass:** 2.94 mg **Yield:** 28.0% **R*f*:** 0.23 (15% Ethyl Ether/Hexanes). **^{1}H NMR (600MHz, $CDCl_3$):** δ 7.47 (d, J= 8.5 Hz, 2H), 6.92 (d, J= 8.8 Hz, 2H), 4.87 (dd, J= 52.0, 4.6 Hz, 1H), 3.82 (s, 3H), 2.45-2.28 (m, 2H), 2.03-1.96 (m, 4H). **^{13}C{^{1}H}DEPTQ135 NMR (150MHz, $CDCl_3$):** δ 159.13, 133.8, 127.89, 127.88, 113, 99.99.3 (d, J= 179.11), 55.3, 35.5, 30.6 (d, J= 22.04), 20.6. **^{19}F NMR (565 MHz, $CDCl_3$):** δ -173.5 **HRMS** (ESI) *m/z*: [M+Na]+: Calcd for $C_{12}H_{15}FO_2Na$: 233.0948, found: 233.0947.

Characterization of Methoxy Addition Products

Synthesized via standard procedure with 1:1 mixture of MeCN/MeOH as solvent.

Two diastereomers are synthesized during the reaction. Where possible, these diastereomers were separated and characterized. Experimental data and NMR spectra have been included for each isolated diastereomer and sub labeled "a" or "b" e.g. "2aa" and "2ab". In all other instances, data is reported for the major isomer.

1-(4-ethylphenyl)-2-fluoro-1-methoxycycloheptane major isomer (10aa)

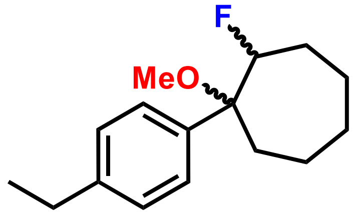

Synthesized according to cyclic fluorination method B using (**1a**) (66.7 mg, 0.3 mmol, 1.0 eq) Selectfluor™ (429.0 mg, 1.2 mmol, 4.0 eq) and Toluene (0.06 mL, 0.6 mmol, 2.0 eq). Purified via prep TLC on neutral aluminum oxide 10% ethyl ether in hexanes.
Colorless oil; **Mass:** 7.9 mg **Yield:** 10.3% **R*f*:** 0.75 (20% Ethyl Ether/Hexanes). **^{1}H NMR (600MHz, C_6D_6):** δ 7.43 (d, J= 7.8 Hz, 2H), 7.13 (d, J= 7.6 Hz, 2H), 4.50 (dd, J= 44.7, 10.5 Hz, 1H), 3.12 (s, 3H), 2.50 (q, J= 7.5 Hz, 2H), 2.37 (q, J= 11.7 Hz, 1H), 1.94-1.72 (m, 3H), 1.60-1.47 (m, 3H), 1.29-1.19 (m, 3H), 1.13 (t, J= 7.5 Hz, 3H). **^{13}C{^{1}H} NMR (150MHz, C_6D_6):** δ 142.6, 140.4, 127.4, 98.7, 97.5, 81.3, 81.2, 51.3, 51.2, 35.6, 35.5, 28.4, 28.3, 28.2, 26.1, 21.6, 21.5, 20.2, 15.5. **^{19}F NMR (565 MHz, C_6D_6):** δ -172.7 (dd, J= 45.02, 22.84 Hz). **HRMS** (ESI) *m/z*: [M+Na]+: Calcd for $C_{16}H_{23}FONa$: 273.1625, found: 273.1622.

1-(4-ethylphenyl)-2-fluoro-1-methoxycycloheptane minor isomer (10ab)

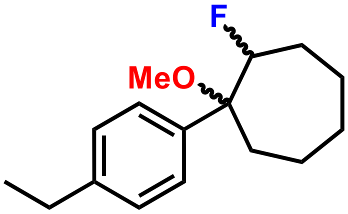

Synthesized according to cyclic fluorination method B using (**1a**) (66.7 mg, 0.3 mmol, 1.0 eq) Selectfluor™ (429.0 mg, 1.2 mmol, 4.0 eq) and Toluene (0.06 mL, 0.6 mmol, 2.0 eq). Purified via prep TLC on neutral aluminum oxide 10% ethyl ether in hexanes.
Colorless oil; **Mass:** 6.9 mg **Yield:** 9.0% **R*f*:** 0.86 (20% Ethyl Ether/Hexanes). **^{1}H NMR (600MHz, C_6D_6):** δ 7.42 (d, J= 7.5 Hz, 2H), 7.12 (d, J= 7.5 Hz, 2H), 4.72 (dd, J= 45.4, 6.2 Hz, 1H), 2.88 (s, 3H), 2.49 (q, J= 7.5 Hz, 2H), 2.17 (t, J= 13.0 Hz, 1H), 2.10-1.85 (m, 4H), 1.71-1.65 (m, 1H), 1.61-1.41 (m, 4H), 1.11 (s, J= 7.5 Hz, 3H). **^{13}C{^{1}H} NMR (150MHz, C_6D_6):** δ 142.8, 140.5, 127.3, 97.7, 96.5, 81.7, 81.5, 49.5, 29.4, 28.4, 28.3, 26.7, 19.9, 15.4. **^{19}F NMR (565 MHz, C_6D_6):** δ -185.0 (t, J= 45.2 Hz). **HRMS** (ESI) *m/z*: [M+Na]+: Calcd for $C_{16}H_{23}FONa$: 273.1625, found: 273.1623.

Characterization of Isolated Ring Opened Aldehydes

5-hydroxy-5-(4-methoxyphenyl)pentanal (11)[2]

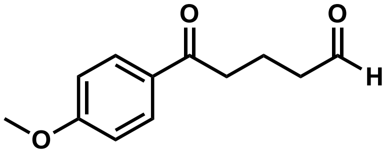

Synthesized according to cyclic fluorination method using (**7d**). Purified via prep TLC 15% ethyl acetate in hexanes.
^{1}H NMR (600MHz, $CDCl_3$): δ 9.81 (s, 0.88H), 7.94 (d, J= 8.96 Hz, 2H), 6.94 (d, J=8.96 Hz, 2H), 3.88 (s, 3H), 3.00 (t, J= 7.09 Hz, 2H), 2.59 (m, 2H), 2.07 (m, 2H). These results are in accordance with published literature.

6-hydroxy-6-(4-methoxyphenyl)hexanal (12)[2]

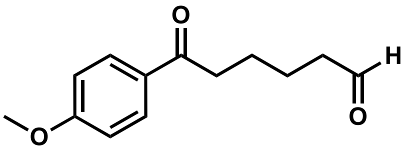

Synthesized according to cyclic fluorination method using (**5d**). Purified via prep TLC 10% ethyl acetate in hexanes.

^{1}H NMR (600MHz, $CDCl_3$): δ 9.79 (bt, J=1.60 Hz, 0.88H), 7.94 (d, J= 8.96 Hz, 2H), 6.94 (d, J= 8.96 Hz, 2H), 3.88 (s, 3H), 2.96 (t, J= 7.03 Hz, 2H), 2.51 (m, 2H), 1.80-1.70 (m, 5H). These results are in accordance with published literature.

8-(4-methoxyphenyl)-8-oxooctanal (13)[2]

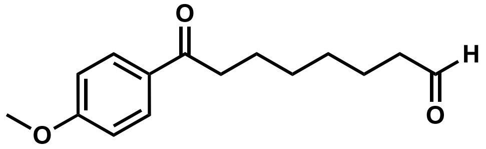

Synthesized according to cyclic fluorination method using (**3d**) including CAN (67.2 mg, 0.12 mmol). Purified via prep TLC 10% ethyl acetate in hexanes.
^{1}H NMR (600MHz, $CDCl_3$): δ 9.76 (t, J= 1.76 Hz, 0.83H), 7.94 (d, J= 8.89 Hz, 2H), 6.93 (d, J=8.89 Hz, 2H), 3.87 (s, 3H), 2.91 (t, J= 7.36 Hz, 2H), 2.44 (td, J= 7.34,1.76 Hz, 2H), 1.76-1.71 (m, 2H), 1.67-1.63 (m, 2H), 1.42-1.36 (m, 4H). These results are in accordance with published literature.

NMR spectra

Some Fluorine spectra contain small peaks in the -100- -130 range. We believe this represents trace amounts of aromatic ring fluorination side products consistent with established Selectfluor reactivity,[6] observable due to the high sensitivity of ^{19}F NMR.

^{1}H NMR (600MHz, C_6D_6) of 1a

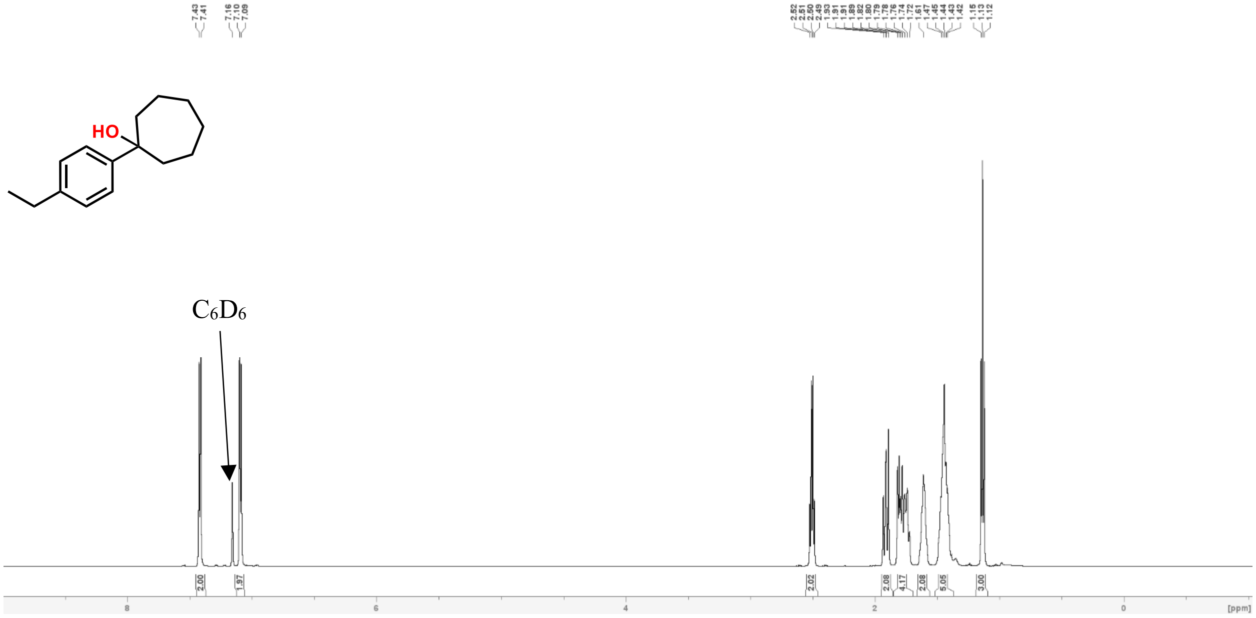

$^{13}C\{^{1}H\}$ NMR (150MHz, C_6D_6) of 1a

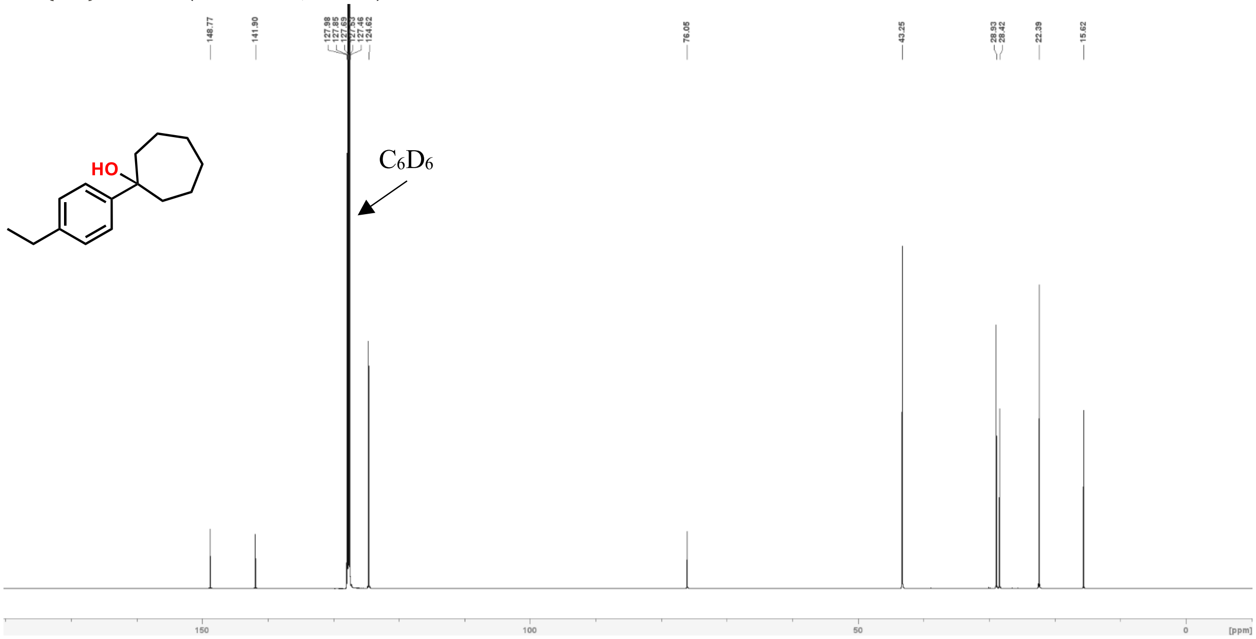

^{1}H NMR (600MHz, C_6D_6) of 1b

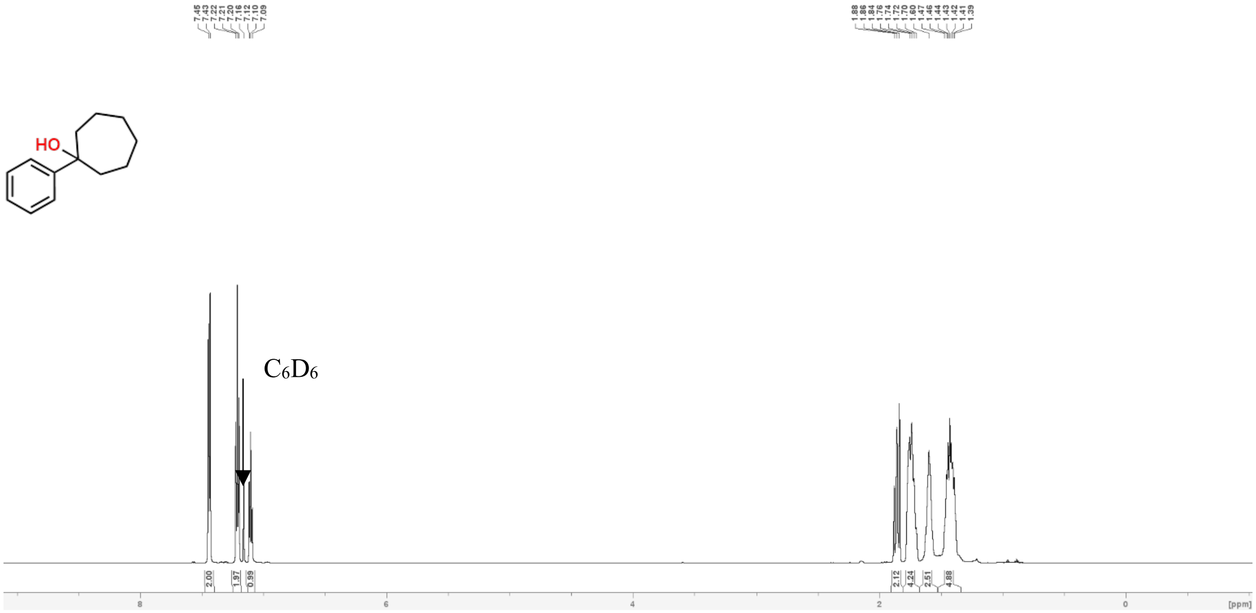

$^{13}C\{^1H\}$ NMR (150MHz, C_6D_6) of 1b

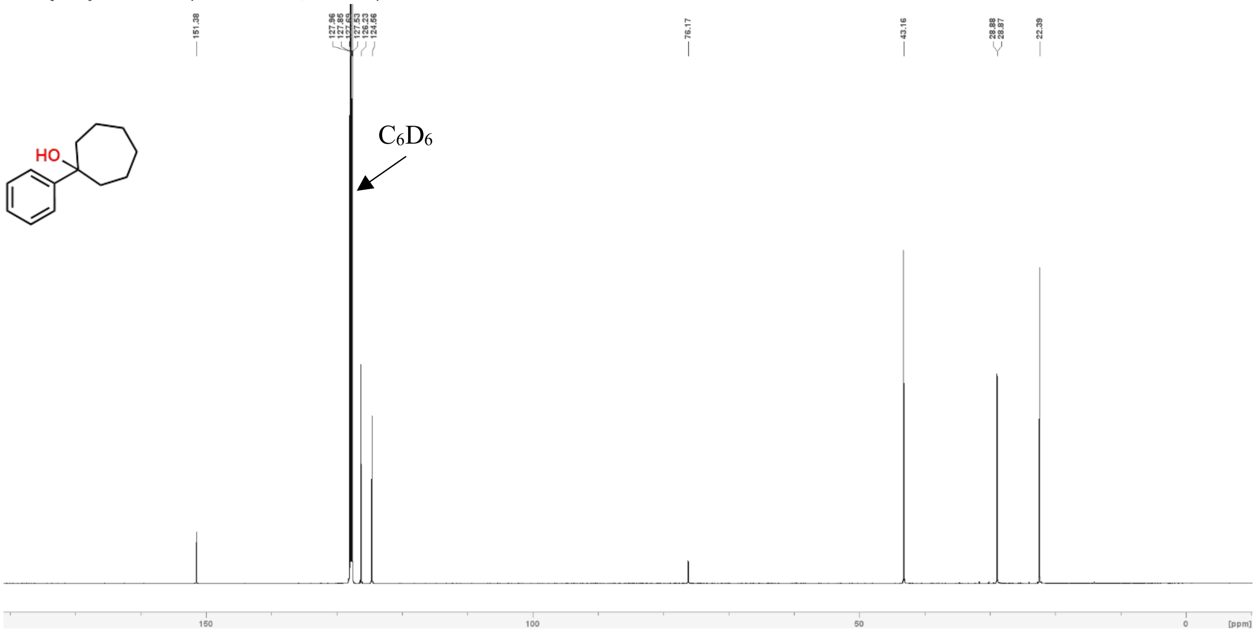

^1H NMR (600MHz, C_6D_6) of 1c

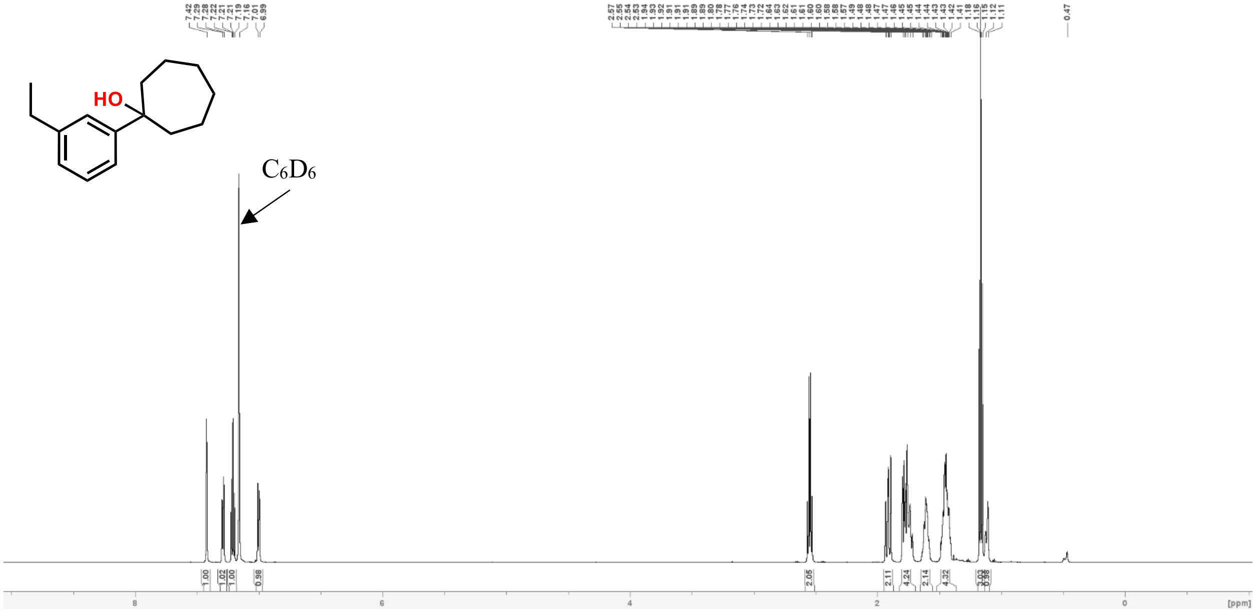

$^{13}C\{^{1}H\}$ NMR (150MHz, C_6D_6) of 1c

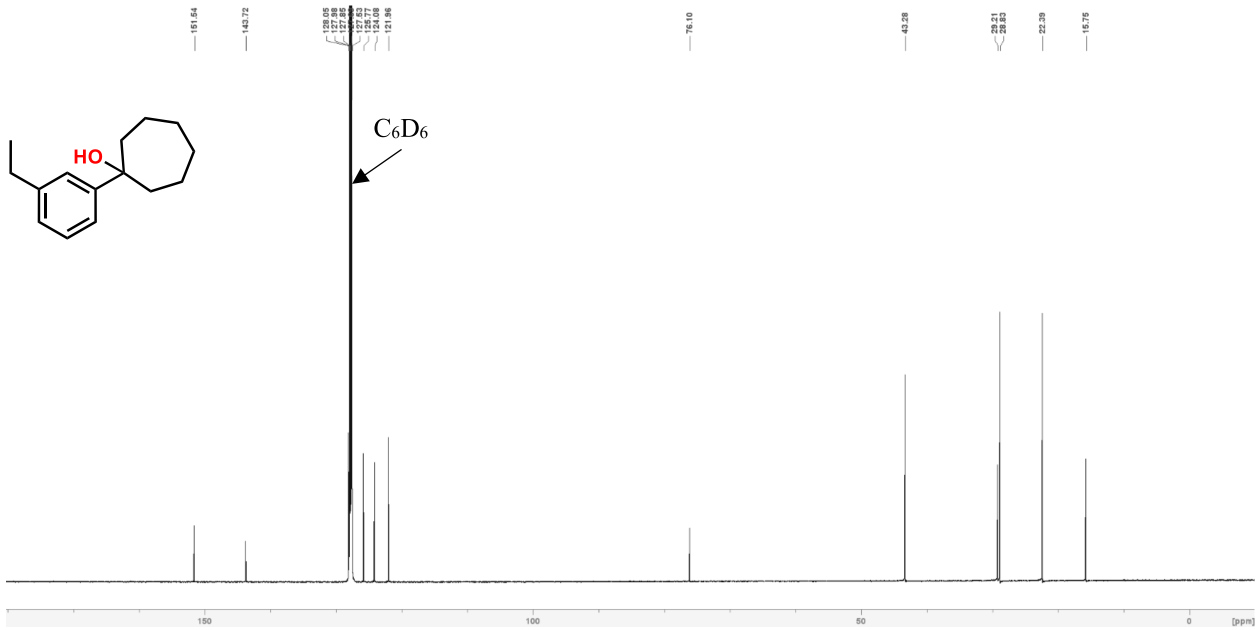

^{1}H NMR (600MHz, C_6D_6) of 1e

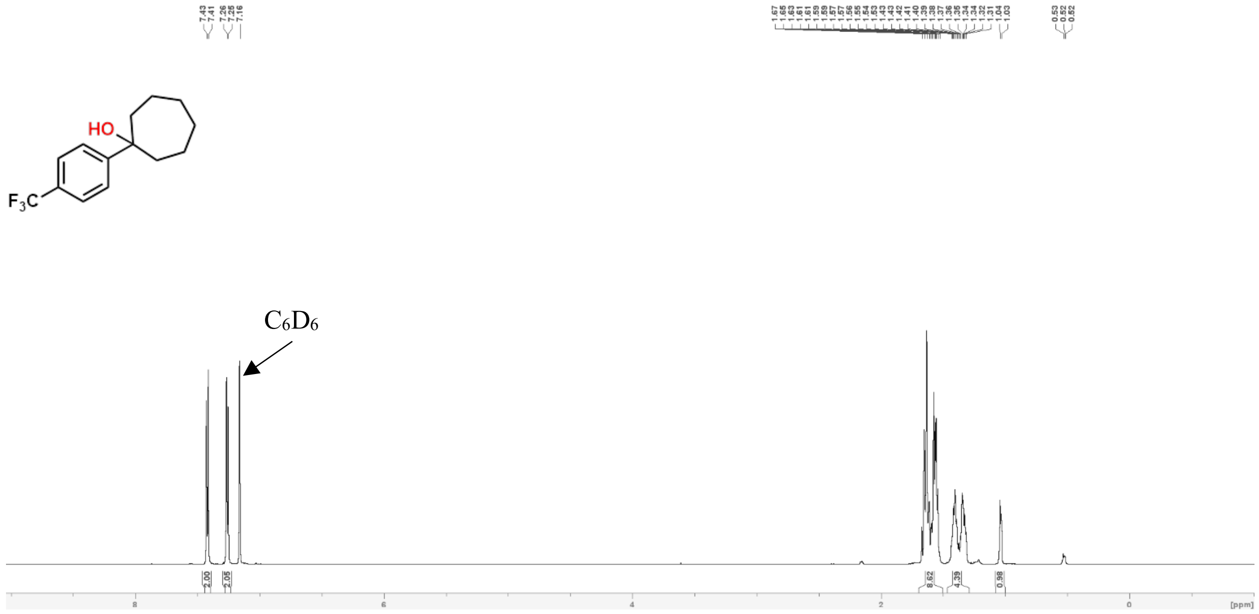

$^{13}C\{^1H\}$ NMR (150MHz, C_6D_6) of 1e

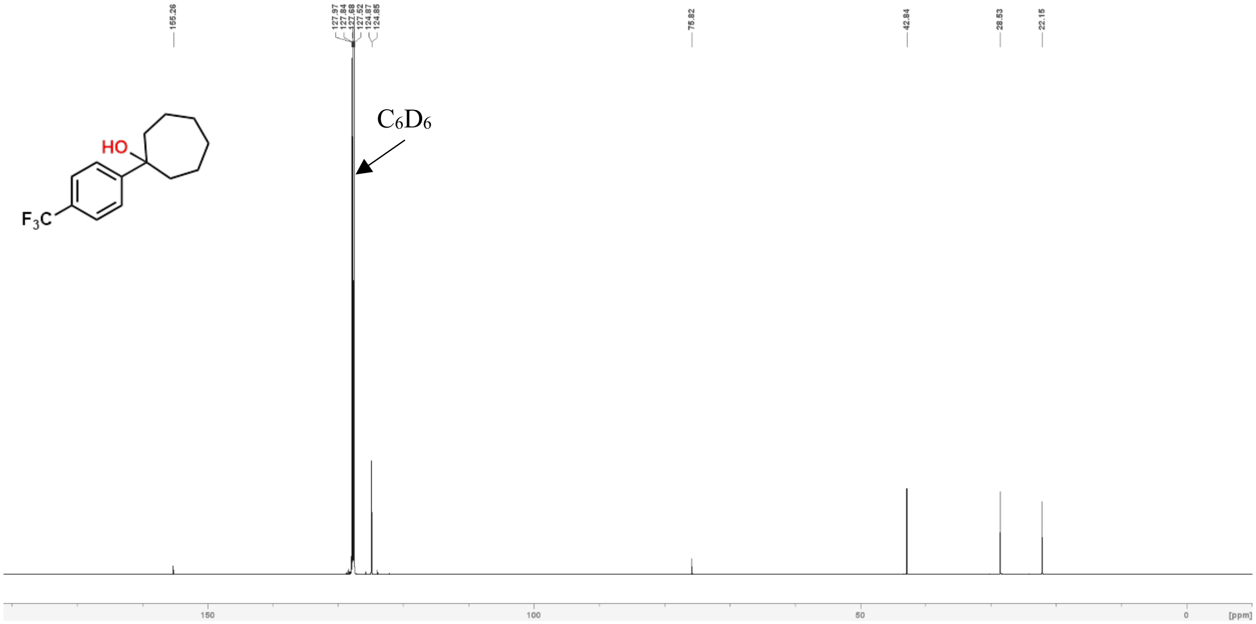

^9F NMR (565MHz, C_6D_6) of 1e

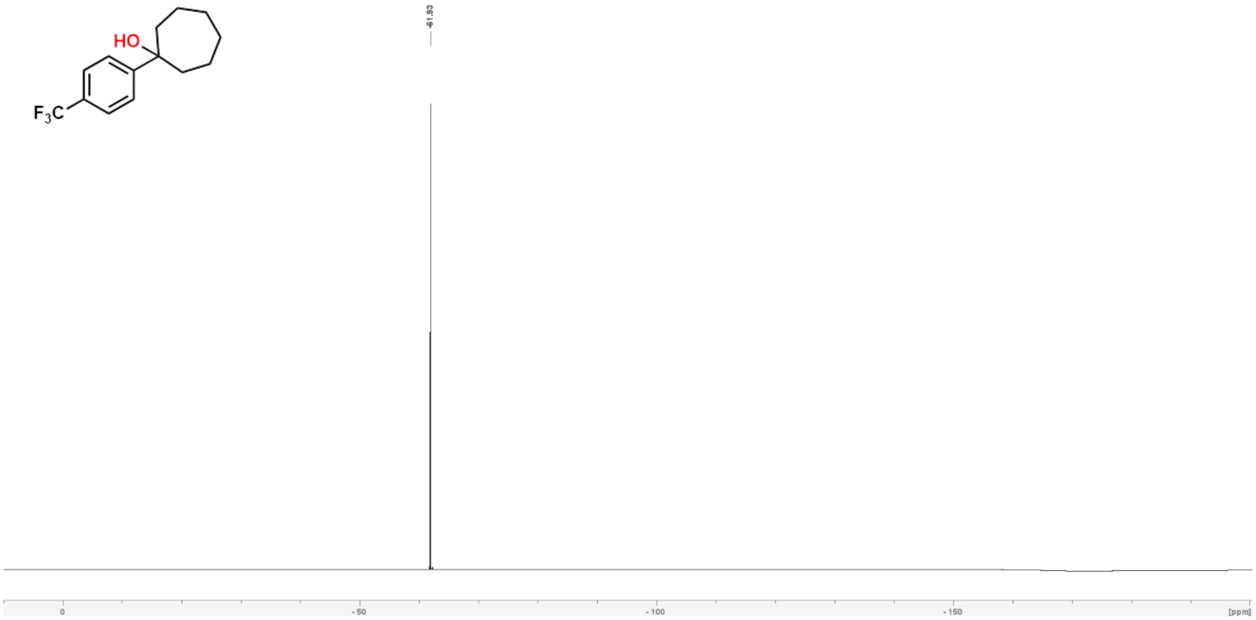

^{1}H NMR (600MHz, C_6D_6) of 2a combined isomers

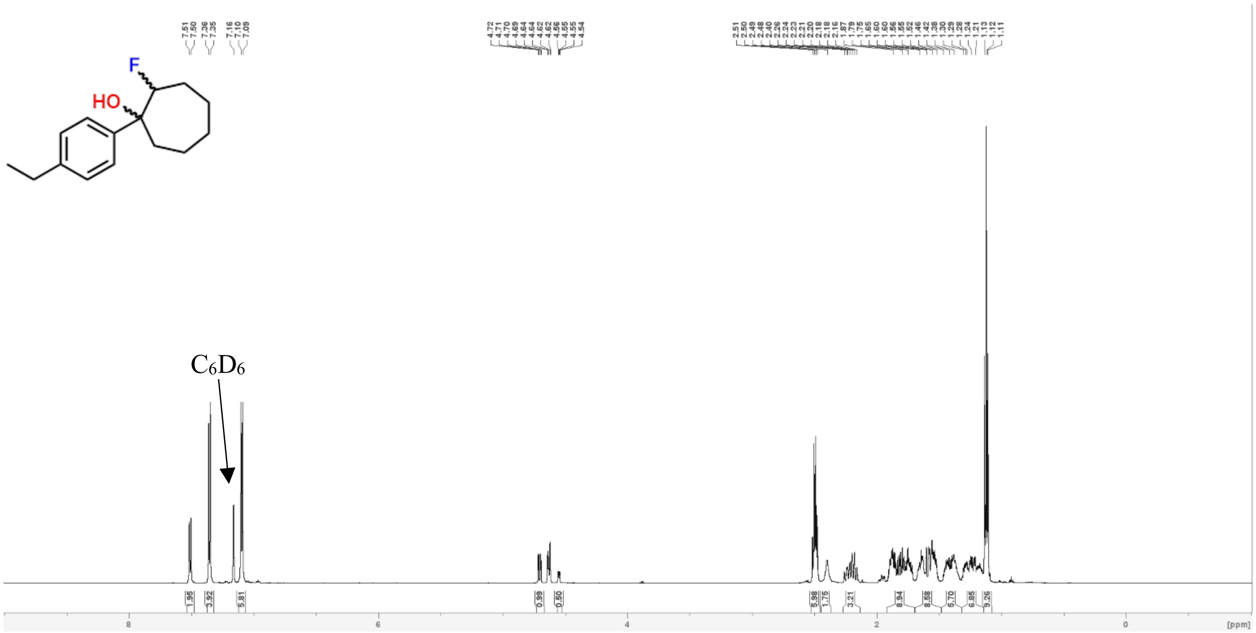

^{1}H NMR (600MHz, C_6D_6) of major isomer 2aa

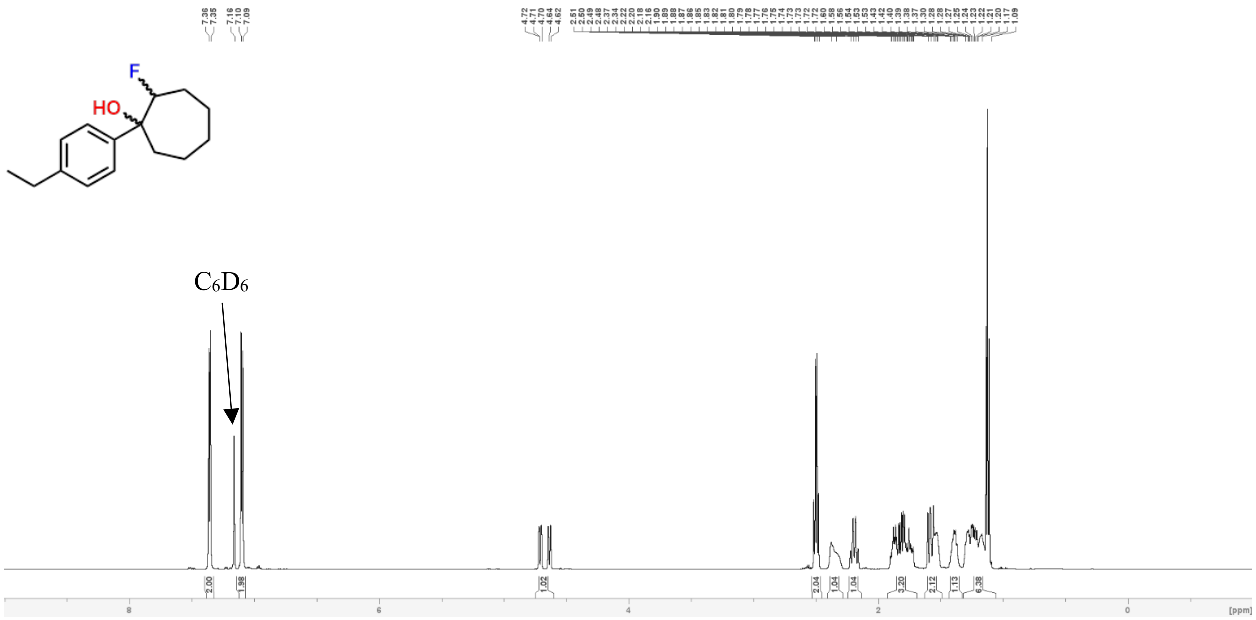

$^{13}C\{^{1}H\}$ NMR (150MHz, C_6D_6) of major isomer 2aa

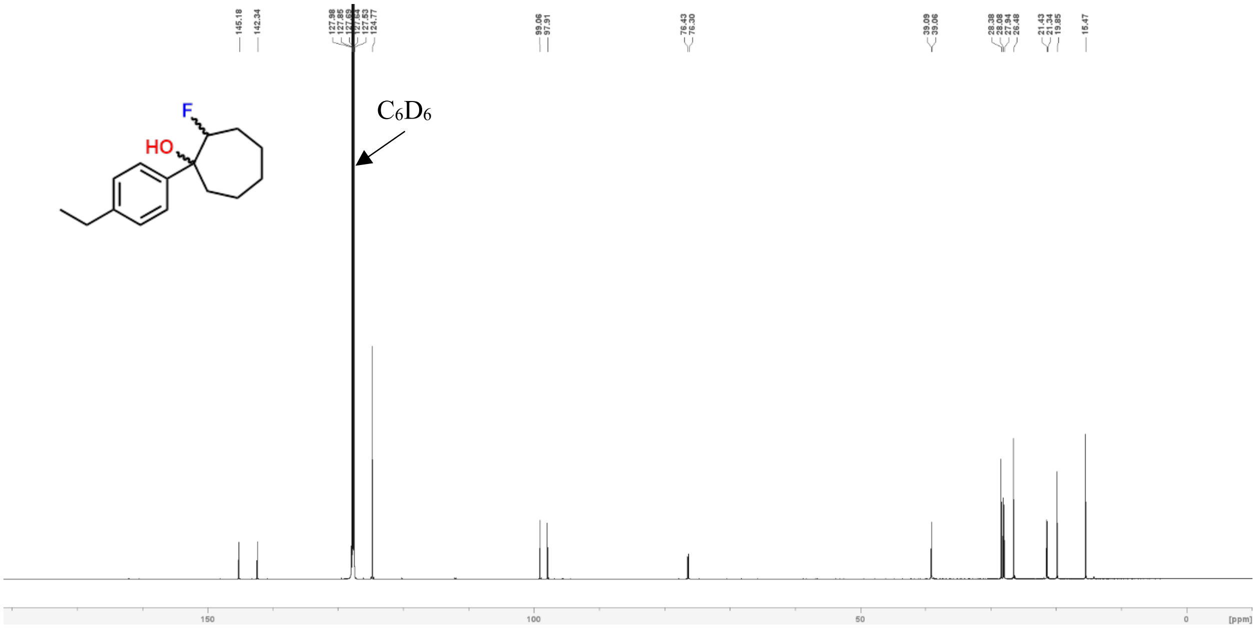

^{19}F NMR (565MHz, C_6D_6) of major isomer 2aa

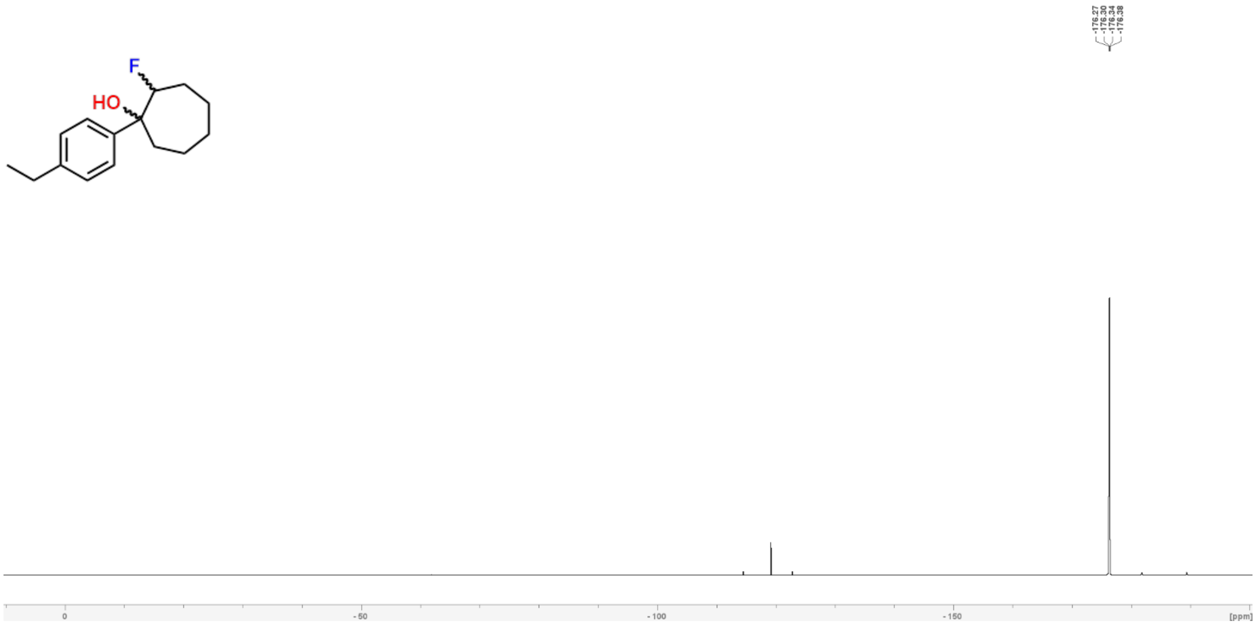

^{1}H NMR (600MHz, C_6D_6) of minor isomer 2ab

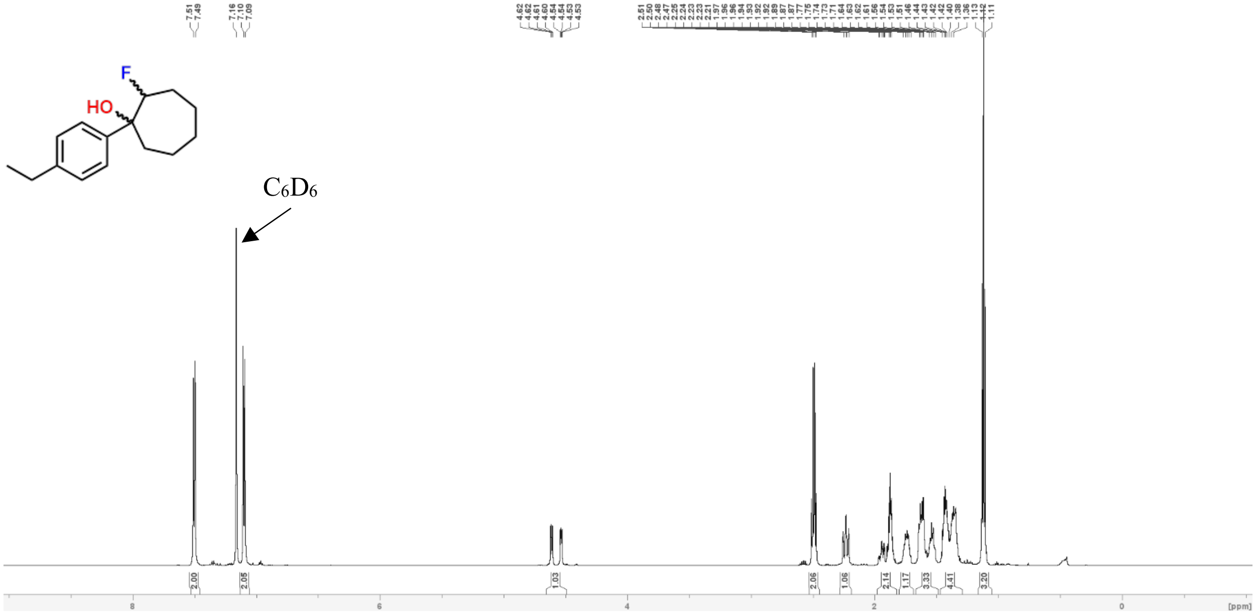

$^{13}C\{^{1}H\}$ NMR (150MHz, C_6D_6) of minor isomer 2ab

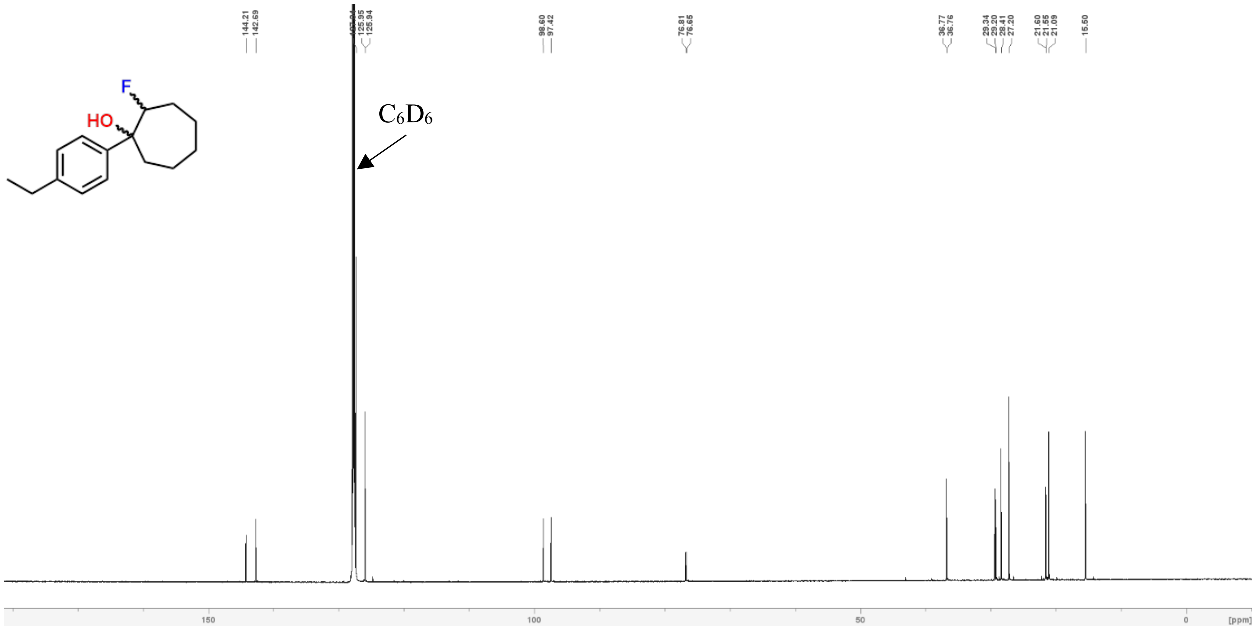

^{19}F NMR (565MHz, C_6D_6) of minor isomer 2ab

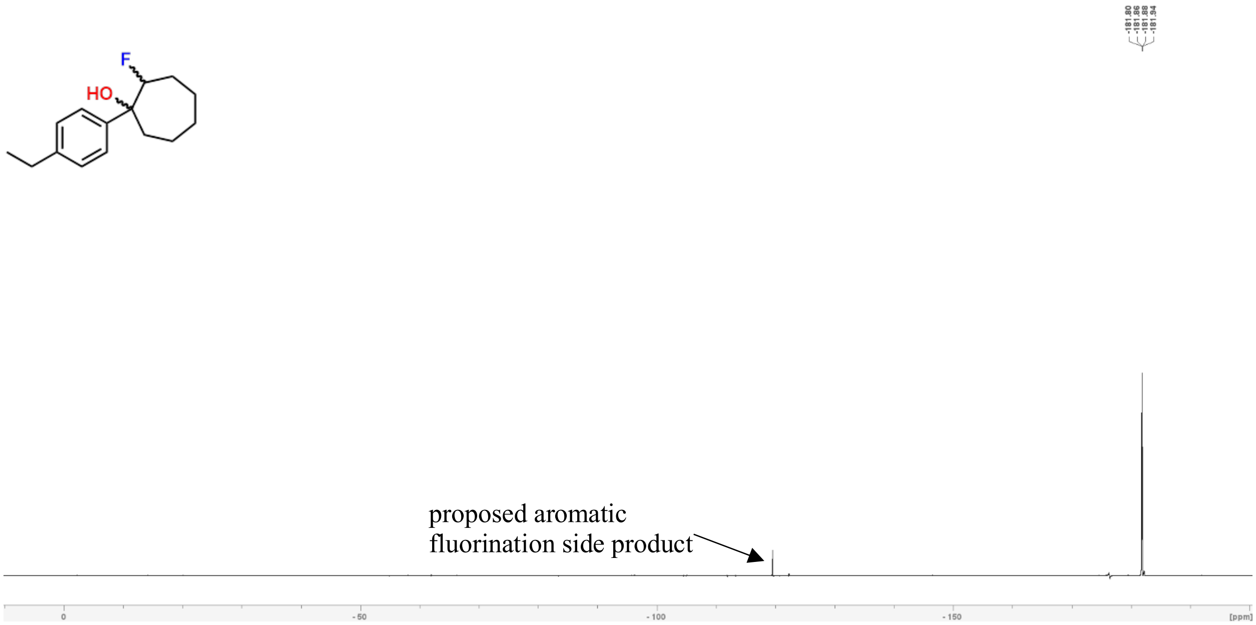

^{1}H NMR (600MHz, C_6D_6) of major isomer 2ba

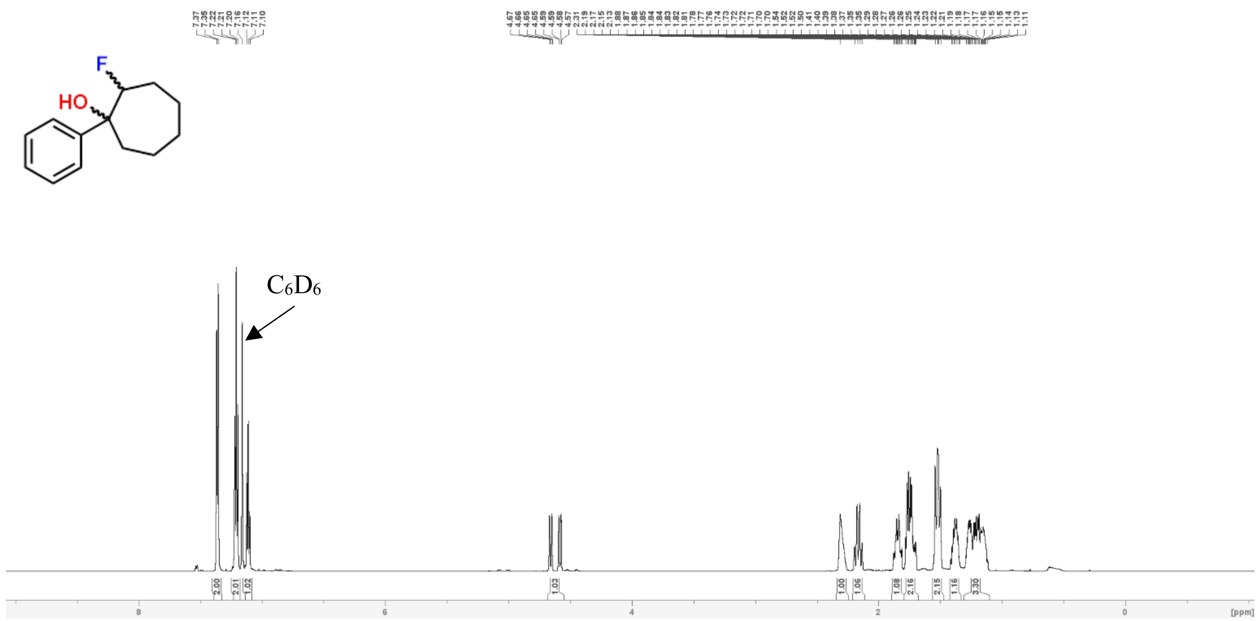

^{13}C{^{1}H} NMR (150MHz, C_6D_6) of major isomer 2ba

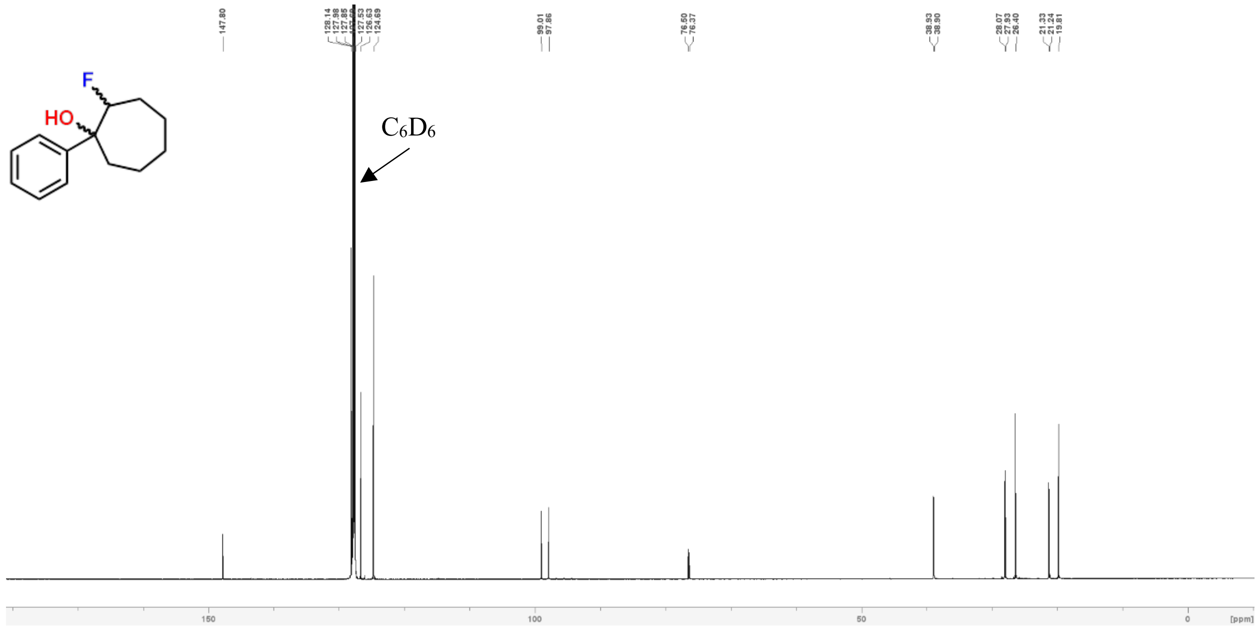

^{19}F NMR (565MHz, C_6D_6) of major isomer 2ba

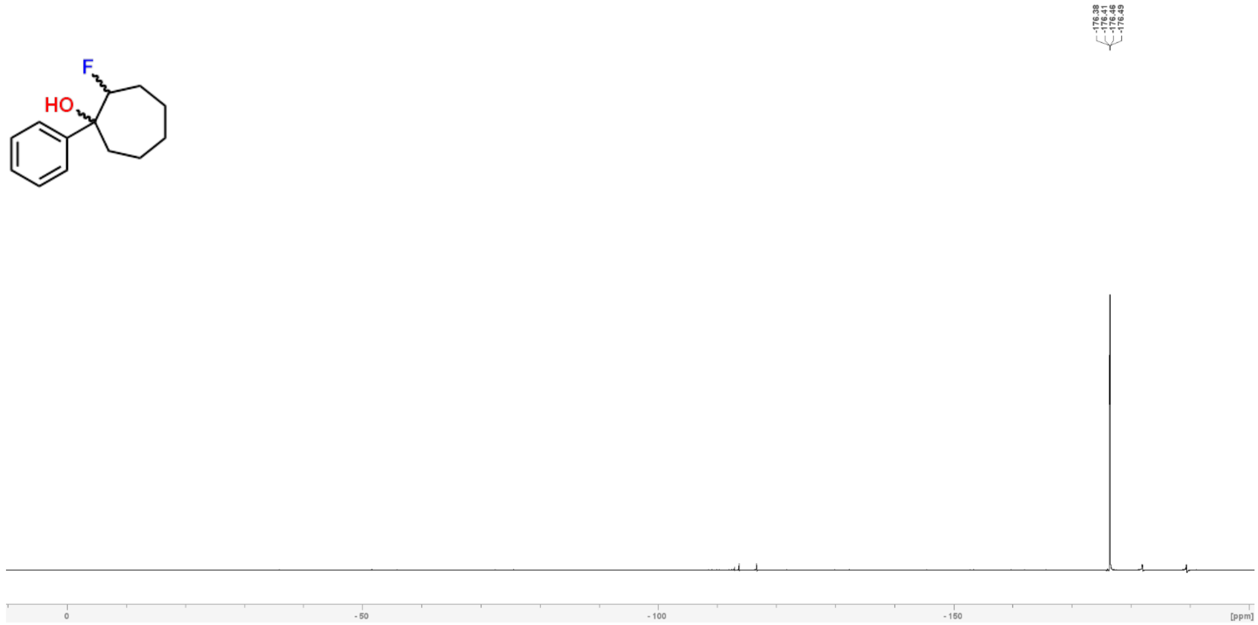

^{1}H NMR (600MHz, C_6D_6) of minor isomer 2bb

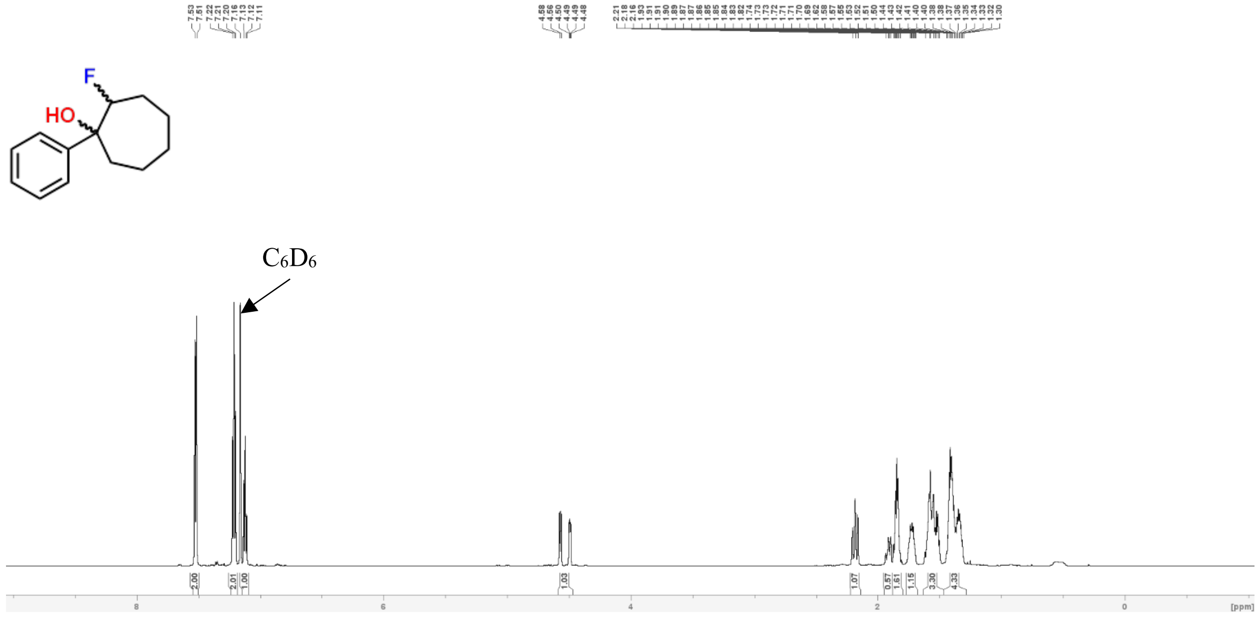

$^{13}C\{^{1}H\}$ NMR (150MHz, C_6D_6) of minor isomer 2bb

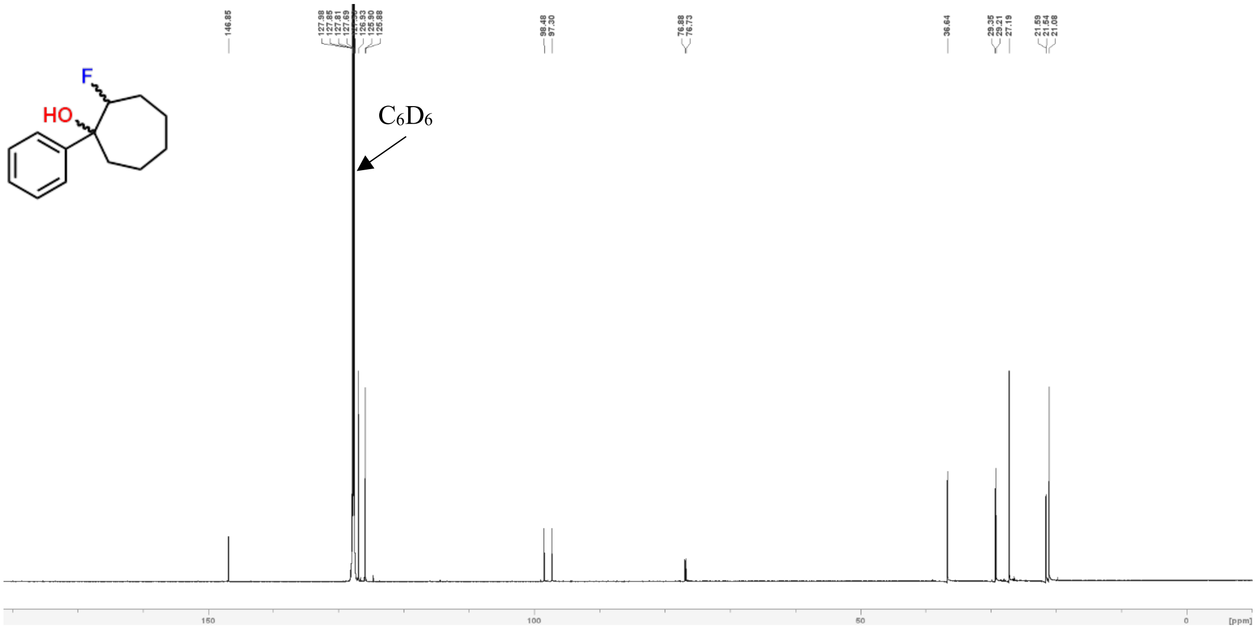

^{19}F NMR (565MHz, C_6D_6) of minor isomer 2bb

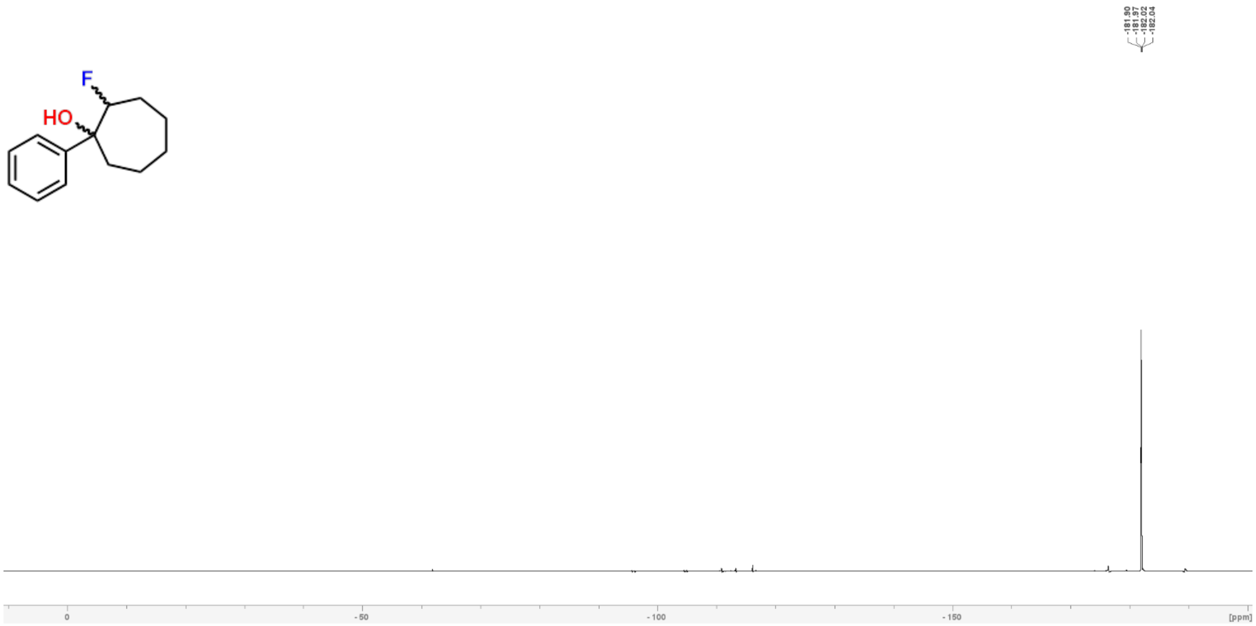

^{1}H NMR (600MHz, C_6D_6) of major isomer 2c

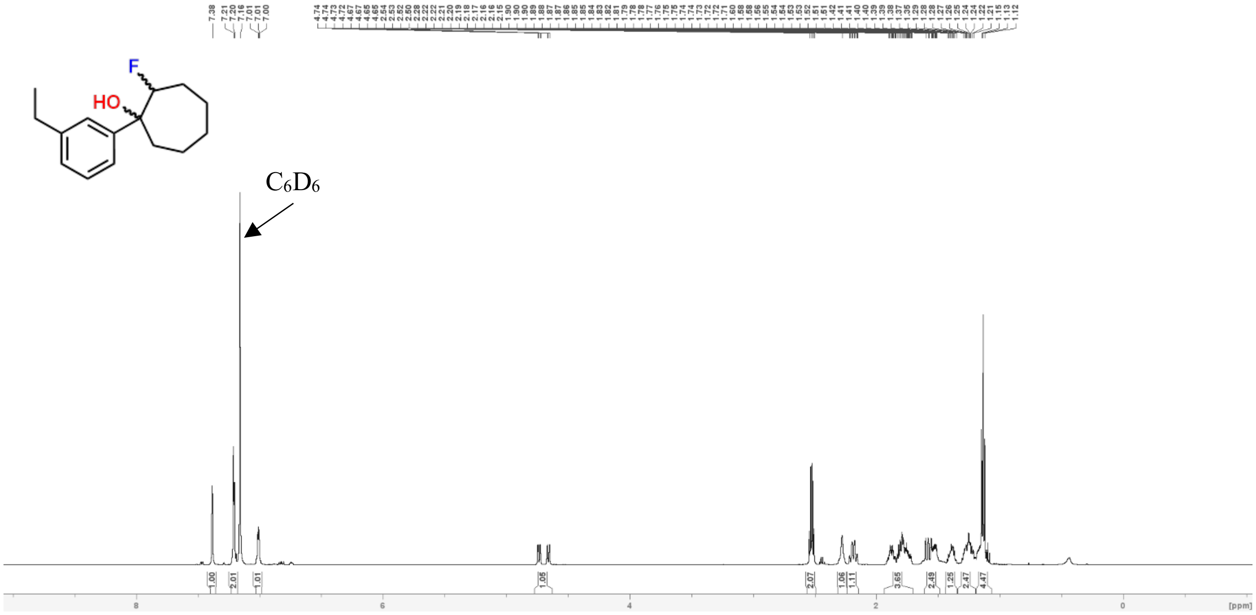

^{13}C{^{1}H} NMR (150MHz, C_6D_6) of major isomer 2c

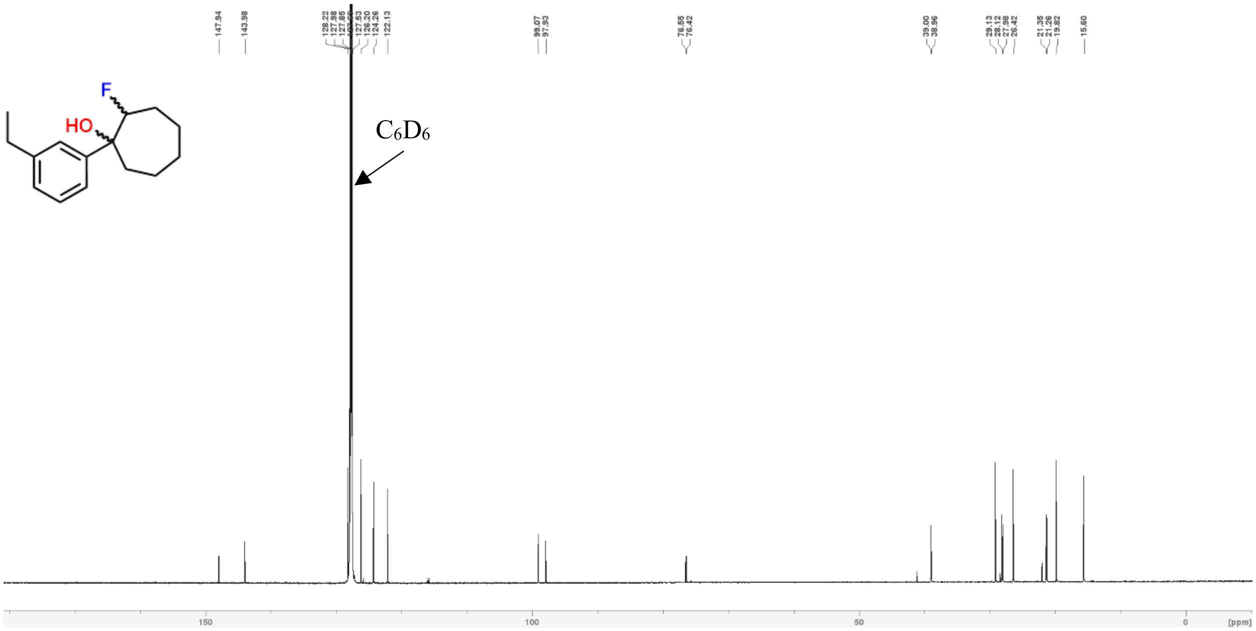

^{19}F NMR (565MHz, C_6D_6) of major isomer 2c

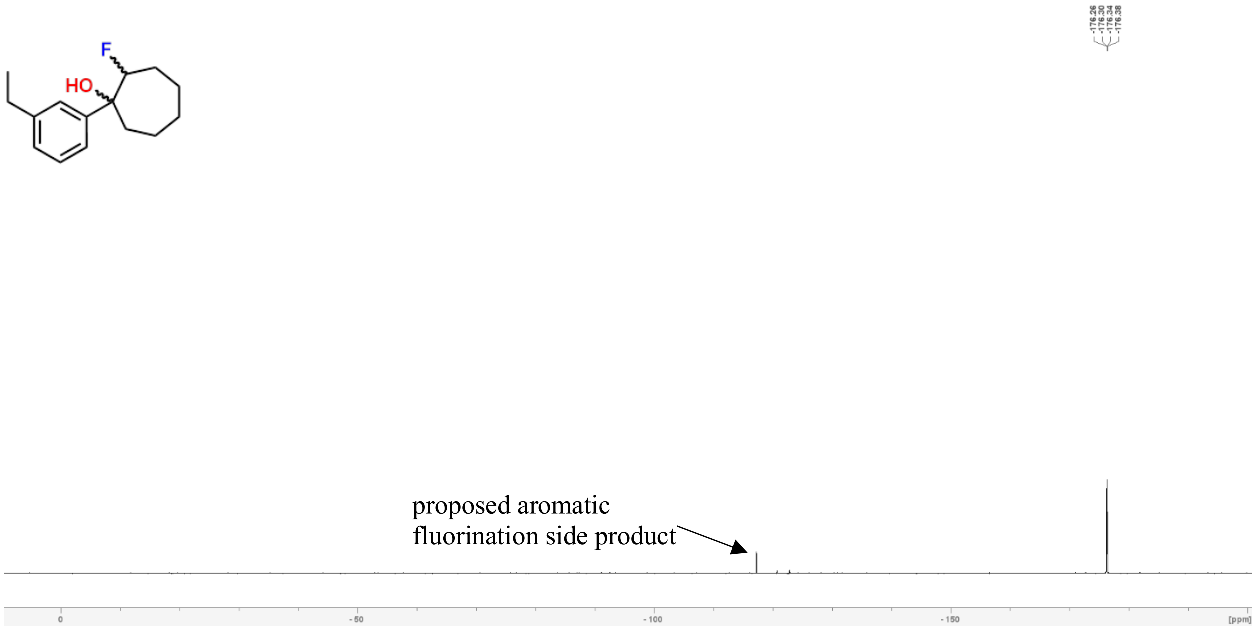

^{1}H NMR (600MHz, C_6D_6) of major isomer 2da

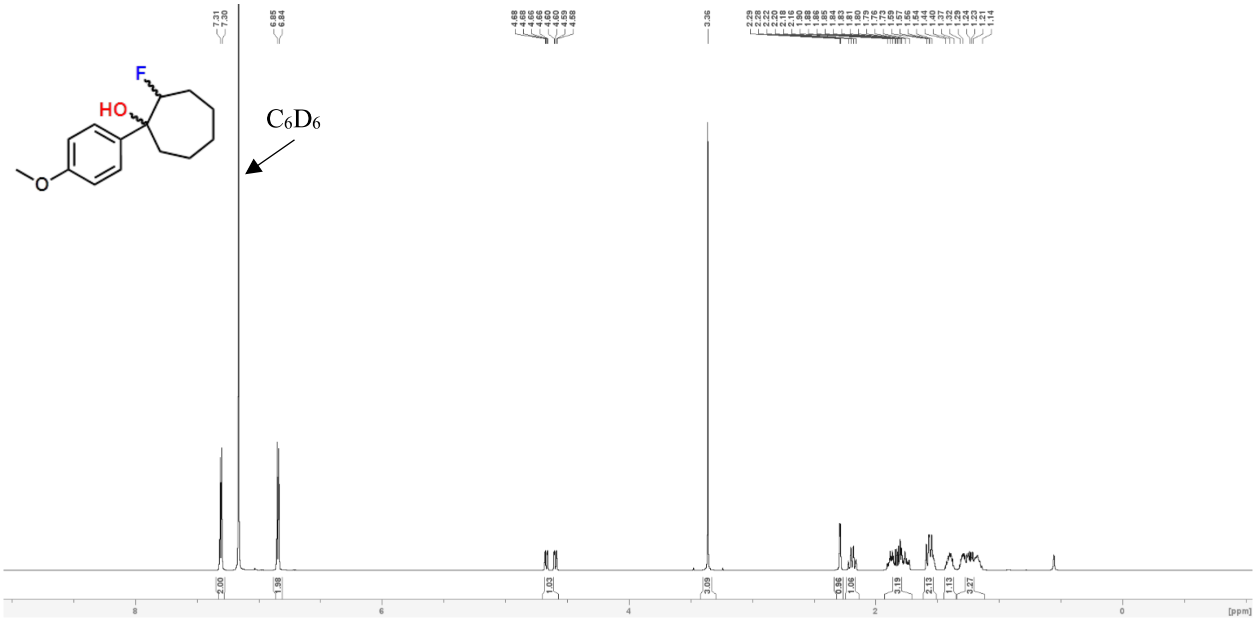

$^{13}C\{^{1}H\}$ NMR (150MHz, C_6D_6) of major isomer 2da

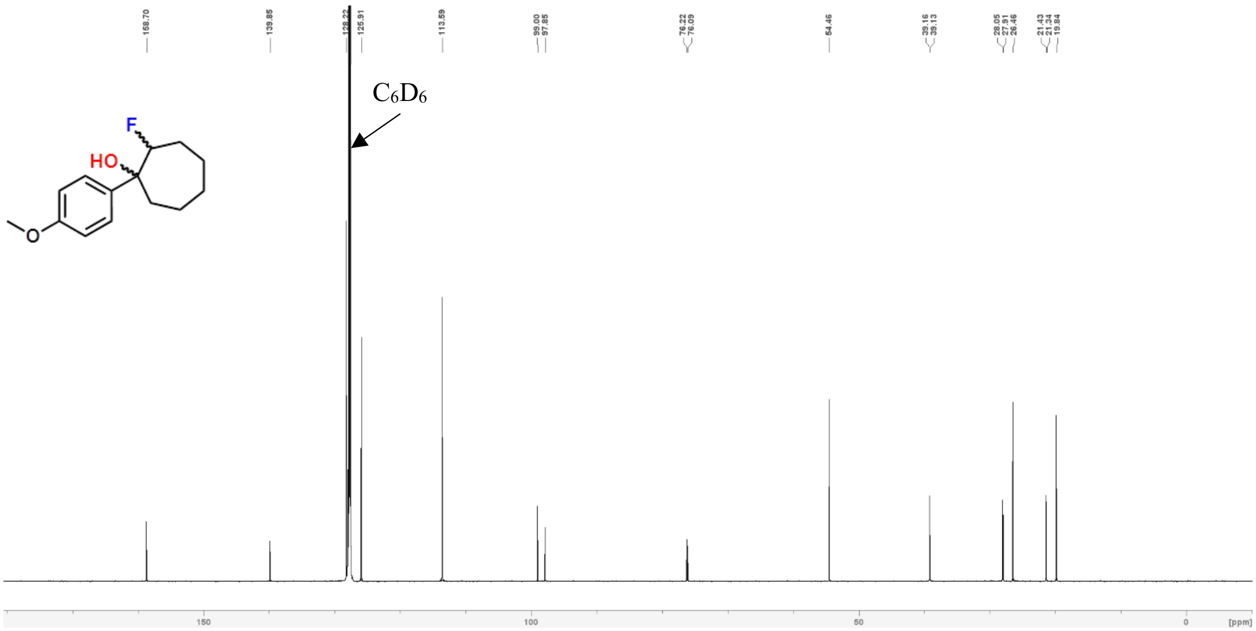

^{19}F NMR (565MHz, C_6D_6) of major isomer 2da

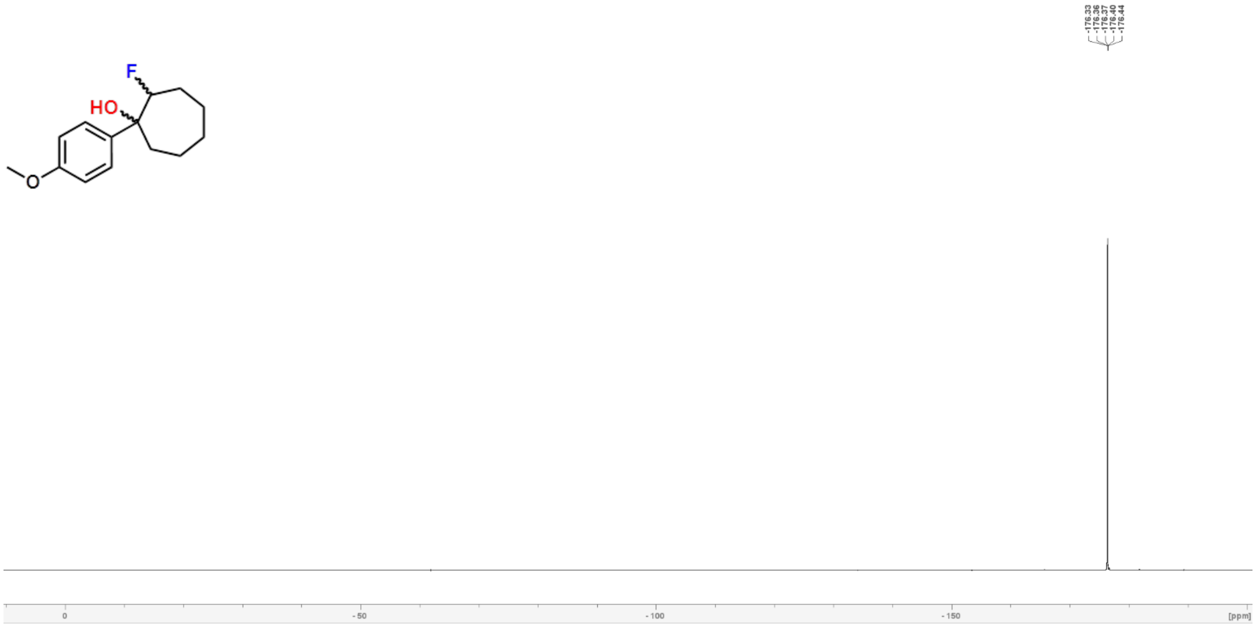

^{1}H NMR (600MHz, C_6D_6) of minor isomer 2db

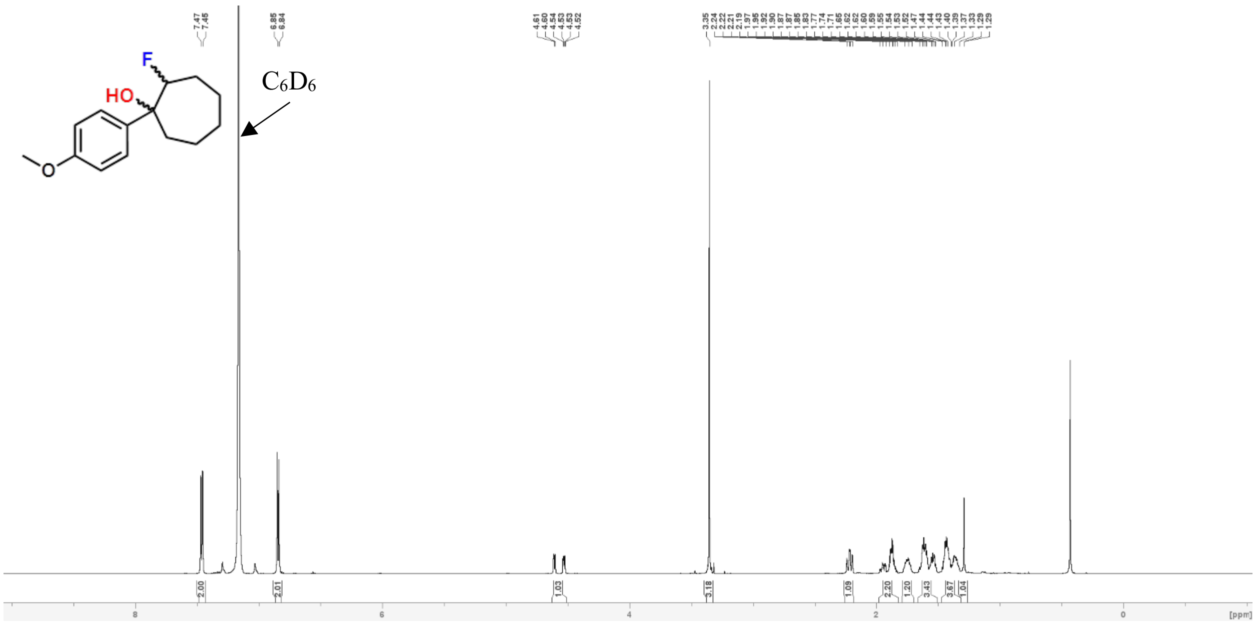

$^{13}C\{^{1}H\}$ NMR (150MHz, C_6D_6) of minor isomer 2db

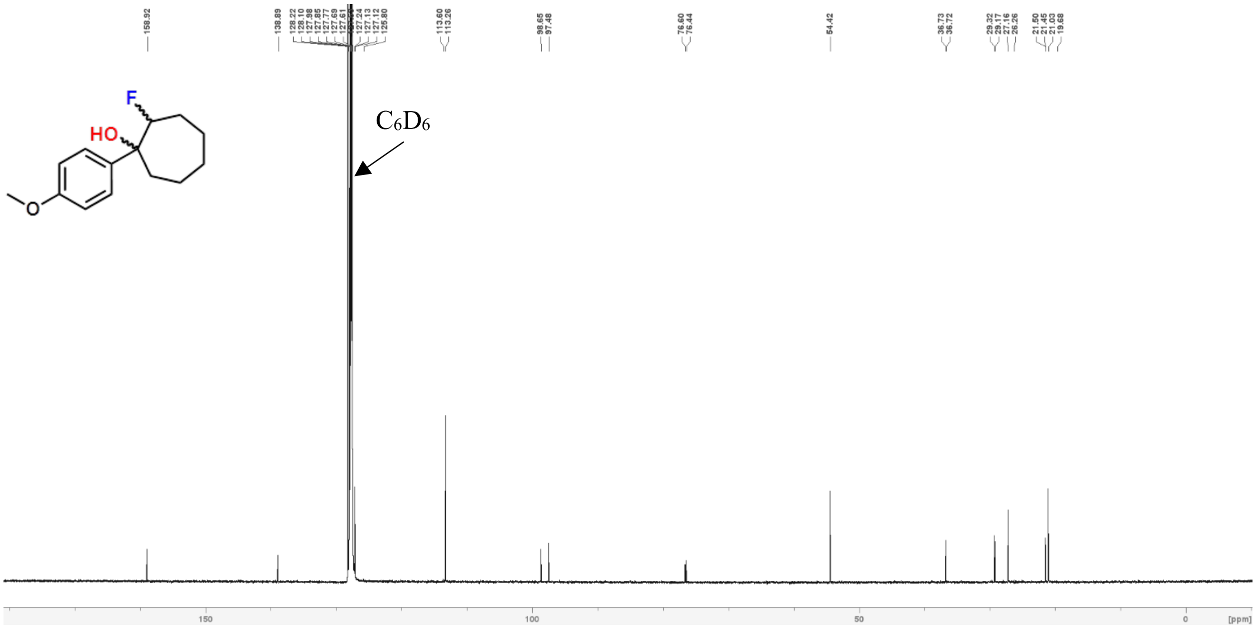

^{19}F NMR (565MHz, C_6D_6) of minor isomer 2db

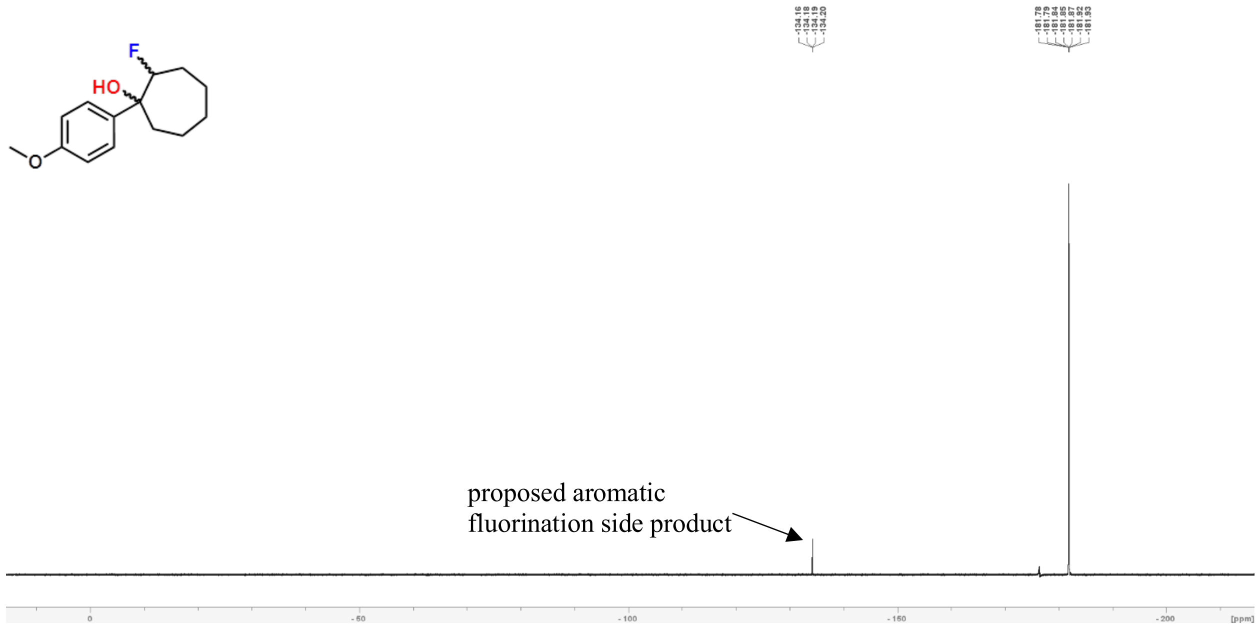

^{1}H NMR (600MHz, C_6D_6) of major isomer 2e

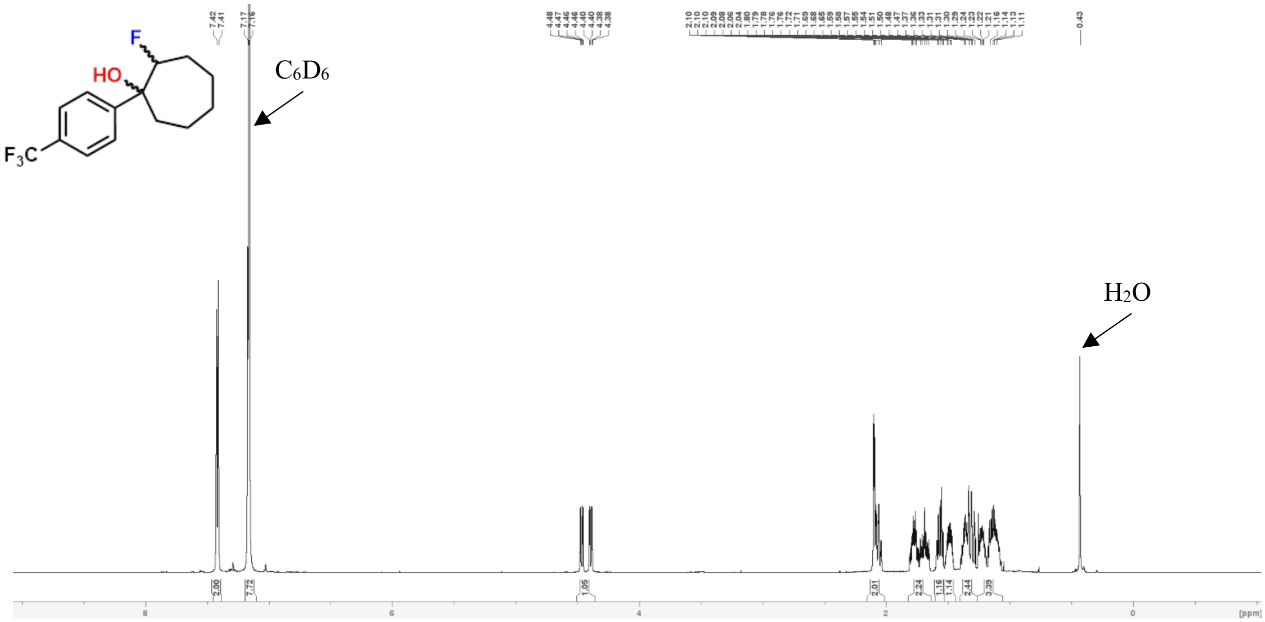

$^{13}C\{^1H\}$ NMR (150MHz, C_6D_6) of major isomer 2e

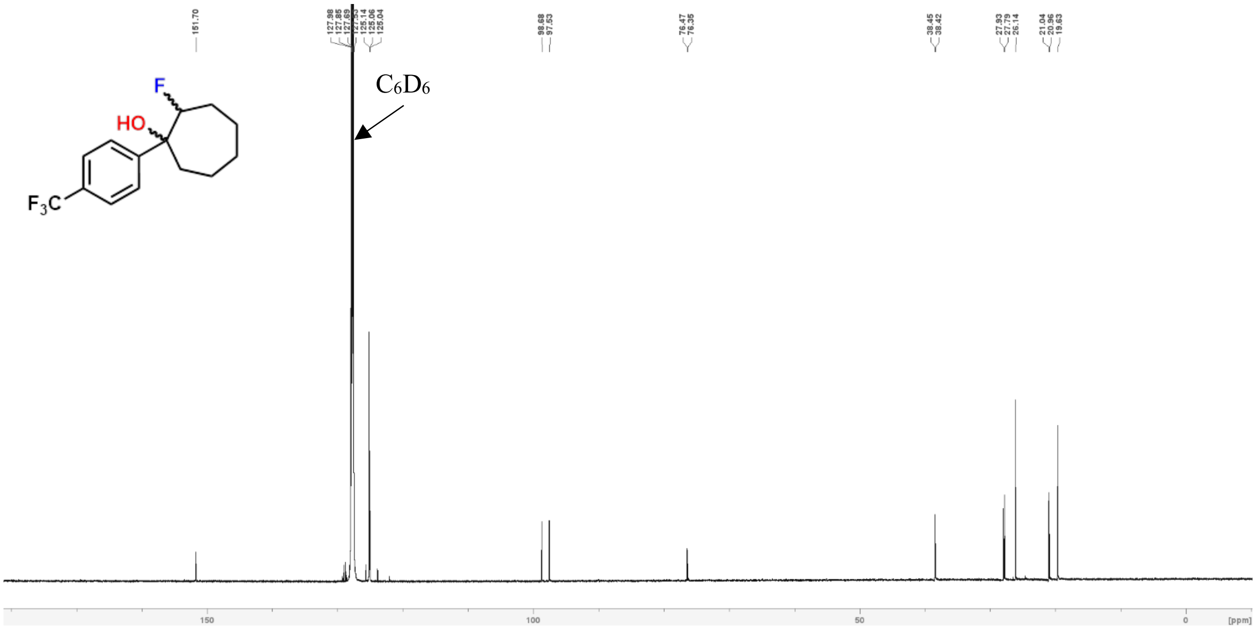

^{19}F NMR (565MHz, C_6D_6) of major isomer 2e

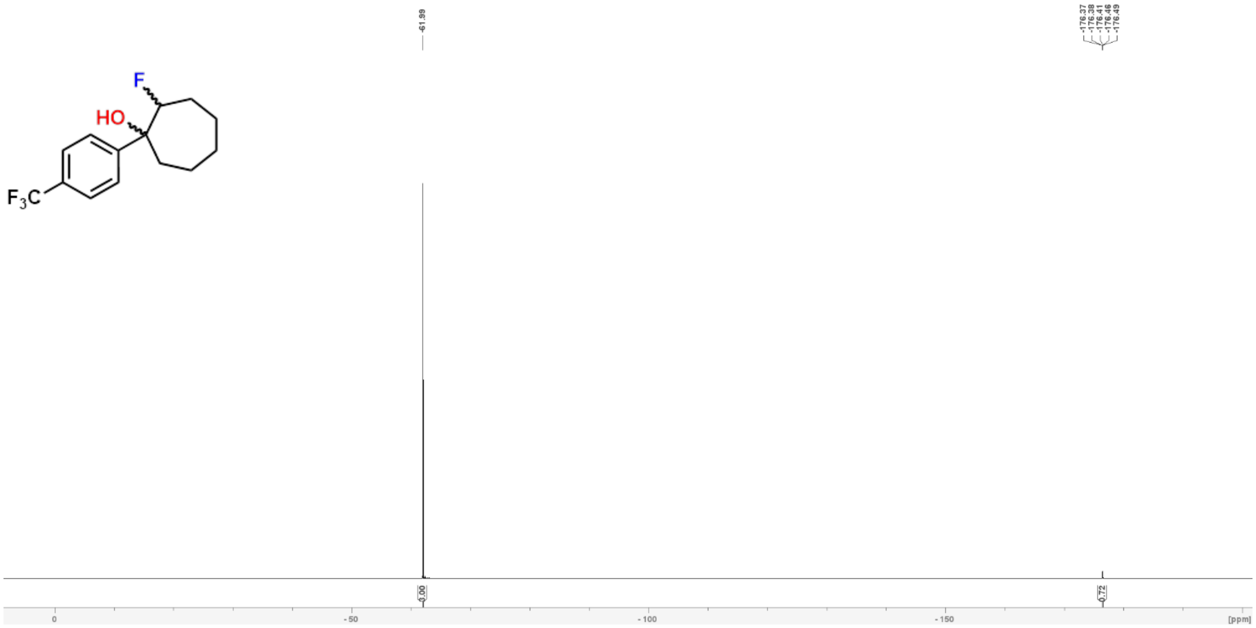

^{1}H NMR (600MHz, C_6D_6) of 3a

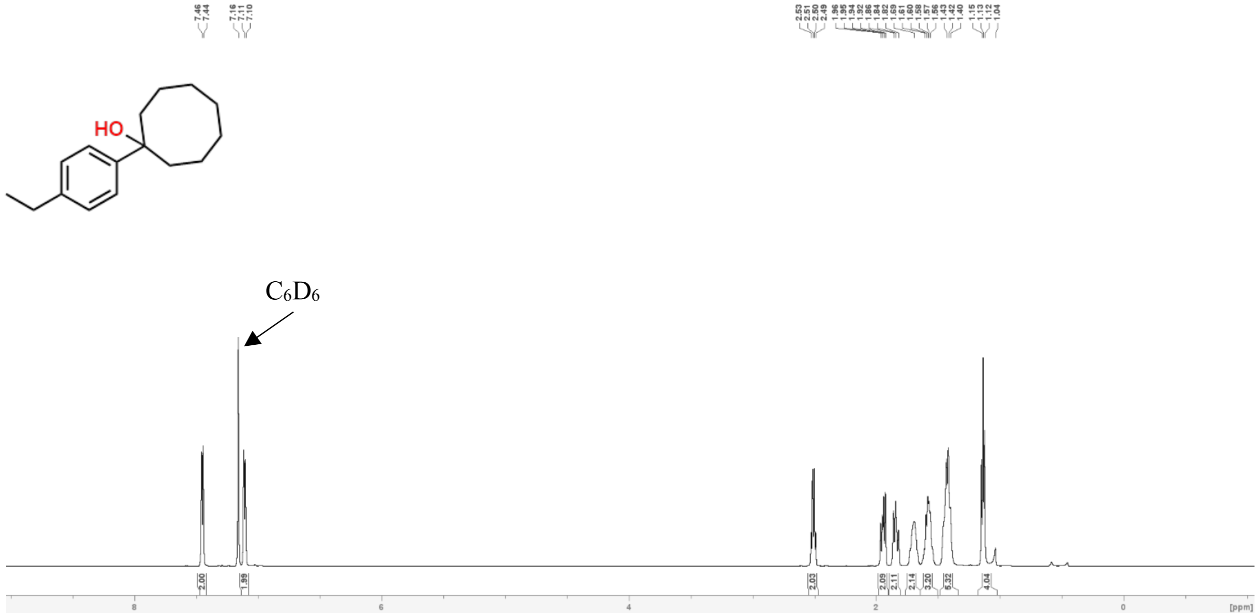

$^{13}C\{^{1}H\}$ NMR (150MHz, C_6D_6) of 3a

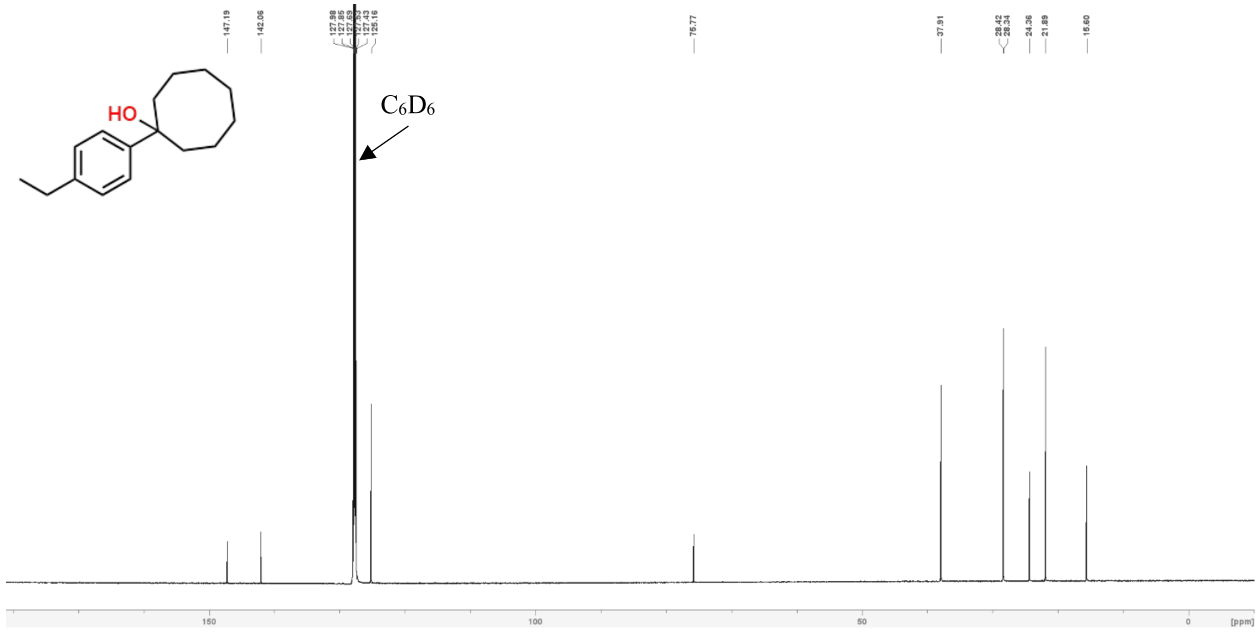

^{1}H NMR (600MHz, C_6D_6) of 3b

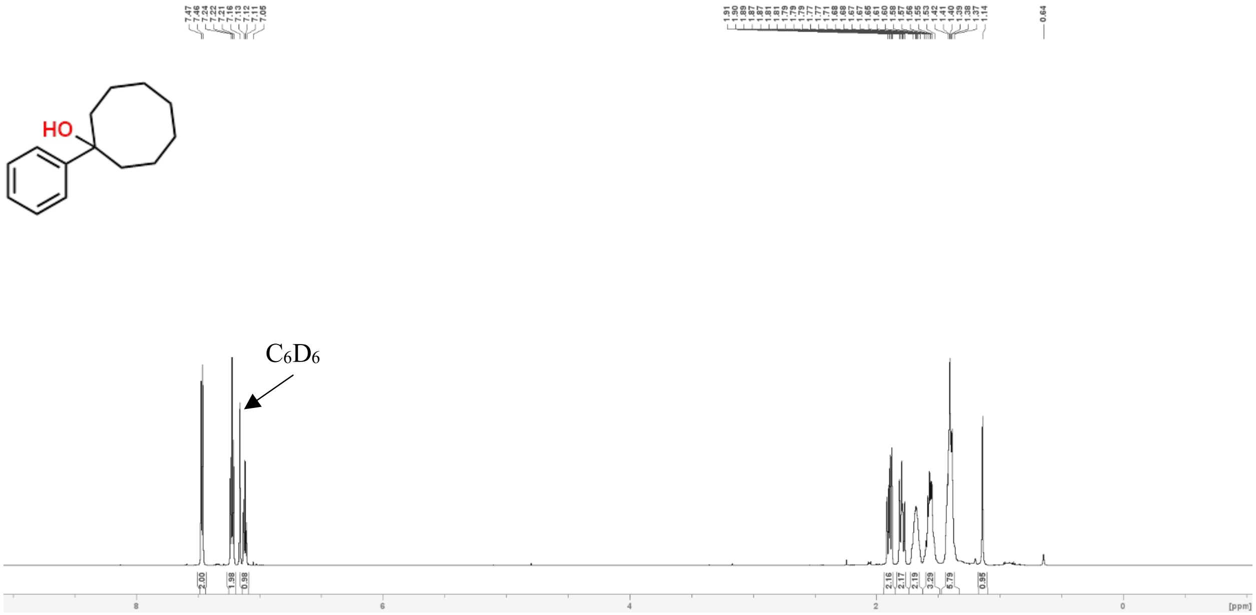

^{13}C{^{1}H} NMR (150MHz, C_6D_6) of 3b

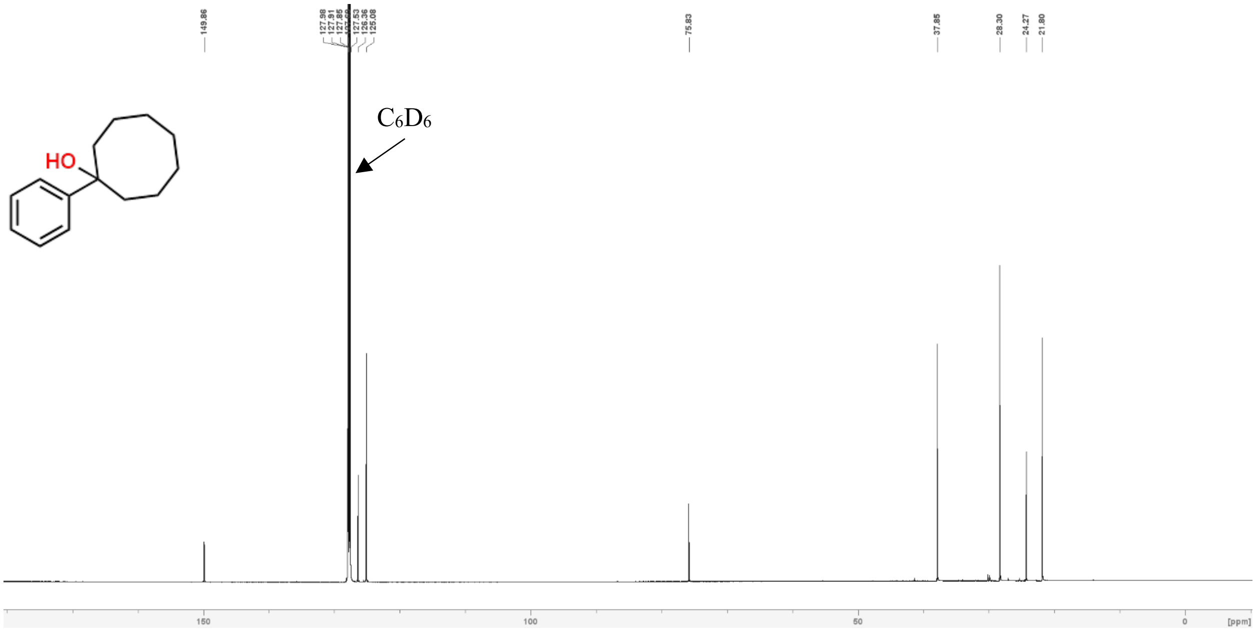

^{1}H NMR (600MHz, C_6D_6) of 3c

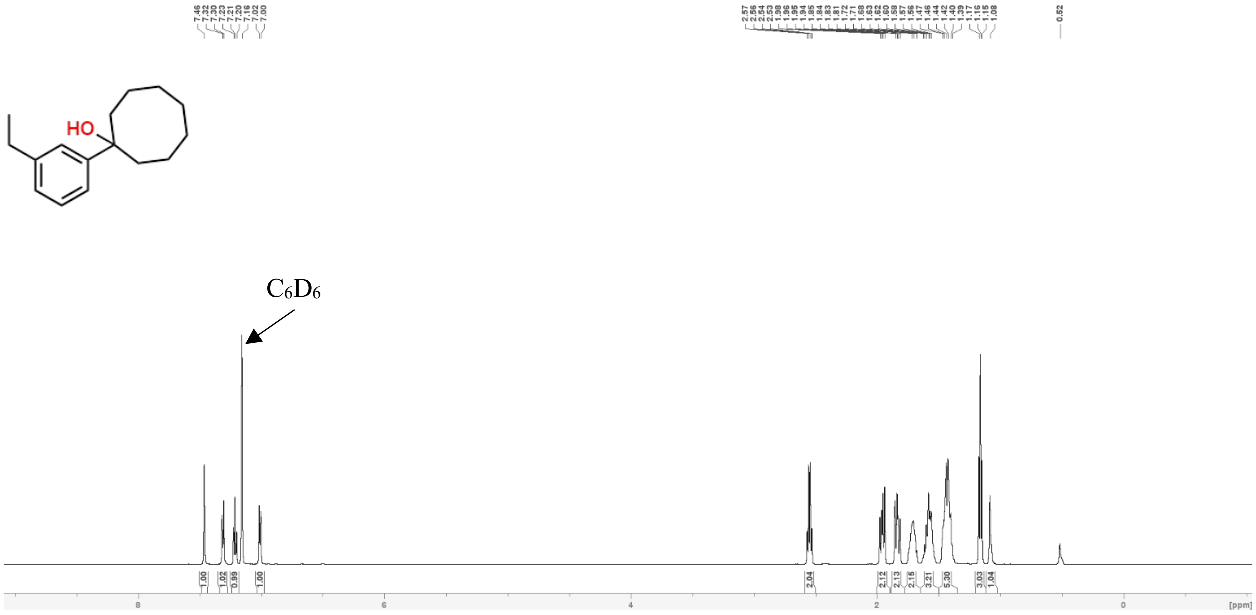

^{13}C{^{1}H} NMR (150MHz, C_6D_6) of 3c

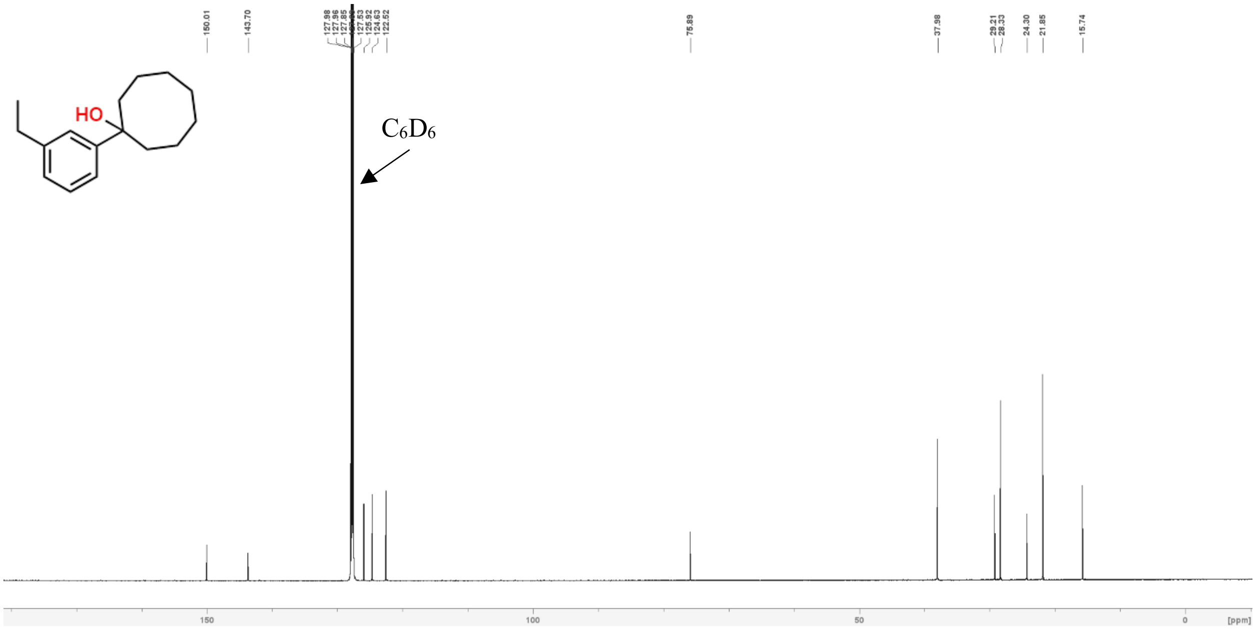

^{1}H NMR (600MHz, C_6D_6) of major isomer 4a

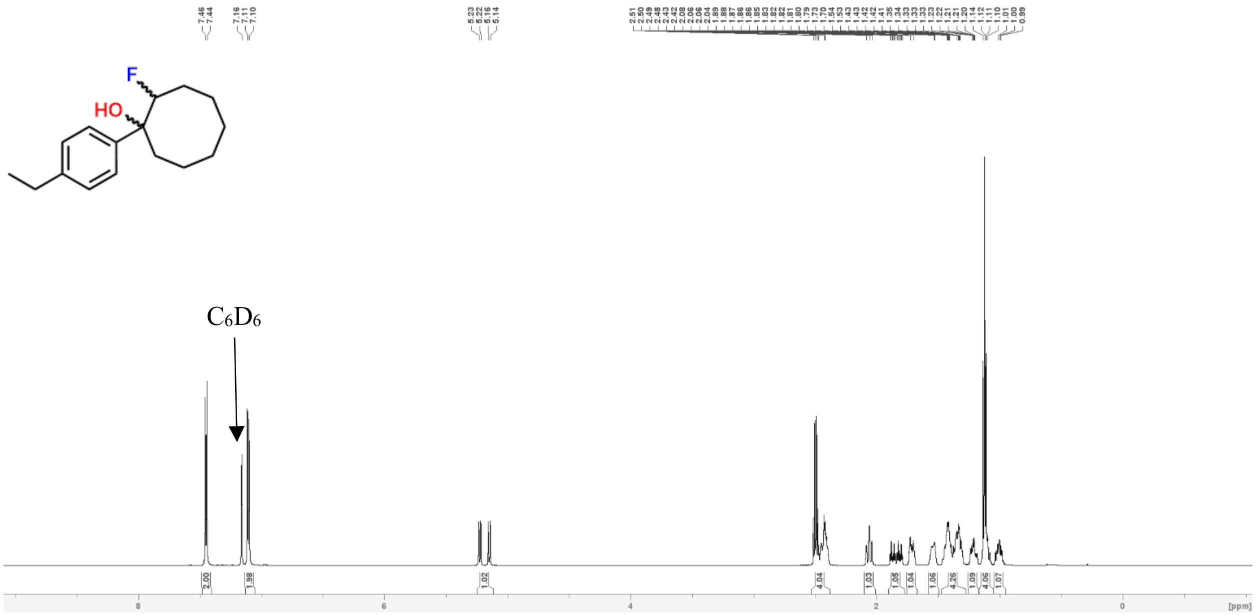

$^{13}C\{^{1}H\}$ NMR (150MHz, C_6D_6) of major isomer 4a

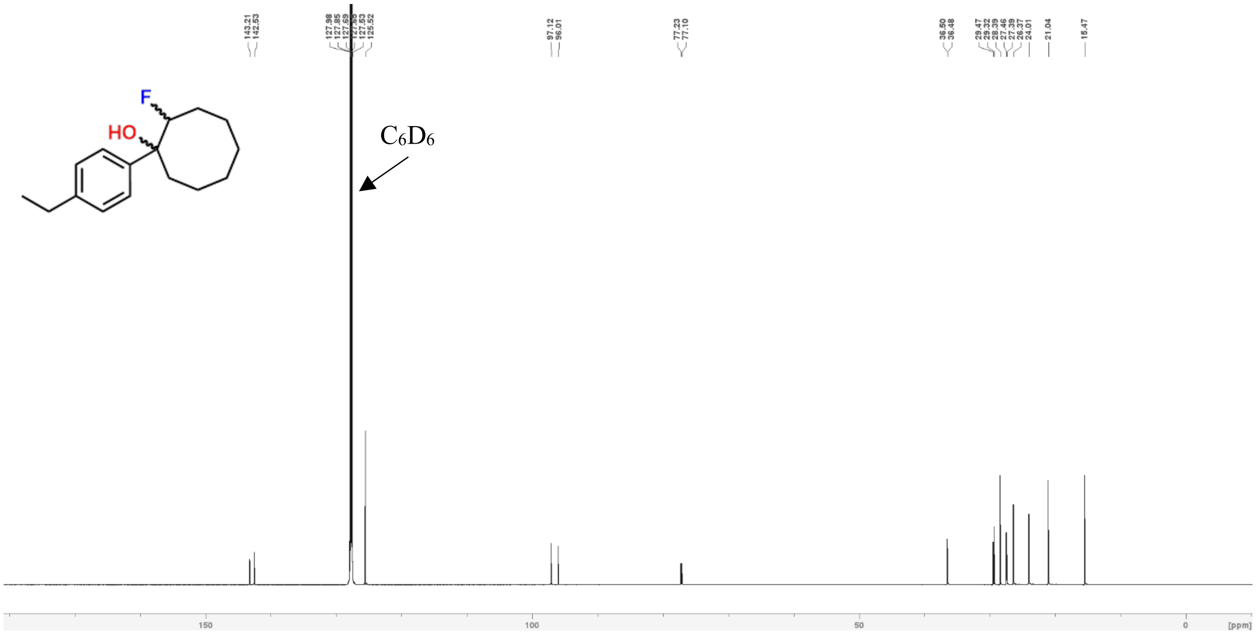

^{19}F NMR (565MHz, C_6D_6) of major isomer 4a

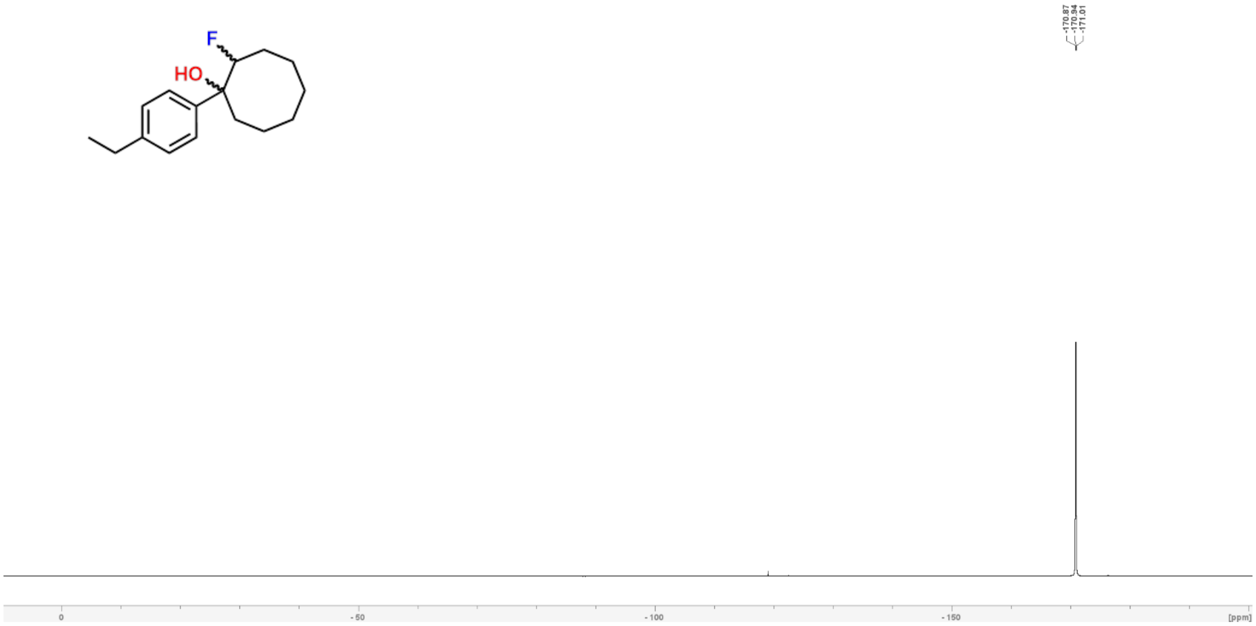

^{1}H NMR (600MHz, C_6D_6) of major isomer 4b

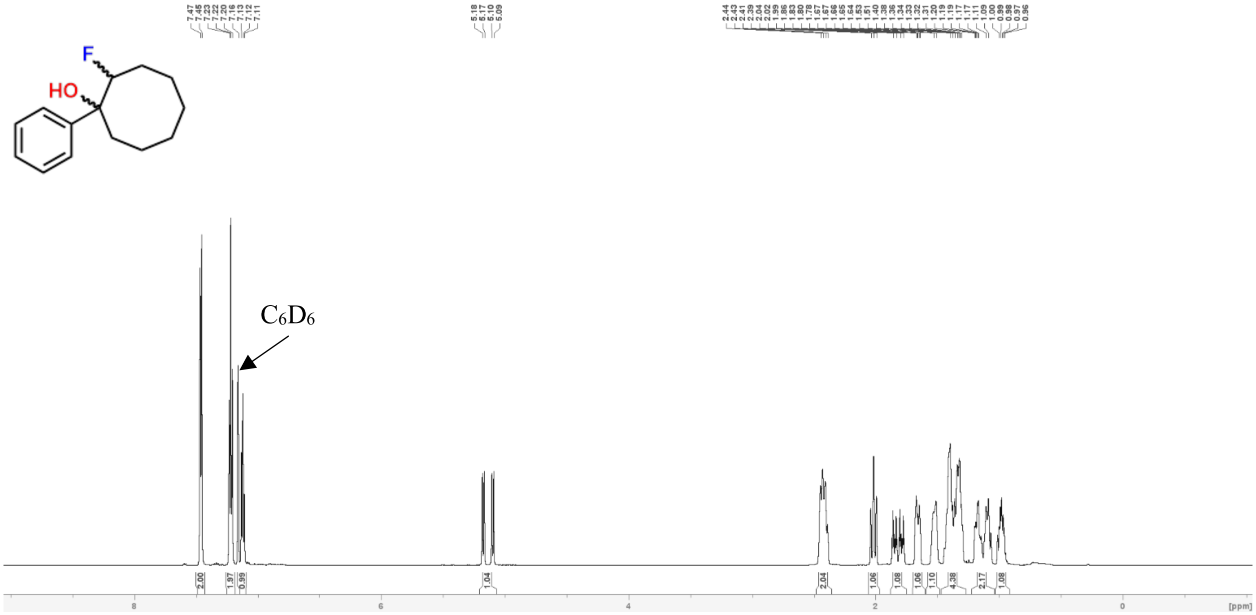

$^{13}C\{^{1}H\}$ NMR (150MHz, C_6D_6) of major isomer 4b

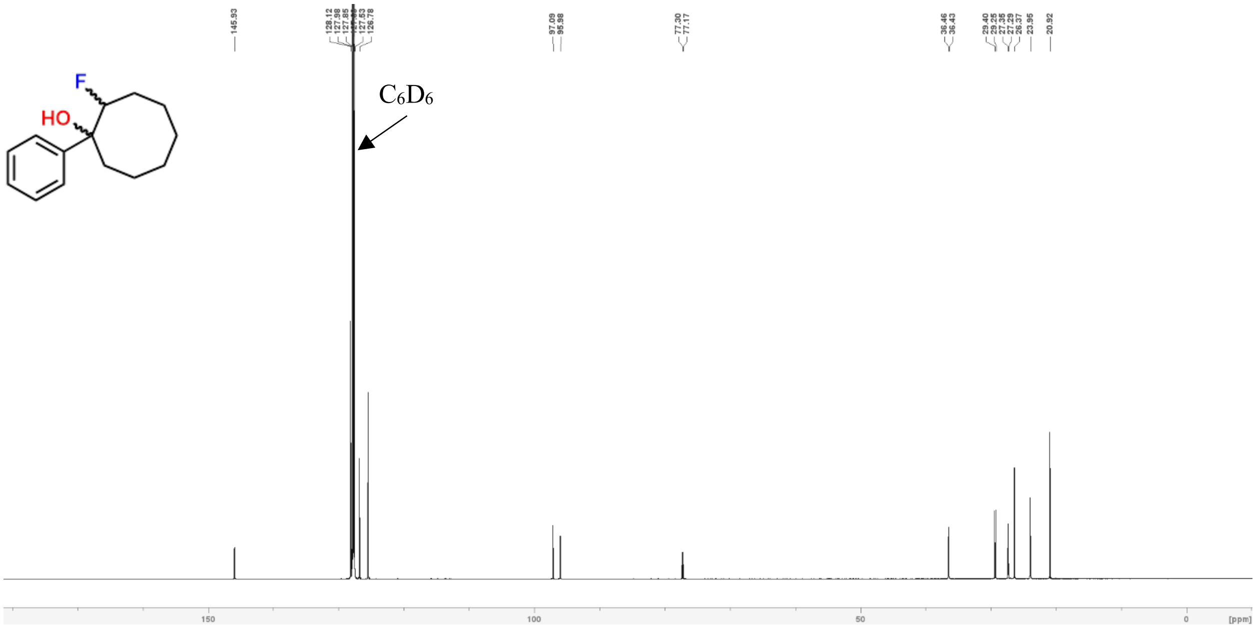

^{19}F NMR (565MHz, C_6D_6) of major isomer 4b

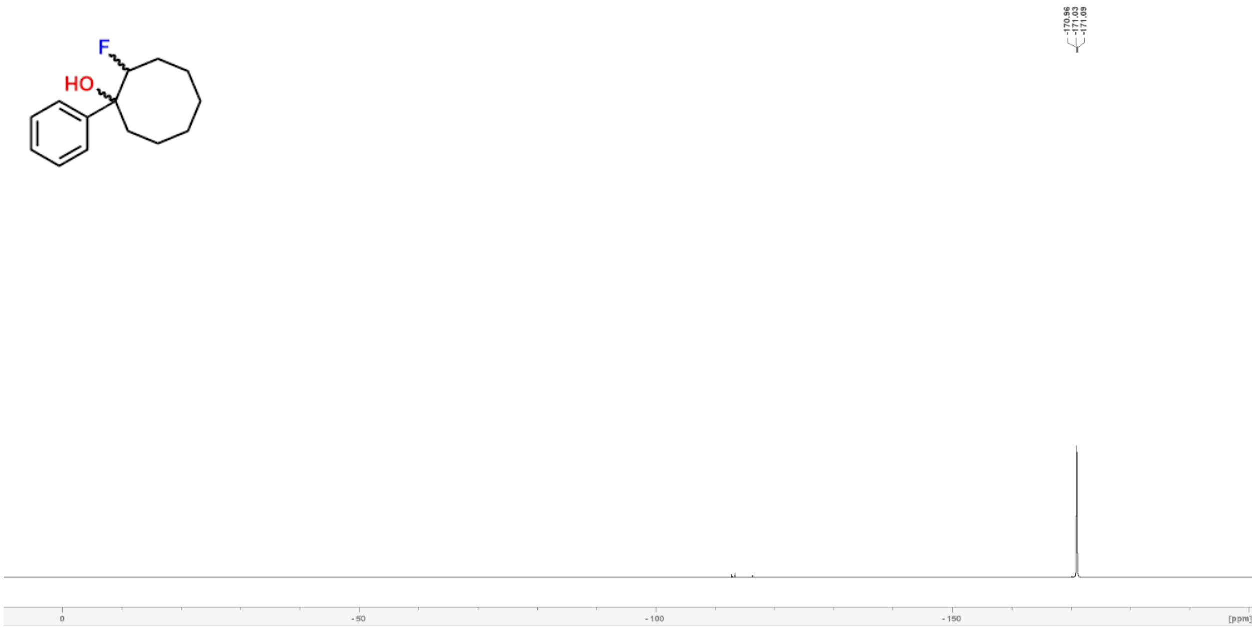

^{1}H NMR (600MHz, C_6D_6) of major isomer 4c

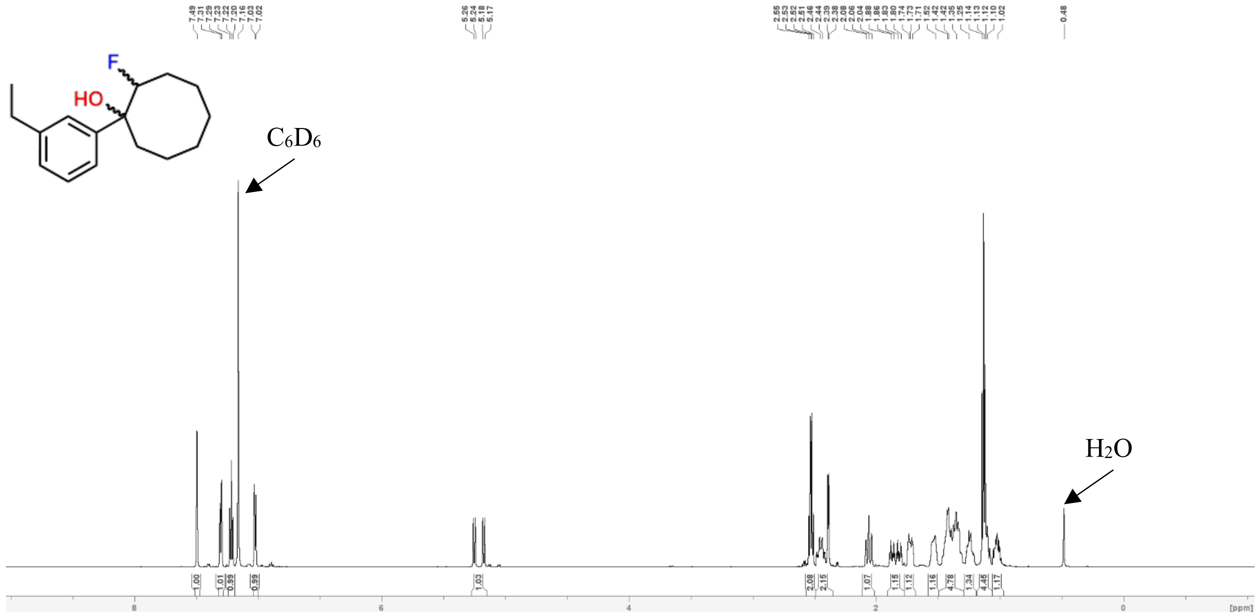

^{13}C{^{1}H} NMR (150MHz, C_6D_6) of major isomer 4c

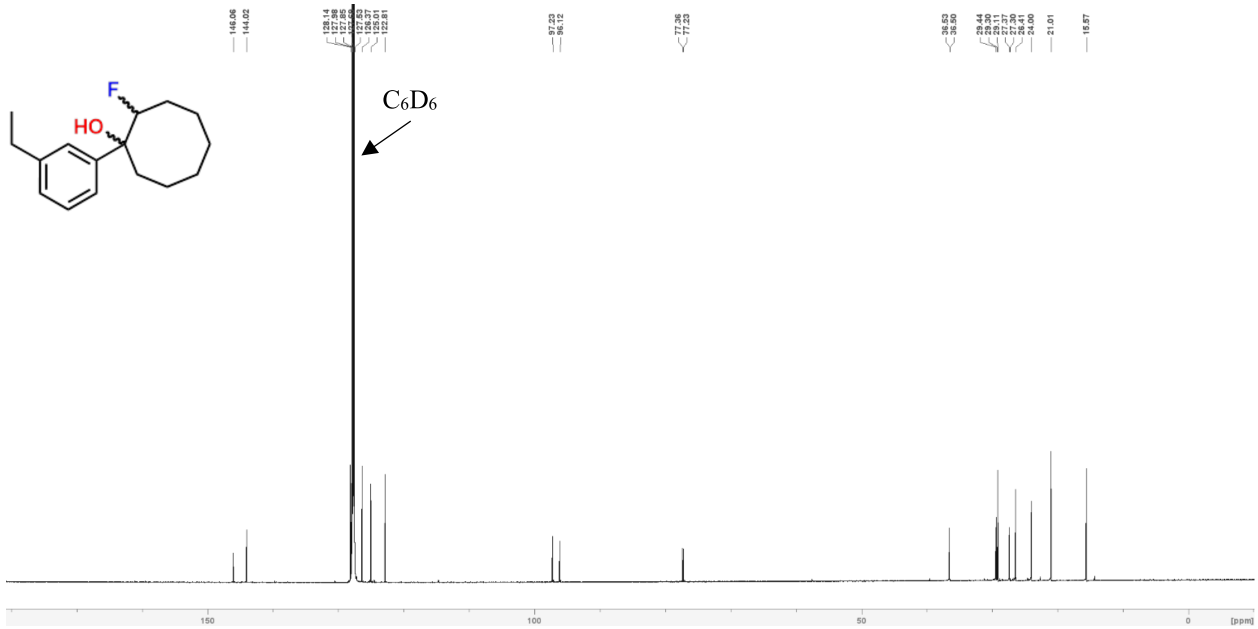

^{19}F NMR (565MHz, C_6D_6) of major isomer 4c

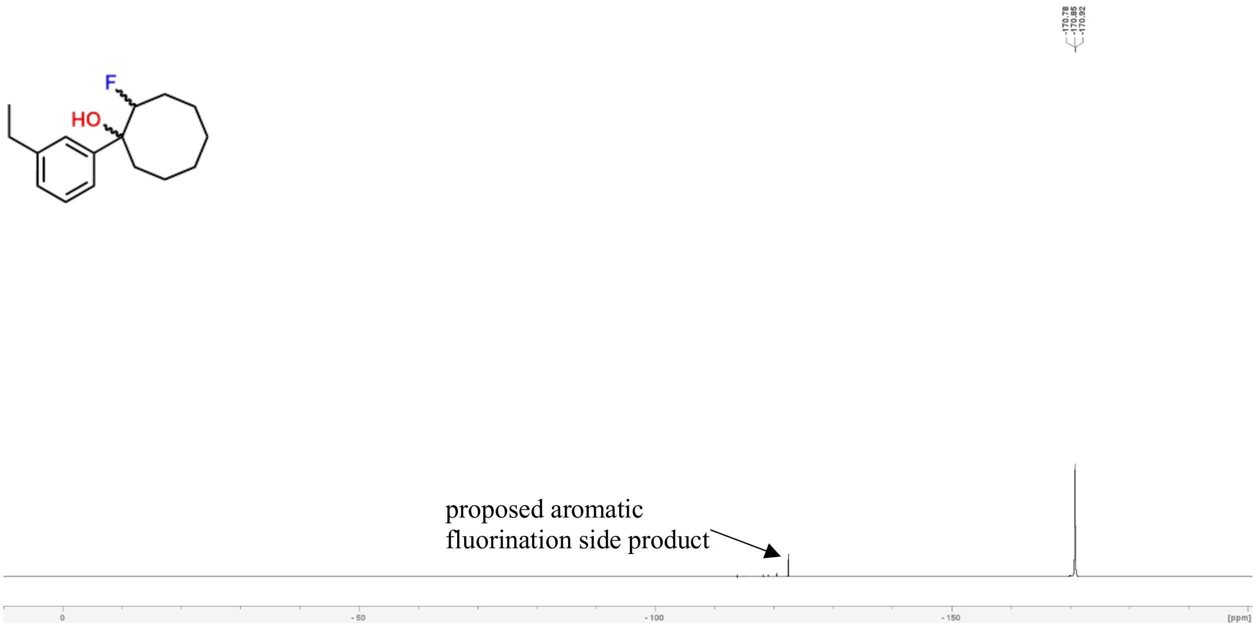

^{1}H NMR (600MHz, C_6D_6) of major isomer 4d

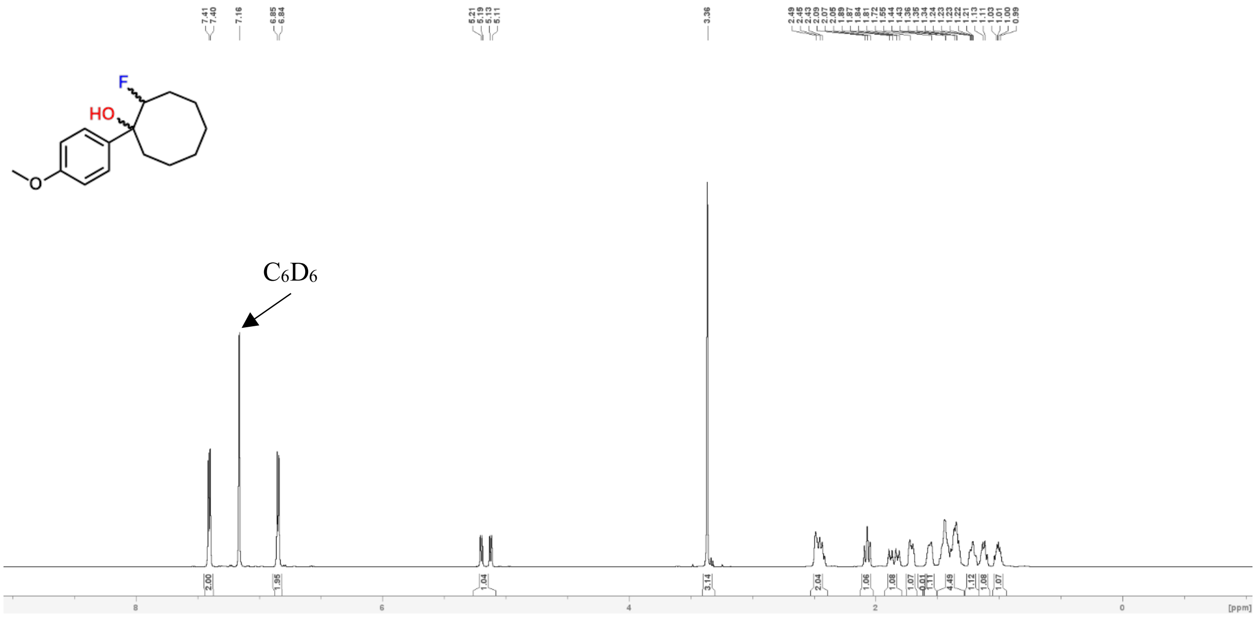

$^{13}C\{^{1}H\}$ NMR (150MHz, C_6D_6) of major isomer 4d

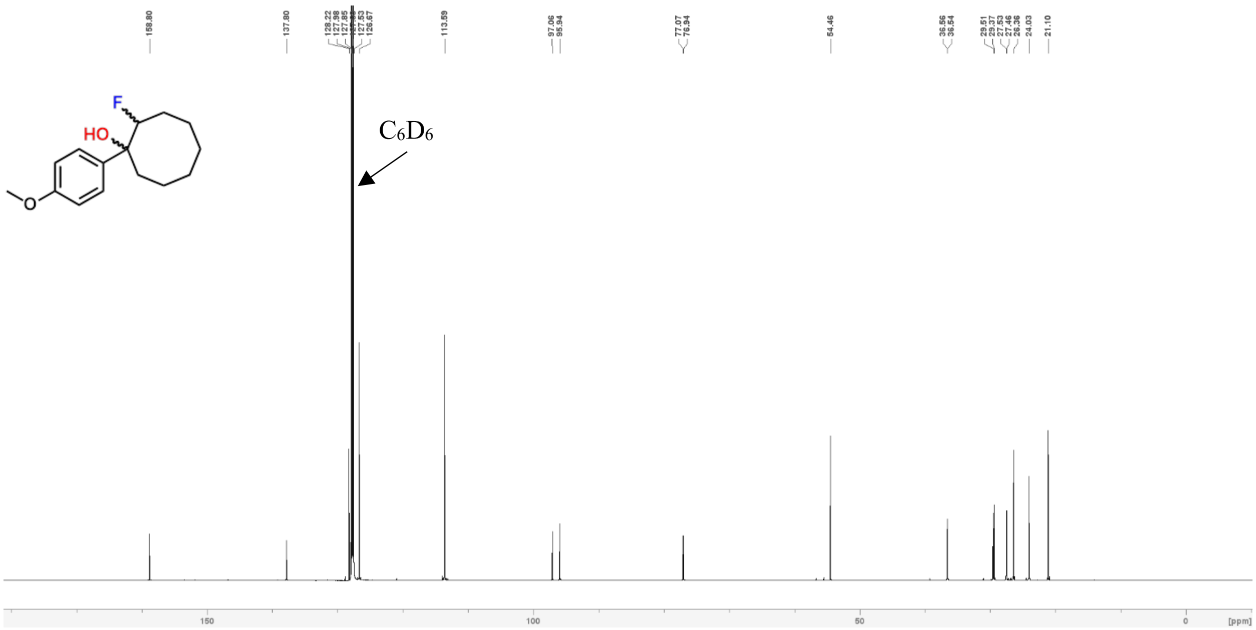

^{19}F NMR (565MHz, C_6D_6) of major isomer 4d

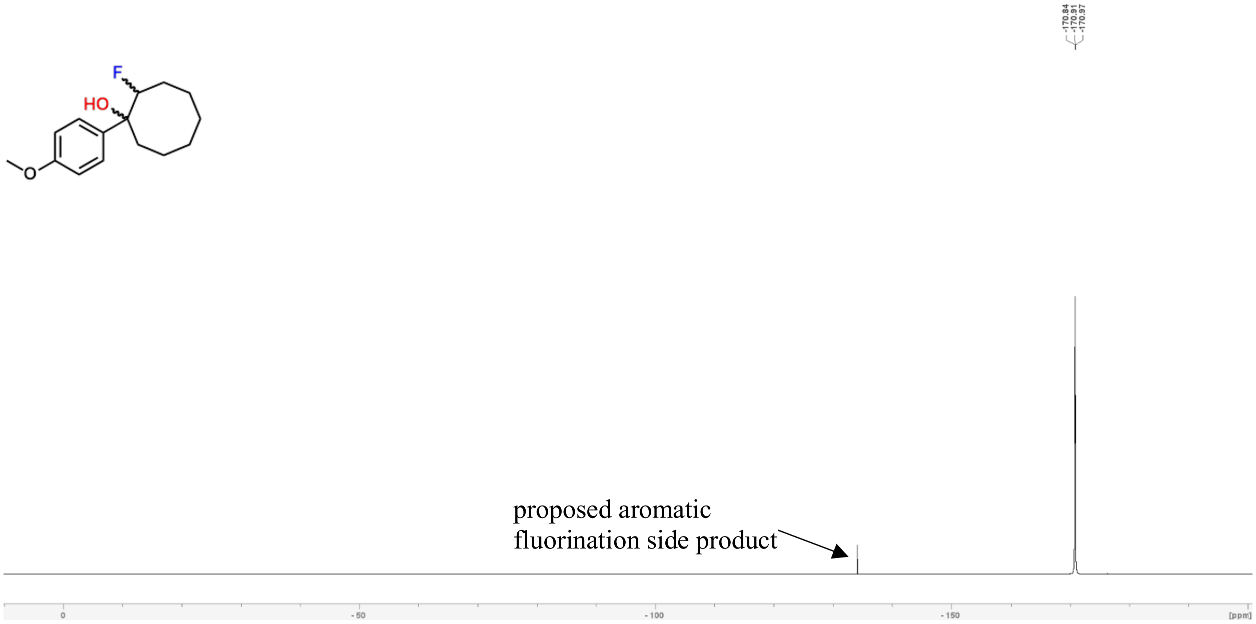

^{1}H NMR (600MHz, C_6D_6) of 5a

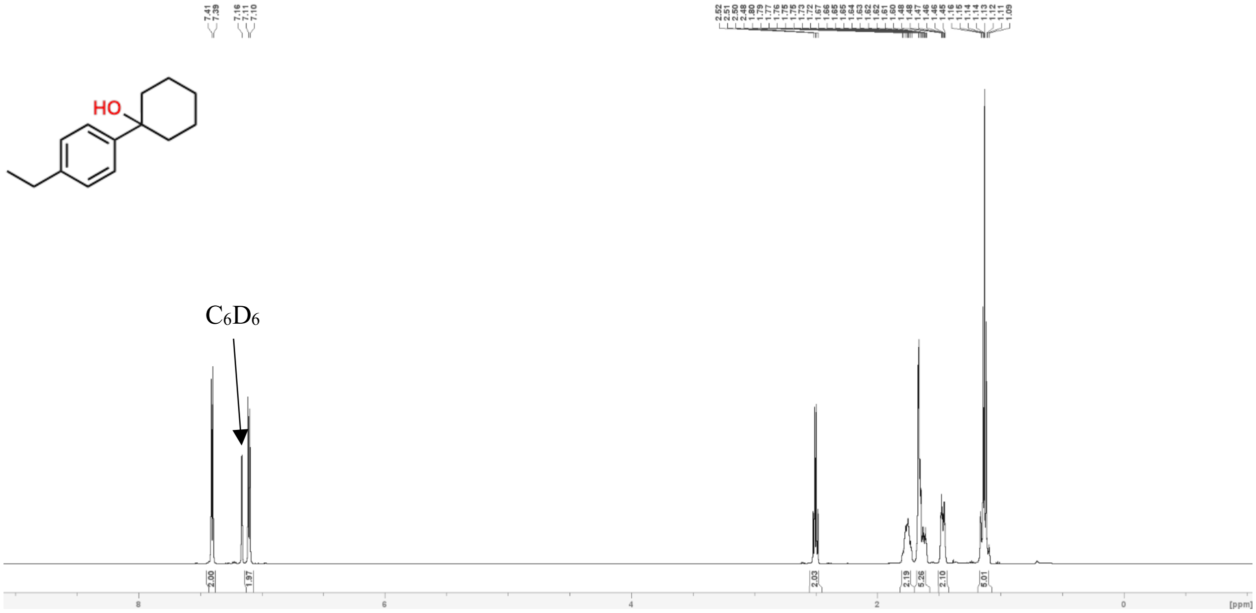

^{13}C{^{1}H} NMR (150MHz, C_6D_6) of 5a

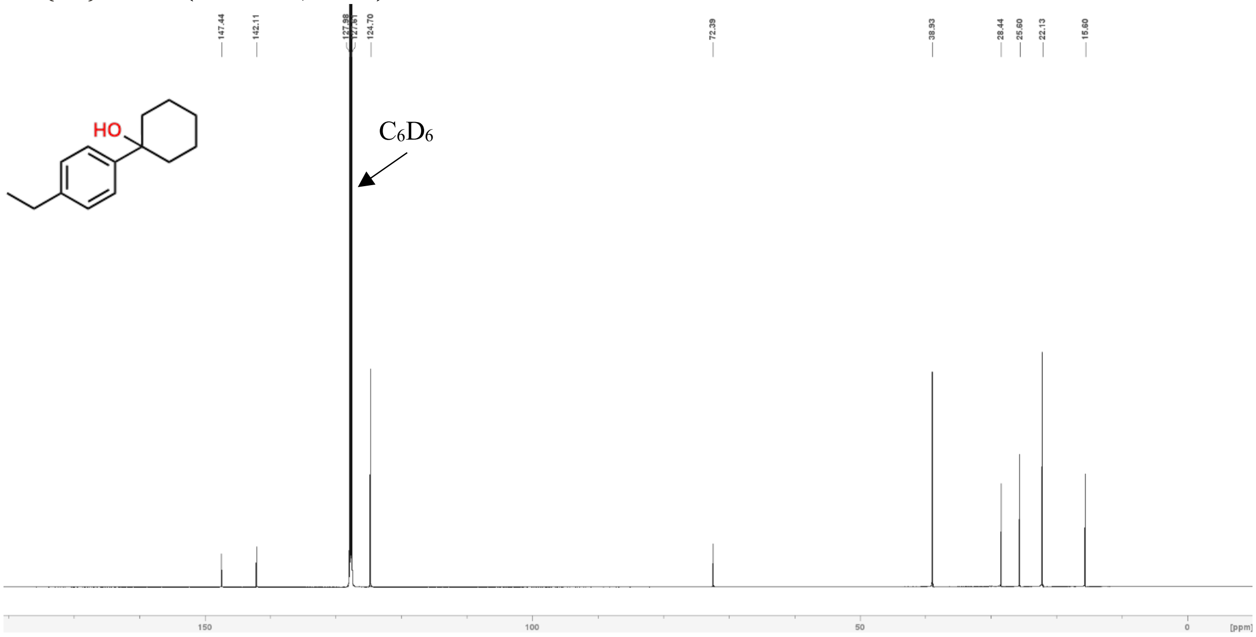

^{1}H NMR (600MHz, C_6D_6) of 5b

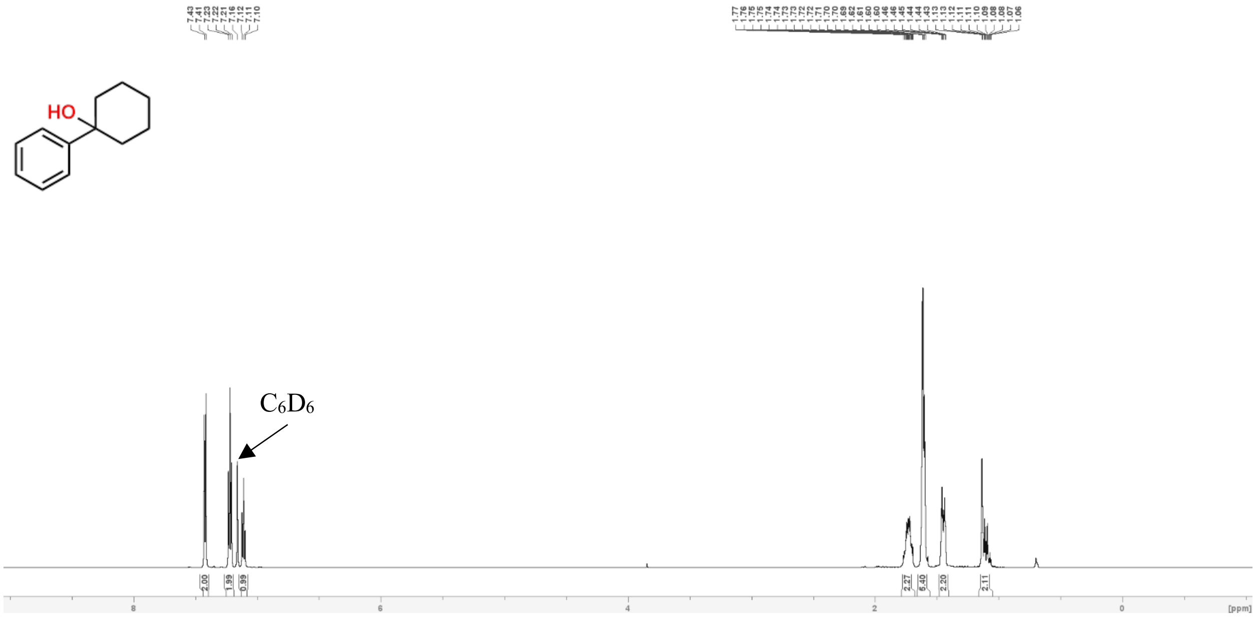

^{13}C{^{1}H} NMR (150MHz, C_6D_6) of 5b

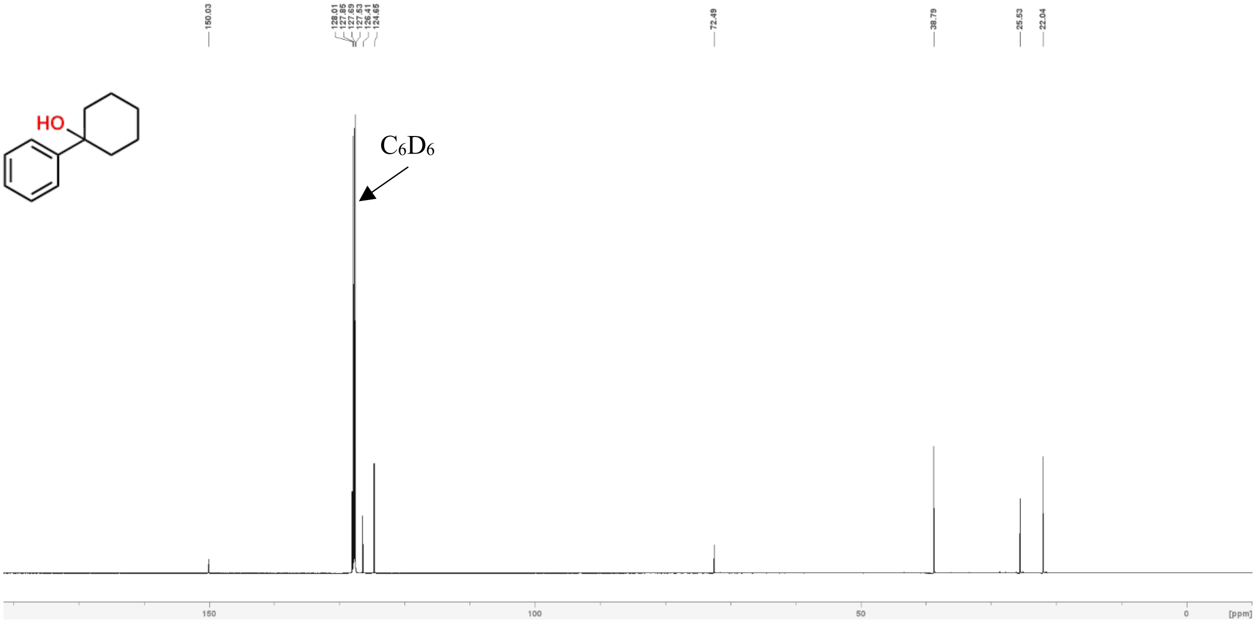

^{1}H NMR (600MHz, C_6D_6) of 5c

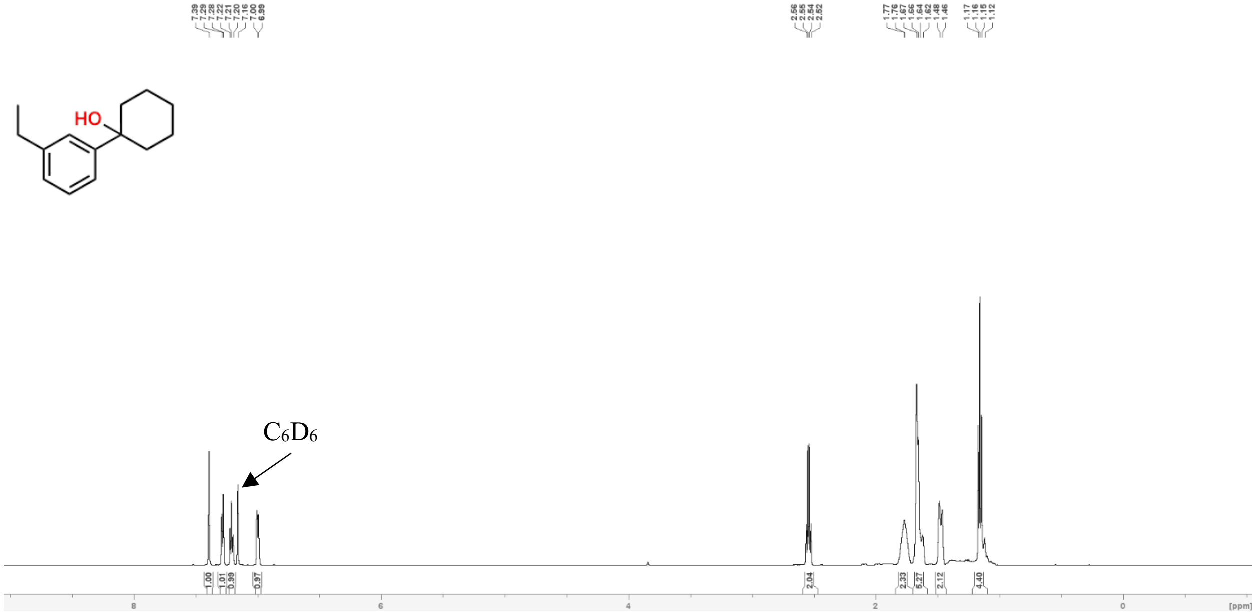

$^{13}C\{^{1}H\}$ NMR (150MHz, C_6D_6) of 5c

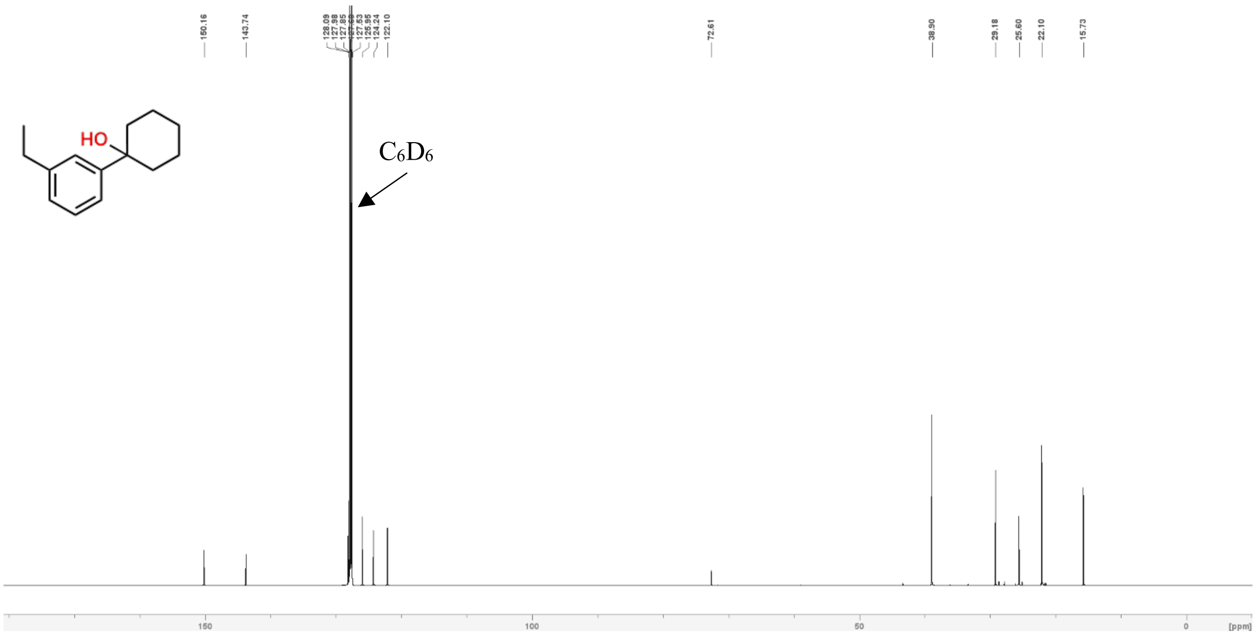

^{1}H NMR (600MHz, C_6D_6) of 6a combined isomers

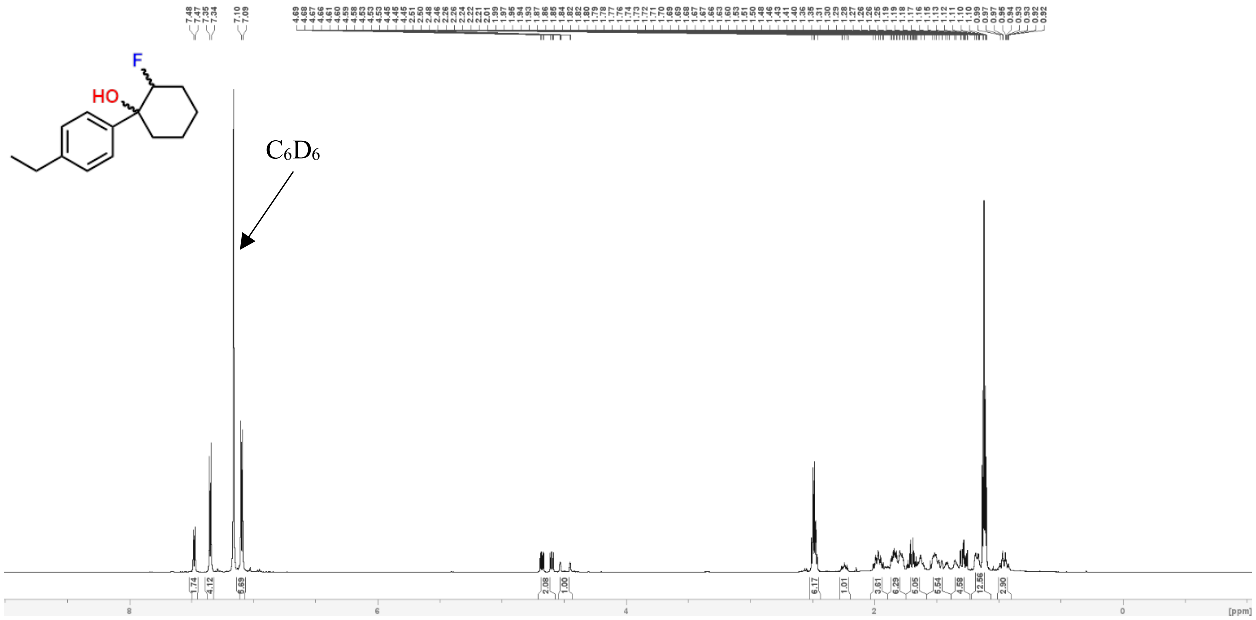

^{1}H NMR (600MHz, C_6D_6) of major isomer 6a

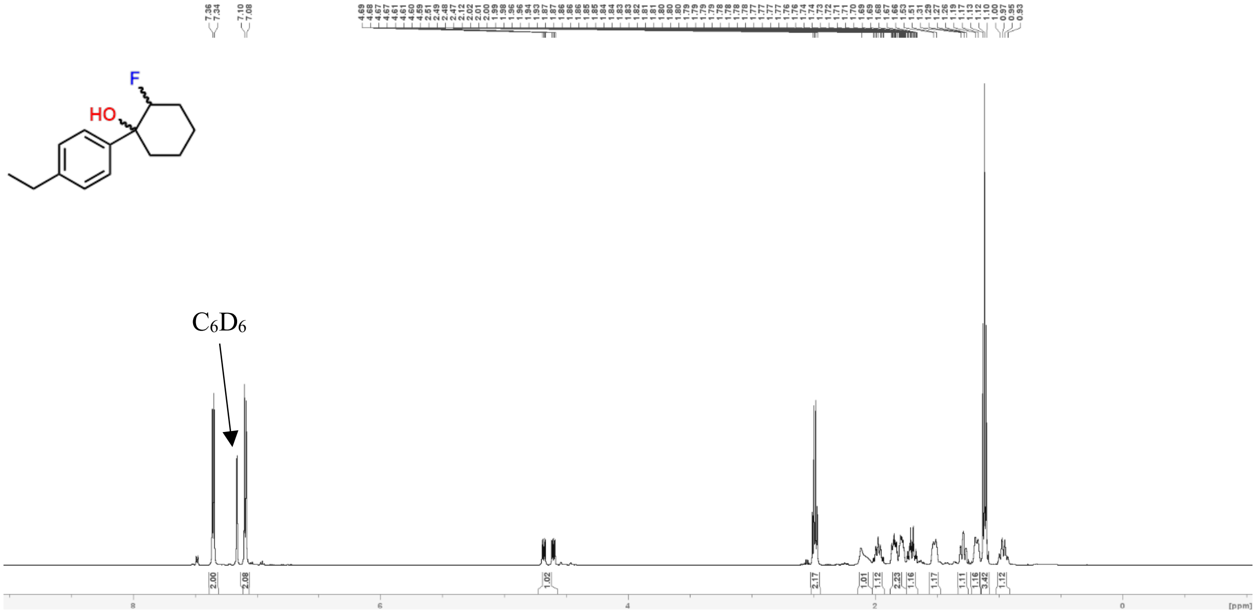

$^{13}C\{^{1}H\}$ NMR (150MHz, C_6D_6) of major isomer 6a

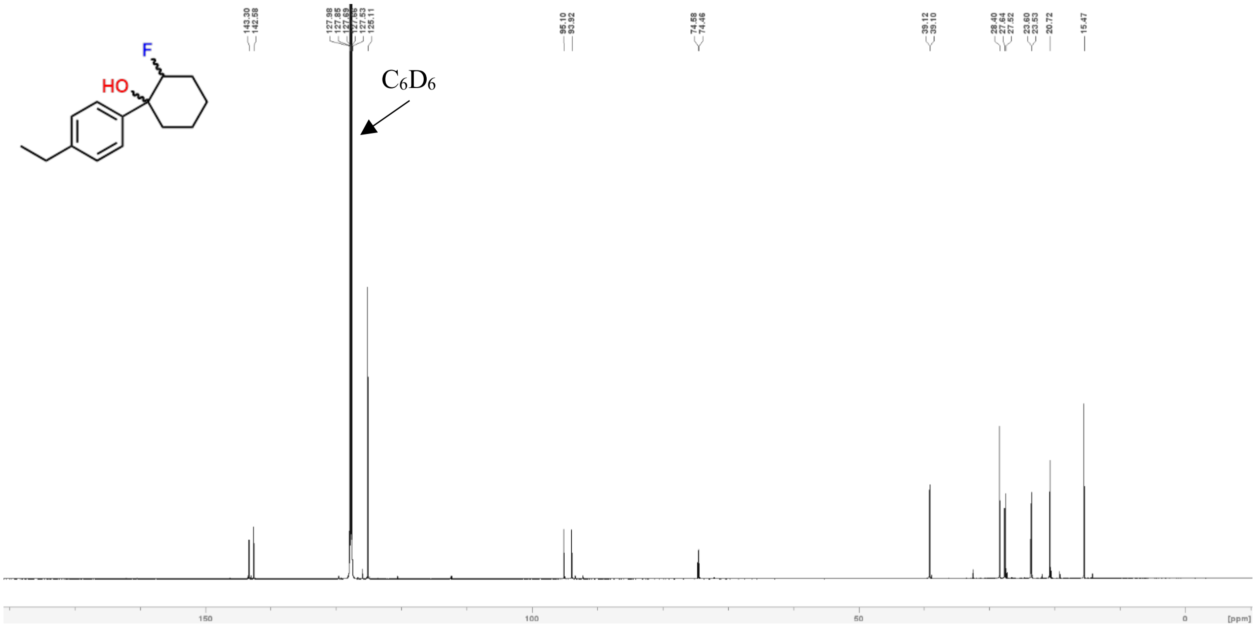

^{19}F NMR (565MHz, C_6D_6) of major isomer 6a

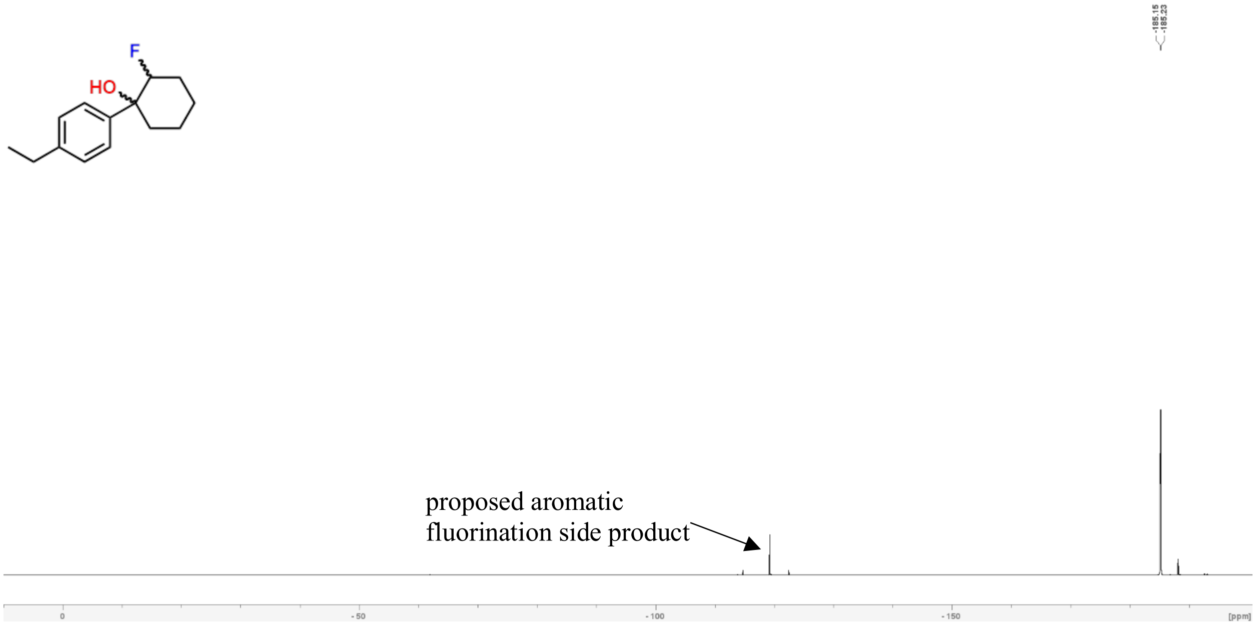

^{1}H NMR (600MHz, C_6D_6) of 6b combined isomers

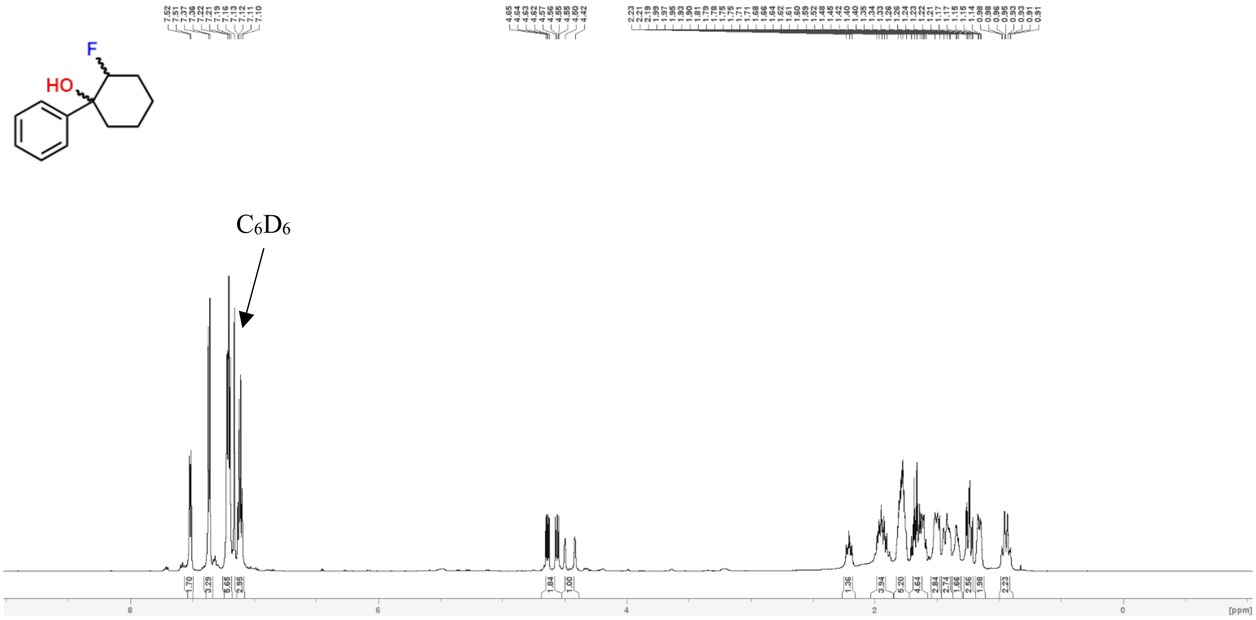

^{1}H NMR (600MHz, C_6D_6) of major isomer 6b

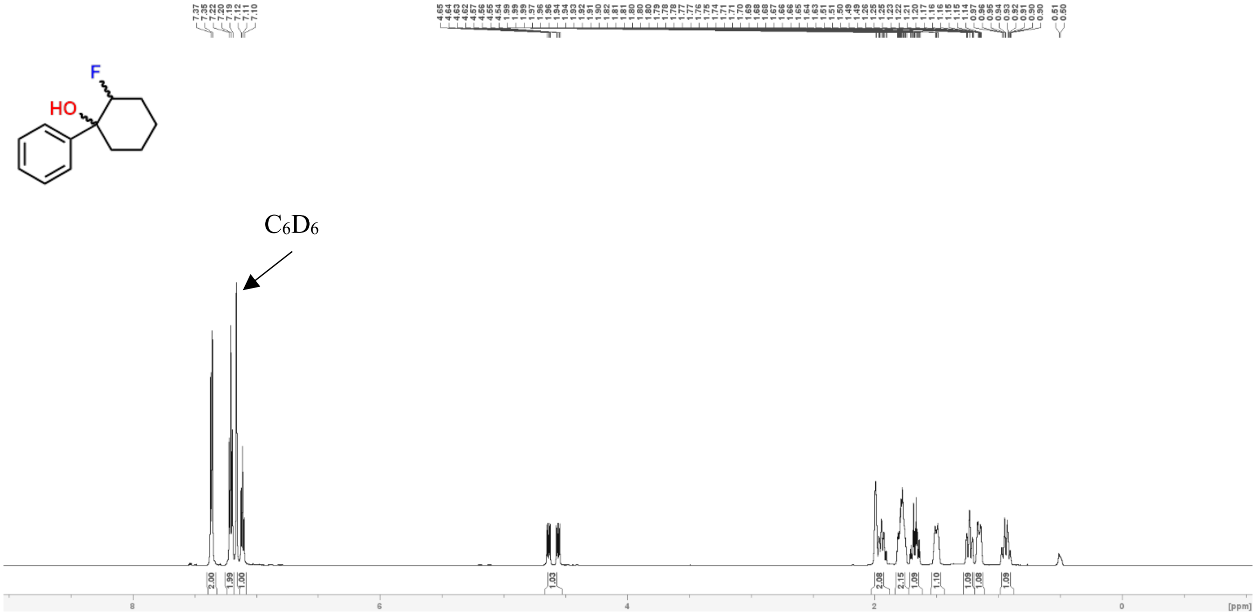

$^{13}C\{^{1}H\}$ NMR (150MHz, C_6D_6) of major isomer 6b

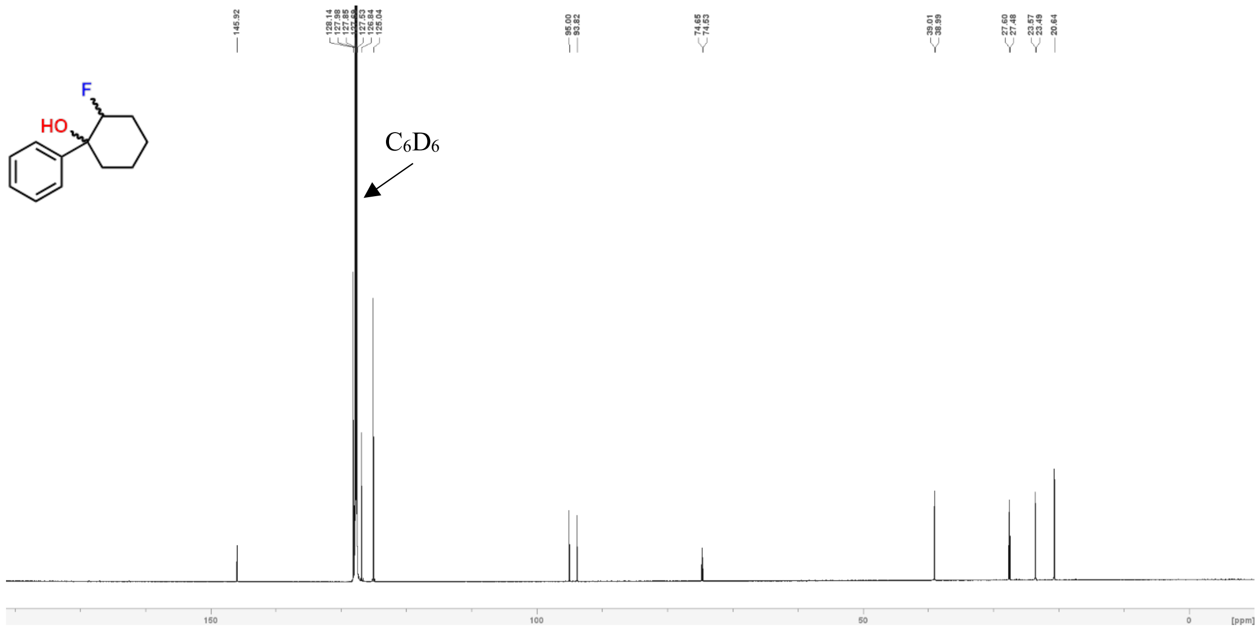

^{19}F NMR (565MHz, C_6D_6) of major isomer 6b

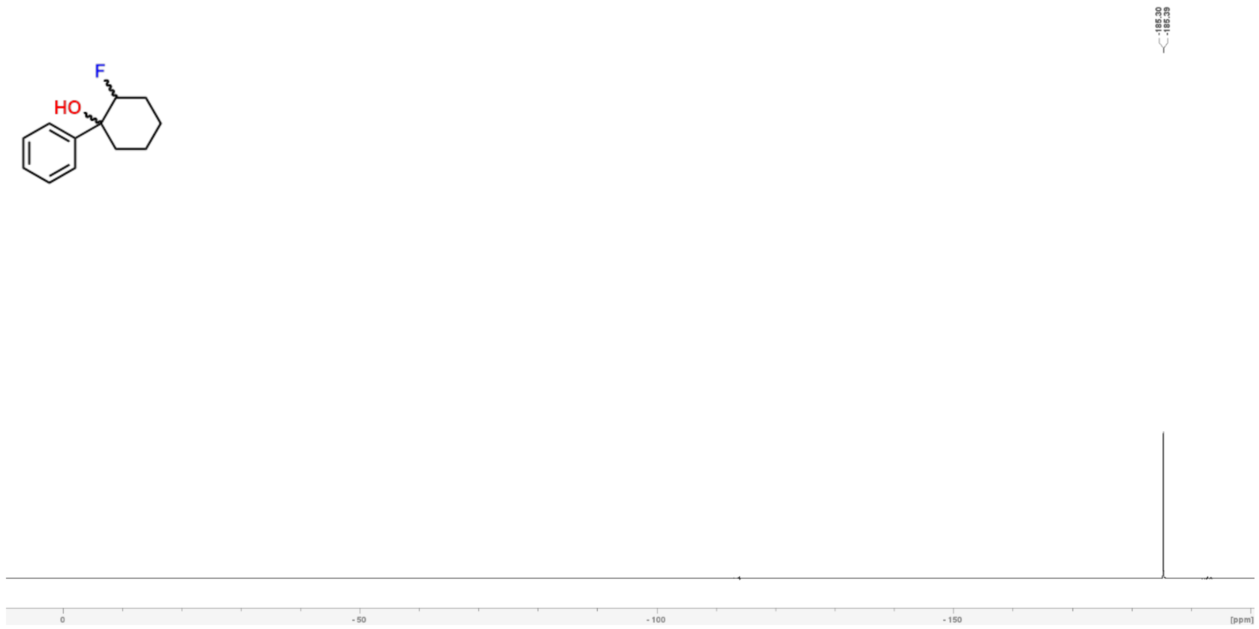

^{1}H NMR (600MHz, C_6D_6) of 6c combined isomers

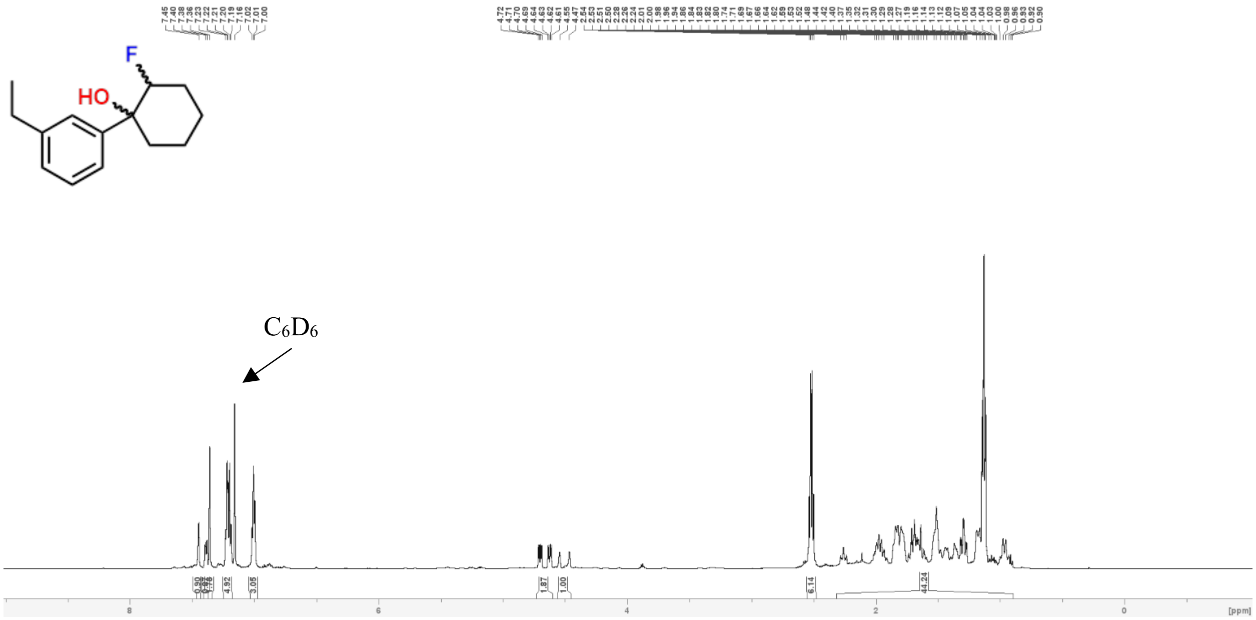

^{1}H NMR (600MHz, C_6D_6) of major isomer 6c

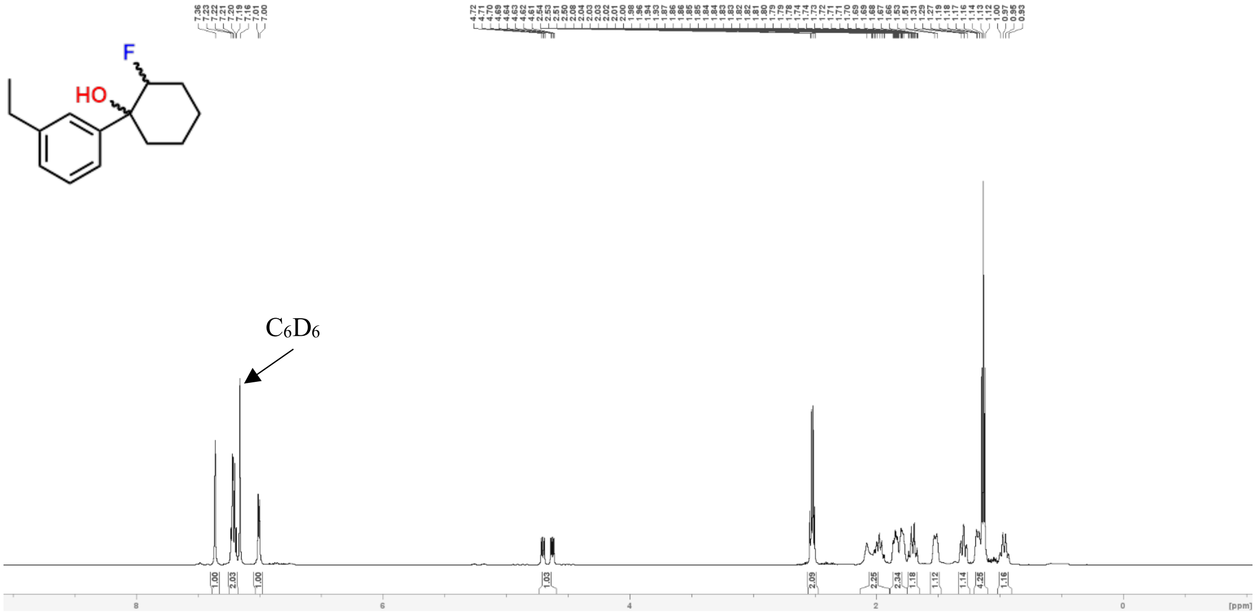

$^{13}C\{^1H\}$ NMR (150MHz, C_6D_6) of major isomer 6c

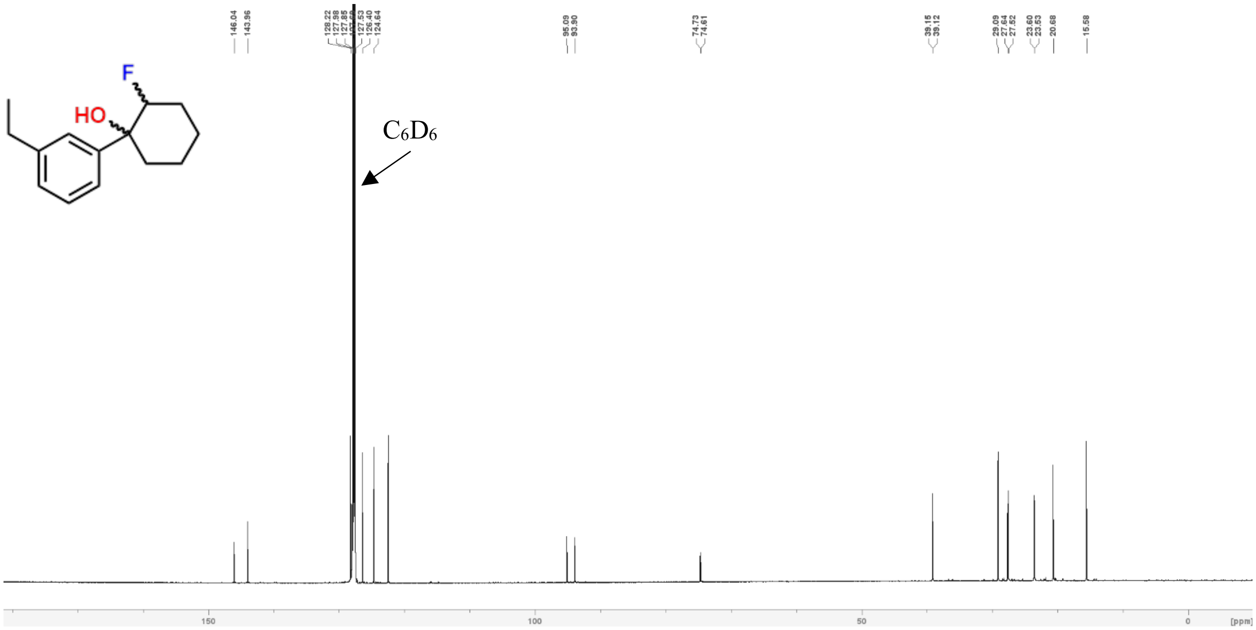

^{19}F NMR (565MHz, C_6D_6) of major isomer 6c

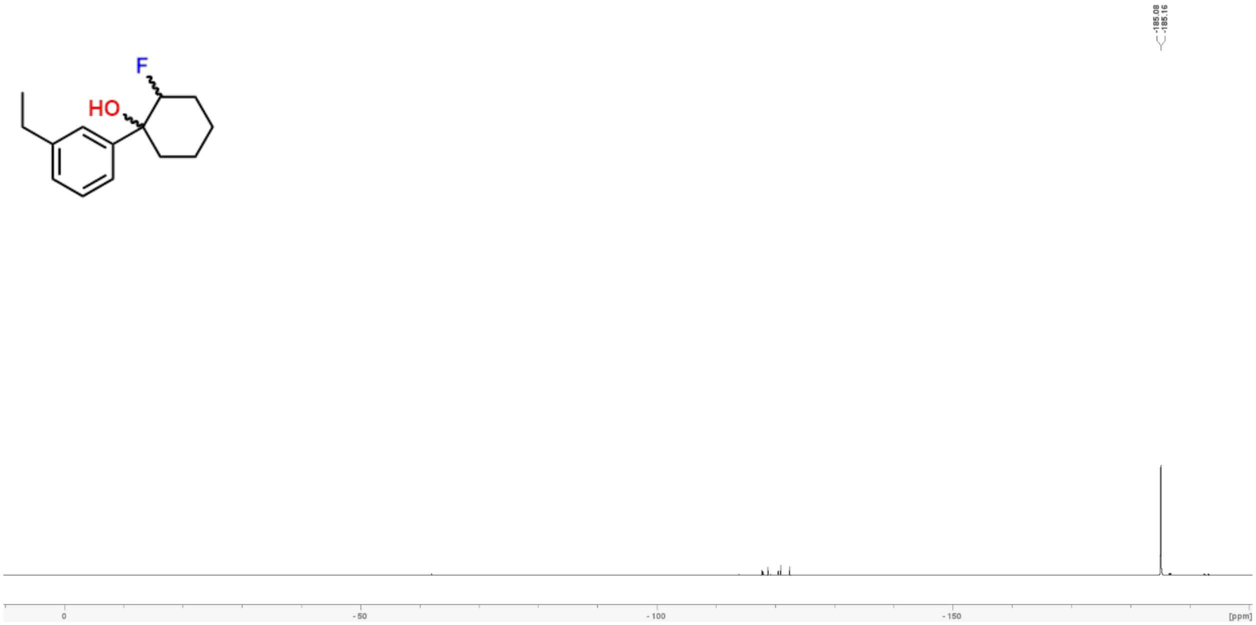

^{1}H NMR (600MHz, C_6D_6) of major isomer 6d

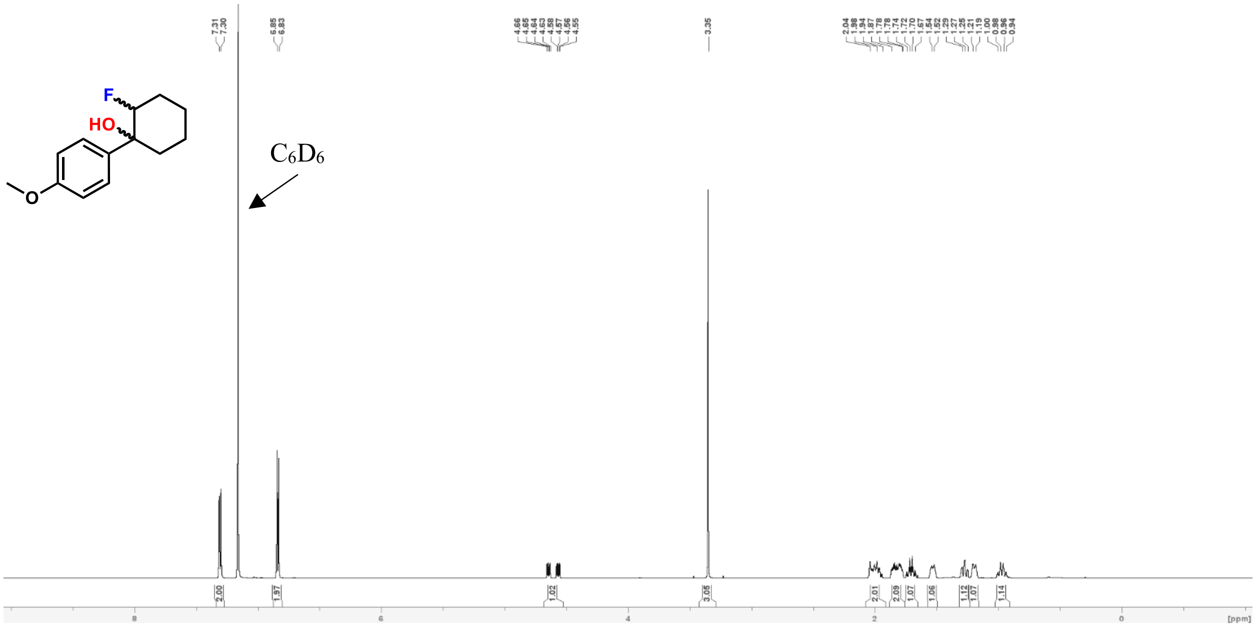

^{13}C{^{1}H} NMR (150MHz, C_6D_6) of major isomer 6d

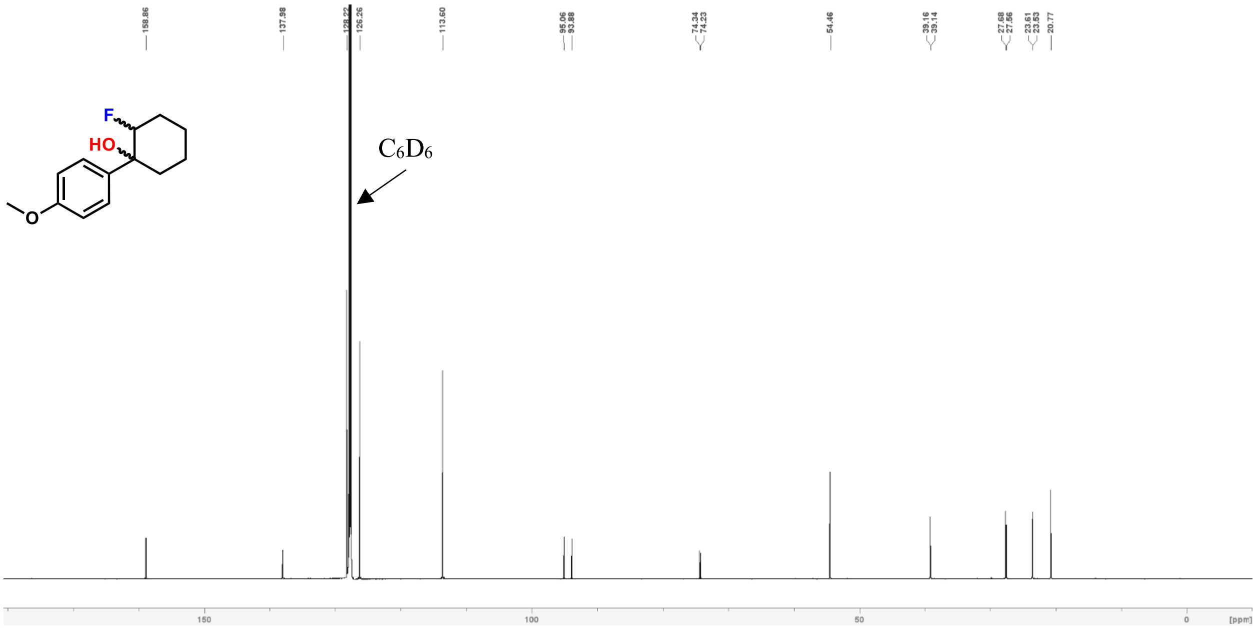

^{19}F NMR (565MHz, $CDCl_3$) of major isomer 6d

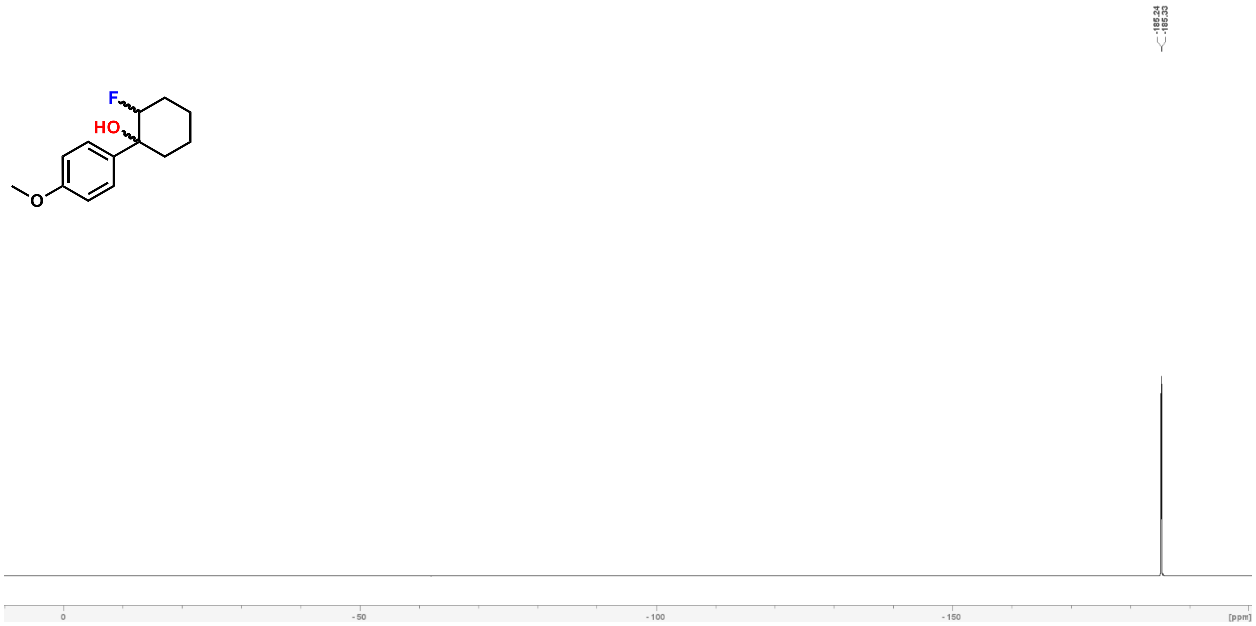

^{1}H NMR (600MHz, C_6D_6) of 7a

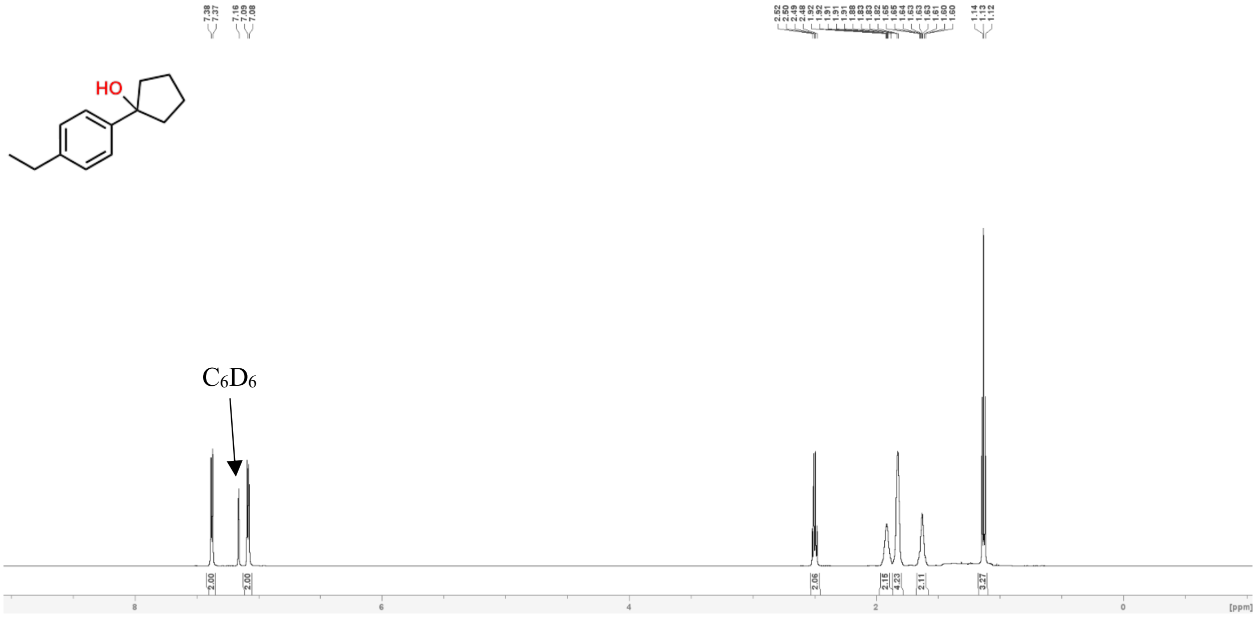

$^{13}C\{^{1}H\}$ NMR (150MHz, C_6D_6) of 7a

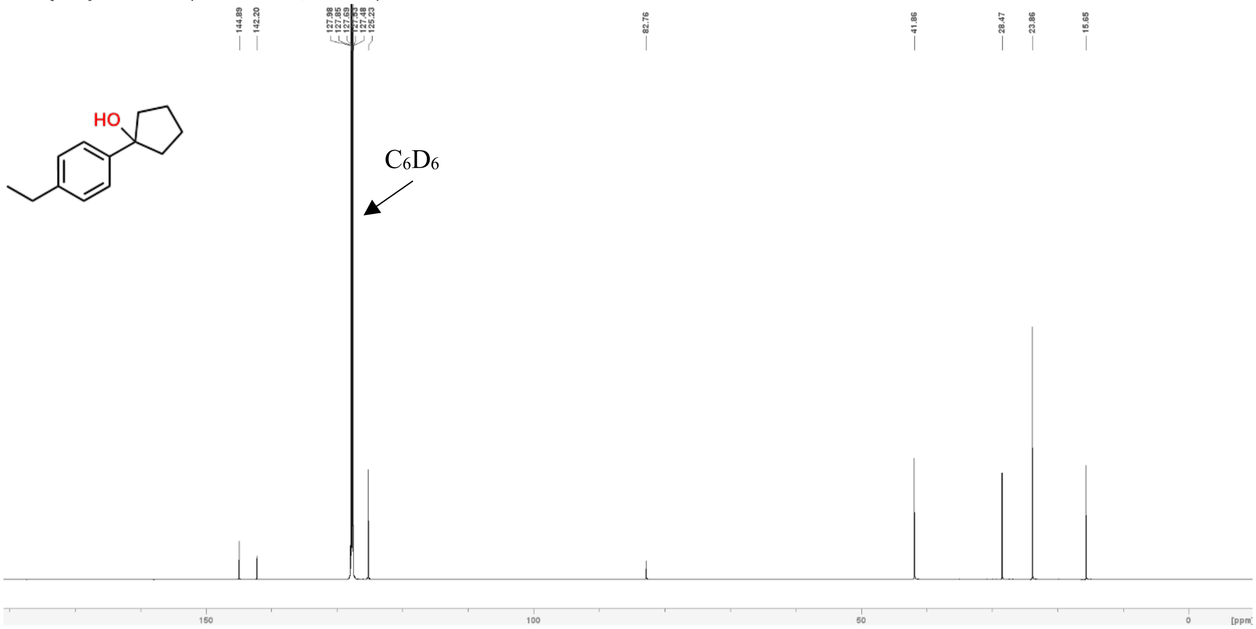

^{1}H NMR (600MHz, C_6D_6) of 7b

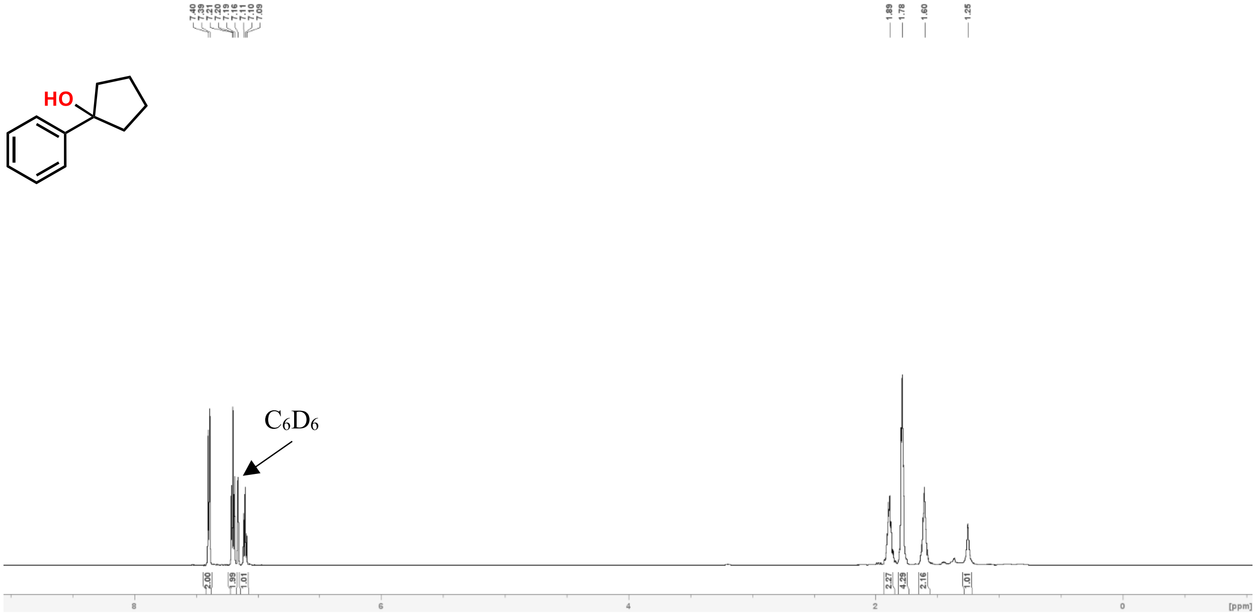

$^{13}C\{^1H\}$ NMR (150MHz, C_6D_6) of 7b

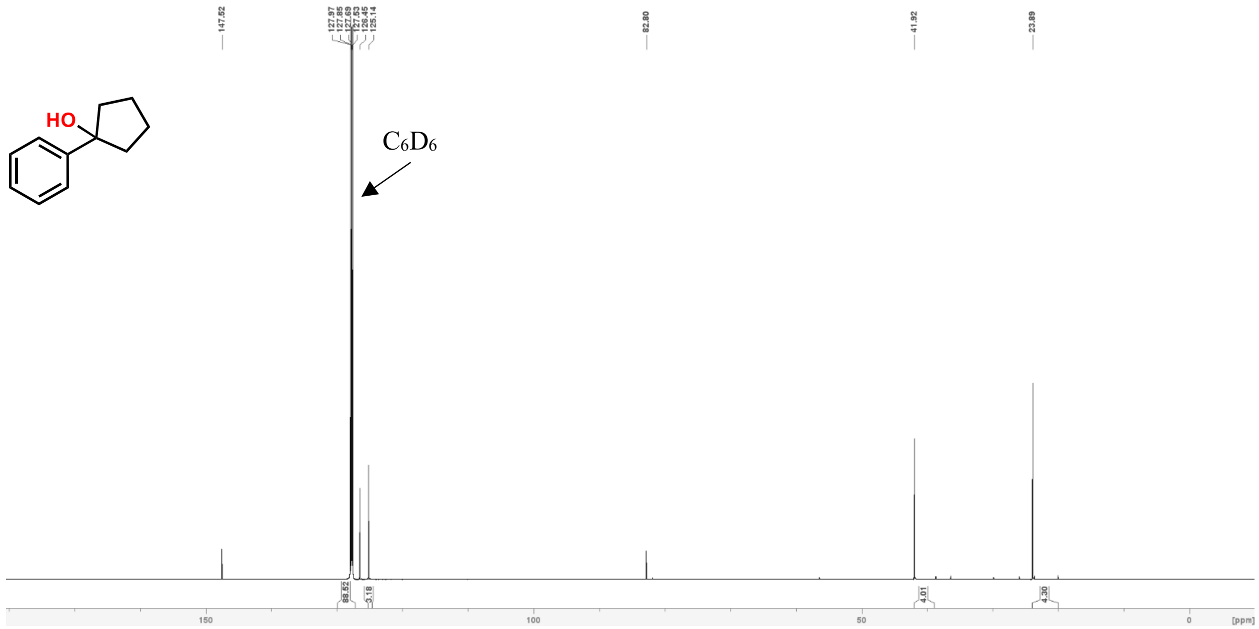

^1H NMR (600MHz, C_6D_6) of 7c

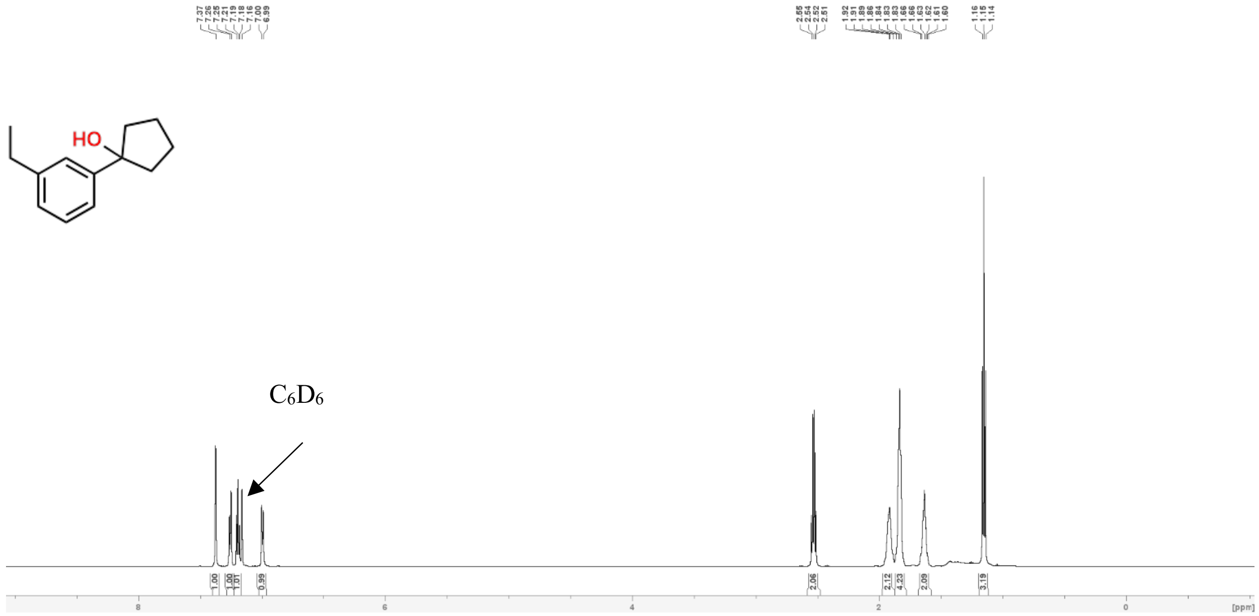

$^{13}C\{^{1}H\}$ NMR (150MHz, C_6D_6) of 7c

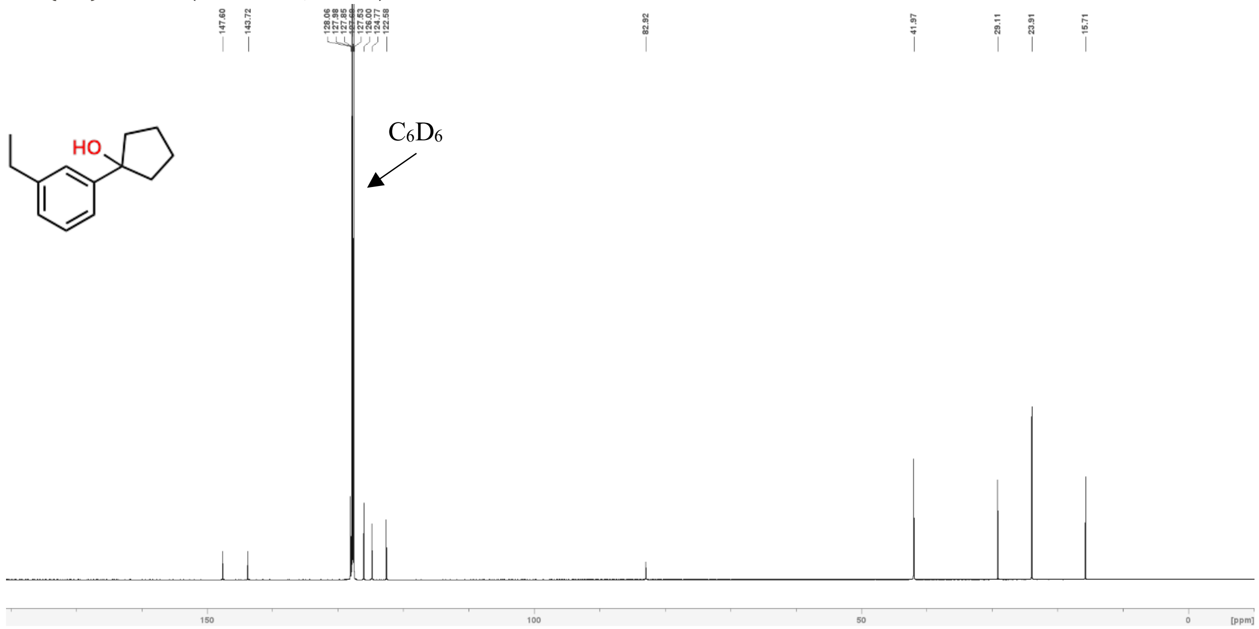

^{1}H NMR (600MHz, C_6D_6) of major isomer 8a

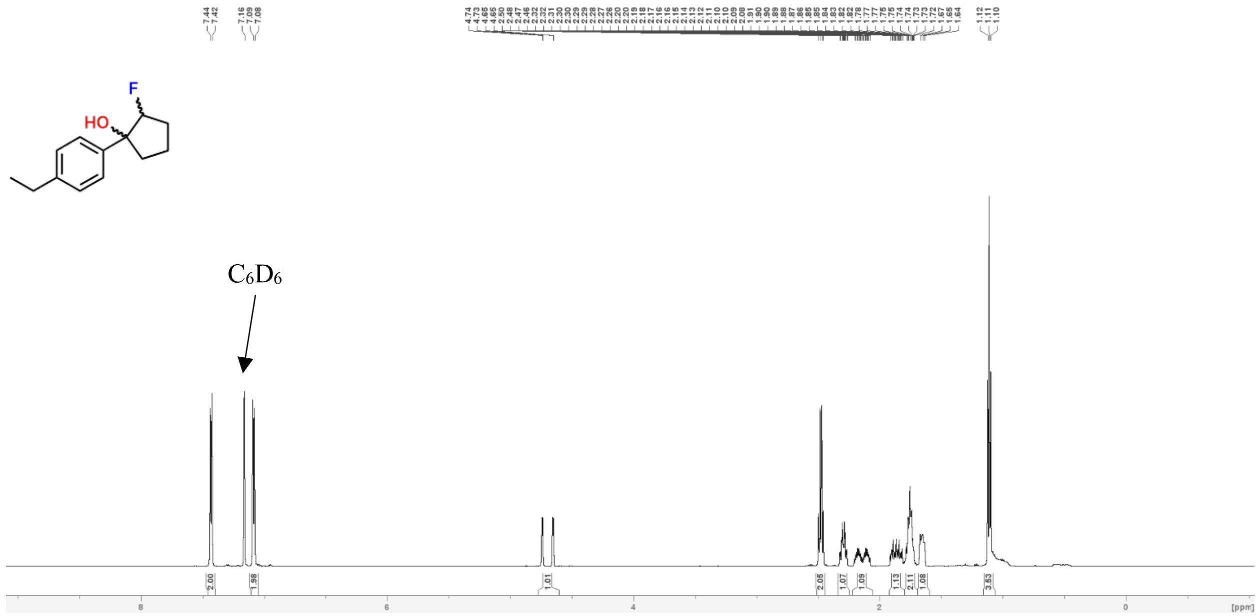

$^{13}C\{^{1}H\}$ NMR (150MHz, C_6D_6) of major isomer **8a**

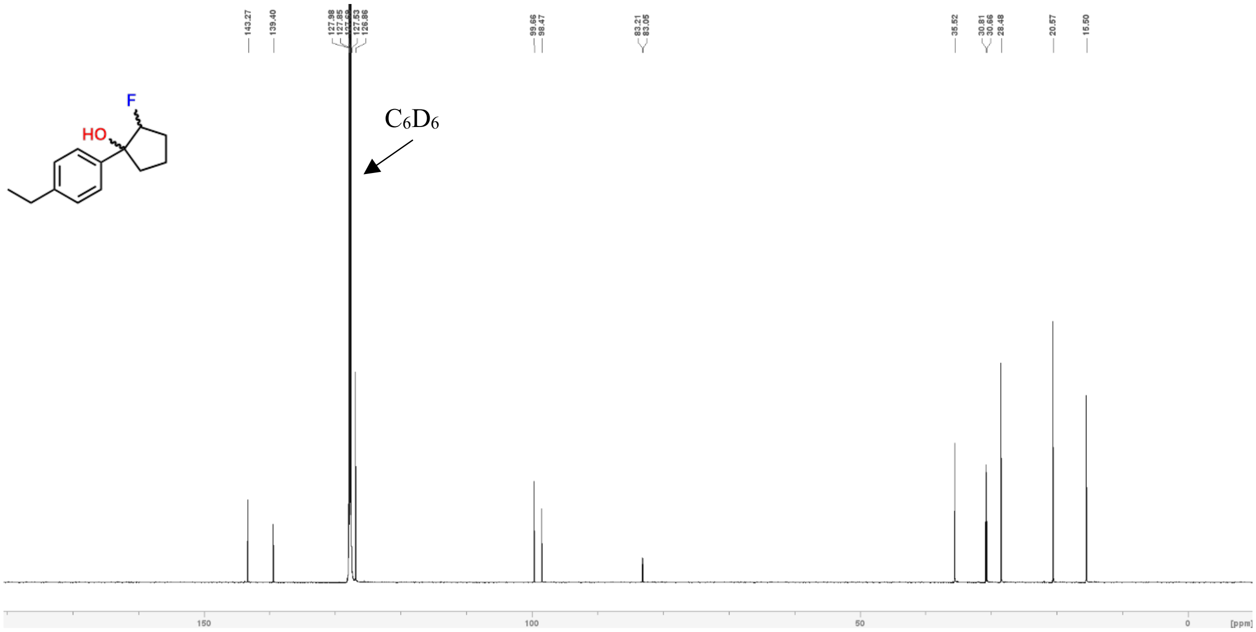

^{19}F NMR (565MHz, C_6D_6) of major isomer **8a**

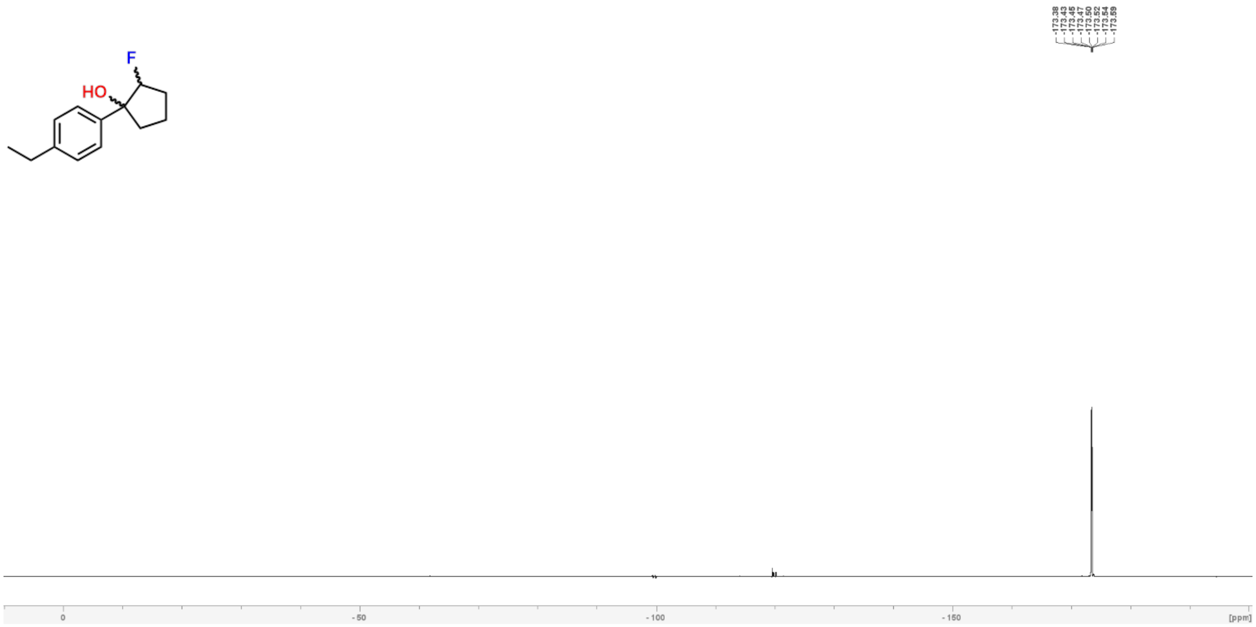

^1H NMR (600MHz, C_6D_6) of major isomer **8b**

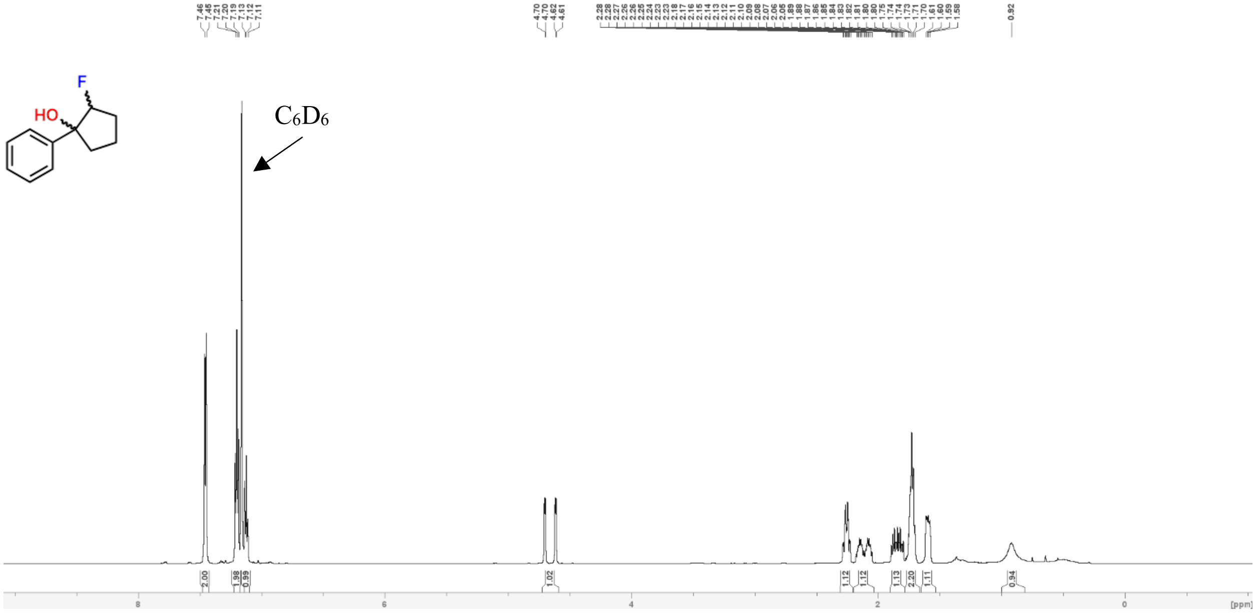

$^{13}C\{^1H\}$ NMR (150MHz, C_6D_6) of major isomer **8b**

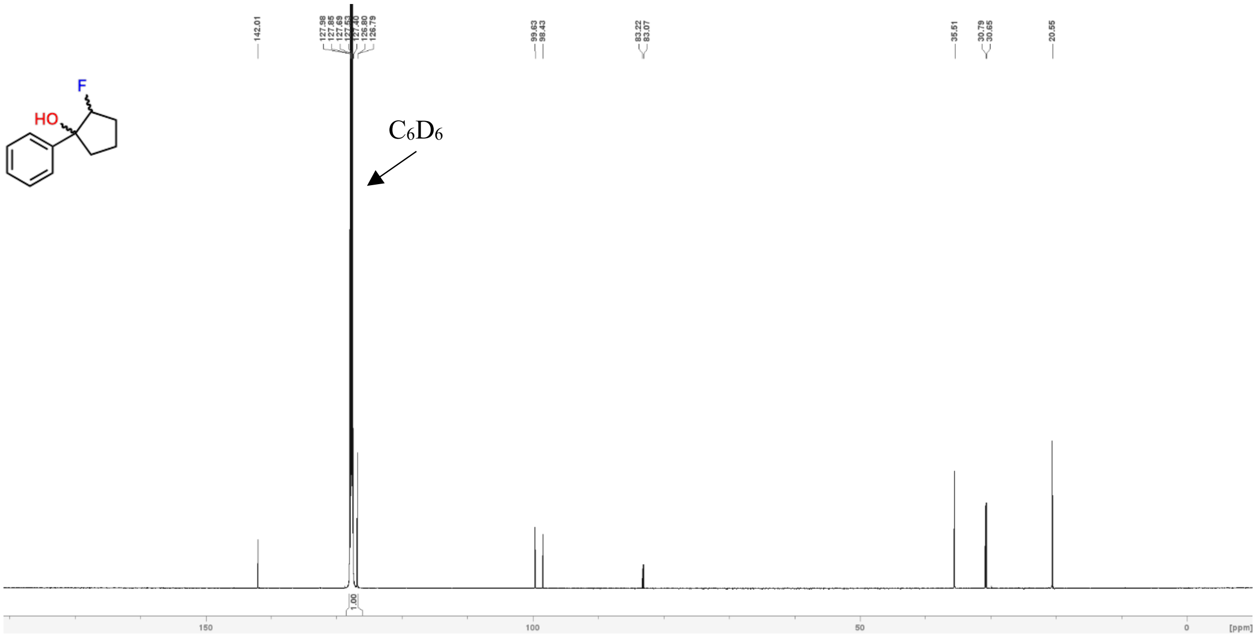

^{19}F NMR (565MHz, C_6D_6) of major isomer 8b

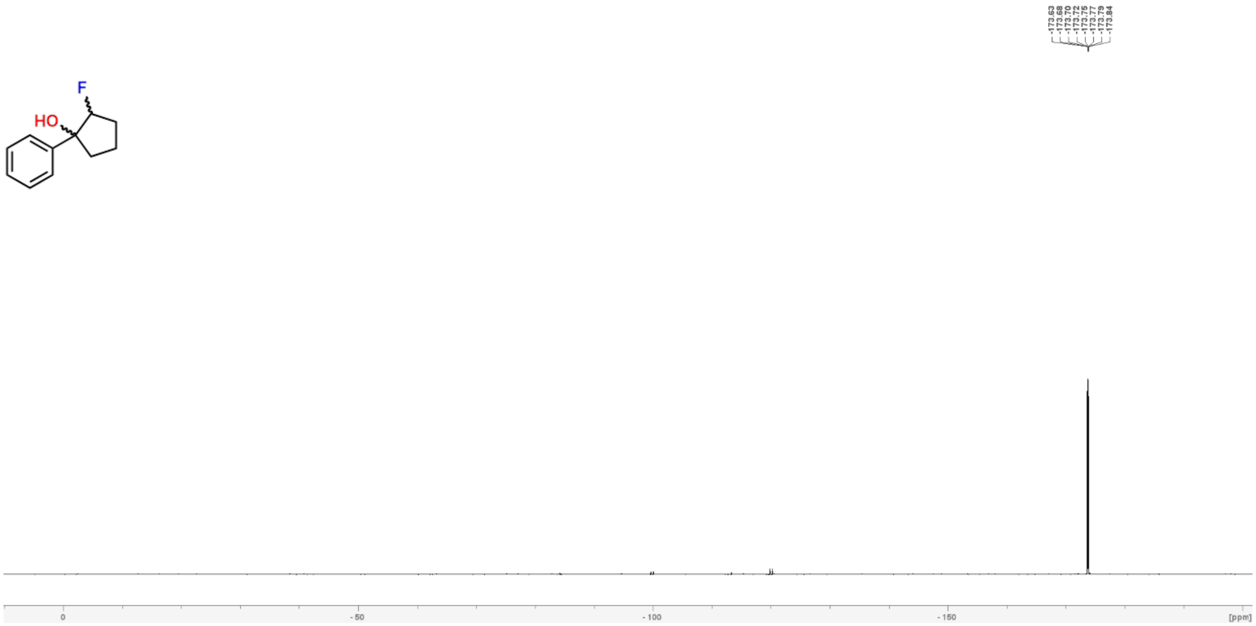

^{1}H NMR (600MHz, C_6D_6) of major isomer 8c

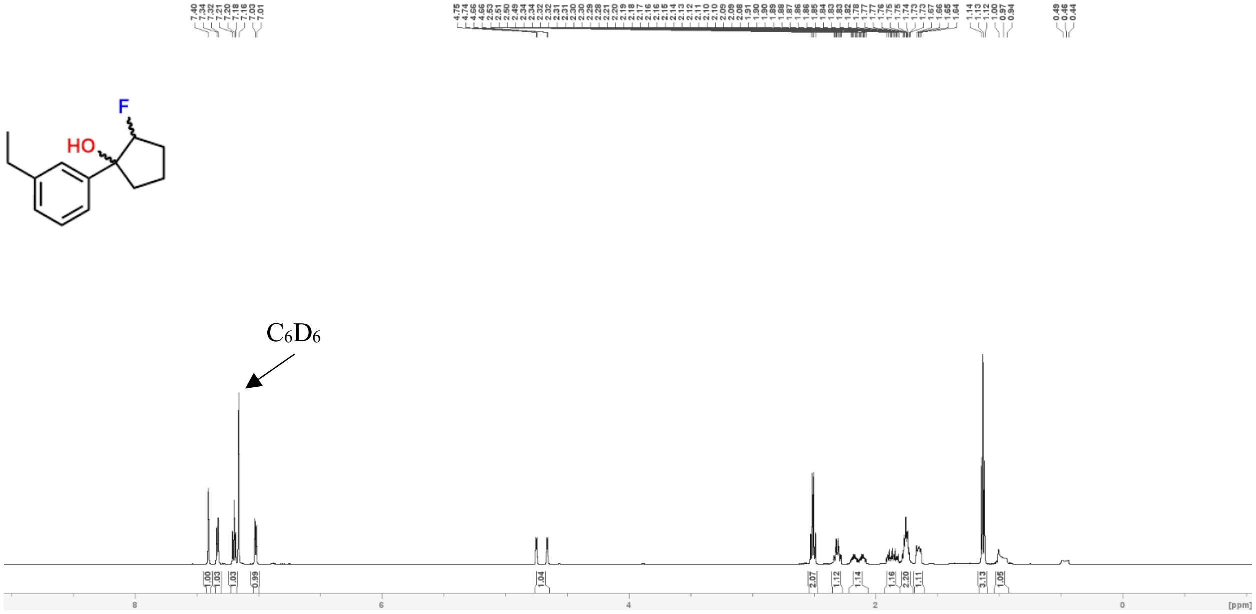

$^{13}C\{^1H\}$ NMR (150MHz, C_6D_6) of major isomer **8c**

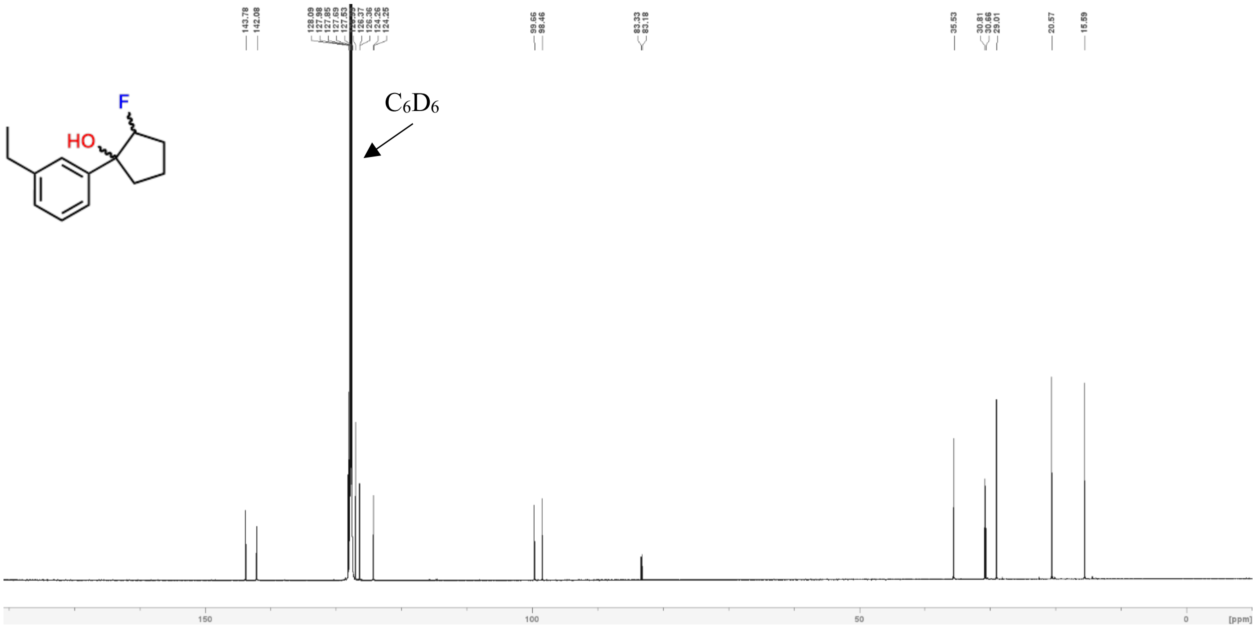

^{19}F NMR (565MHz, C_6D_6) of major isomer **8c**

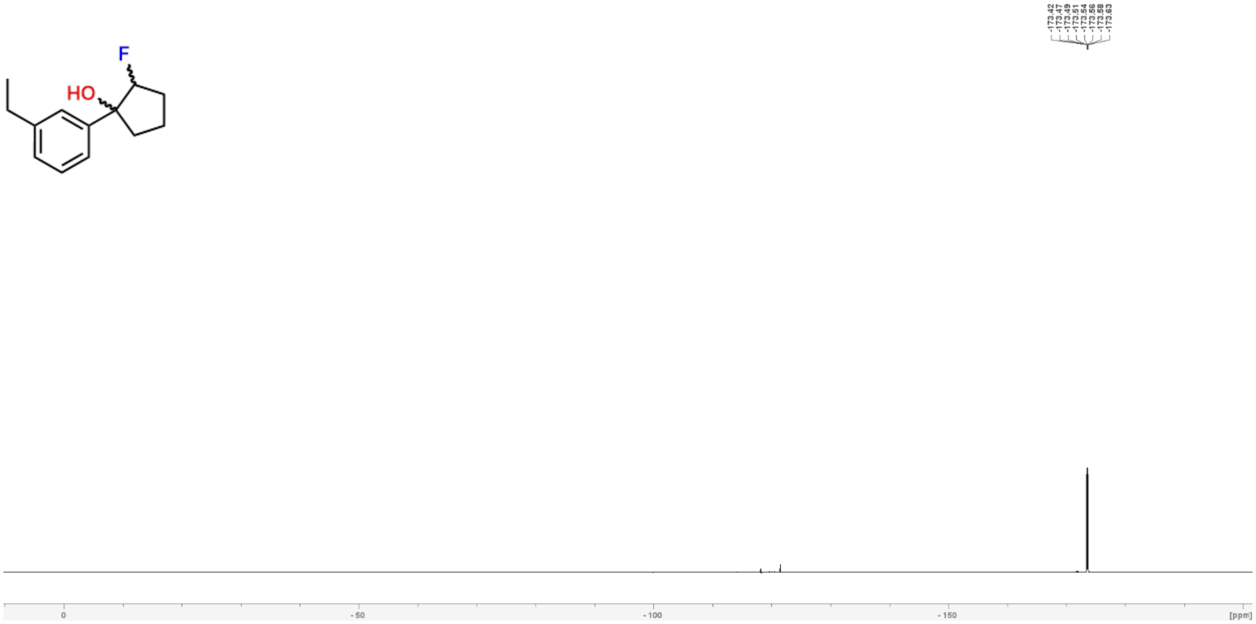

^{1}H NMR (600MHz, $CDCl_3$) of major isomer 8d

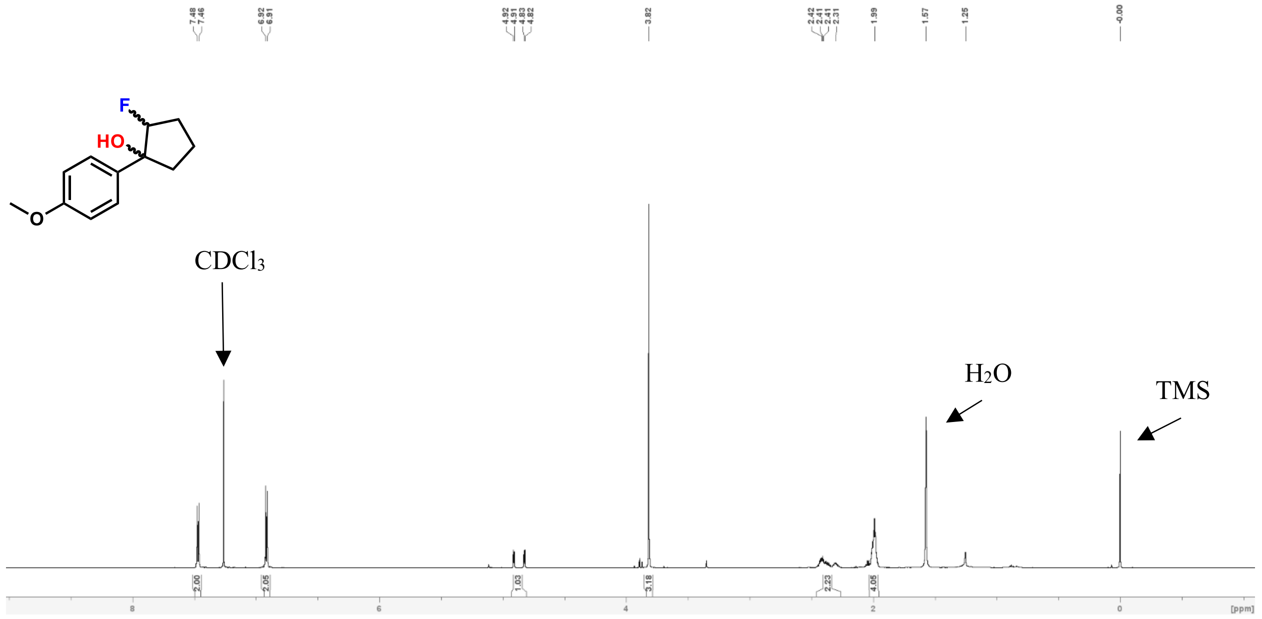

$^{13}C\{^{1}H\}$DEPTQ135 (150MHz, $CDCl_3$) of major isomer 8d

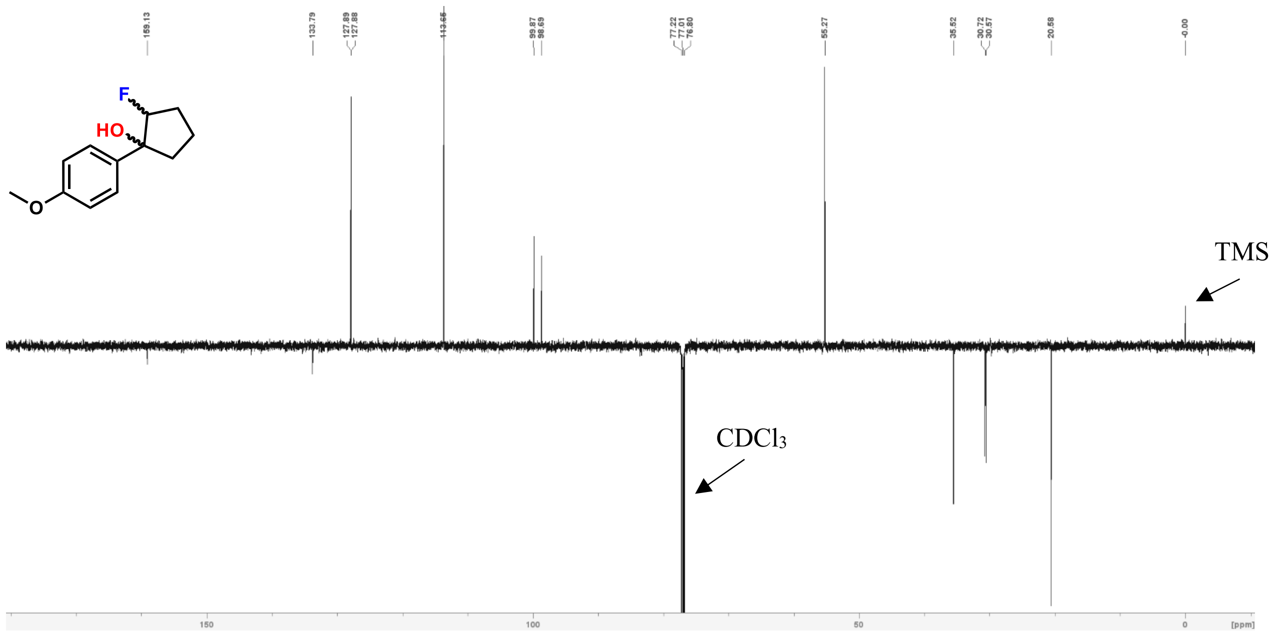

^{19}F NMR (565MHz, $CDCl_3$) of major isomer 8d

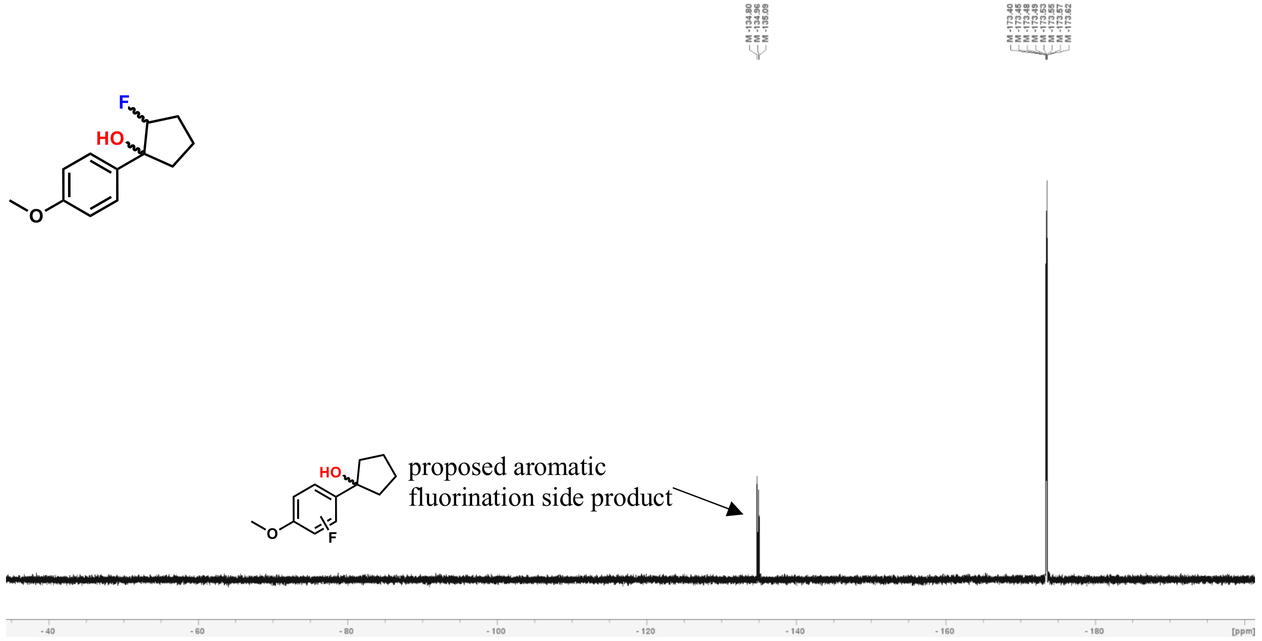

^{1}H NMR (600MHz, C_6D_6) of 10a combined isomers

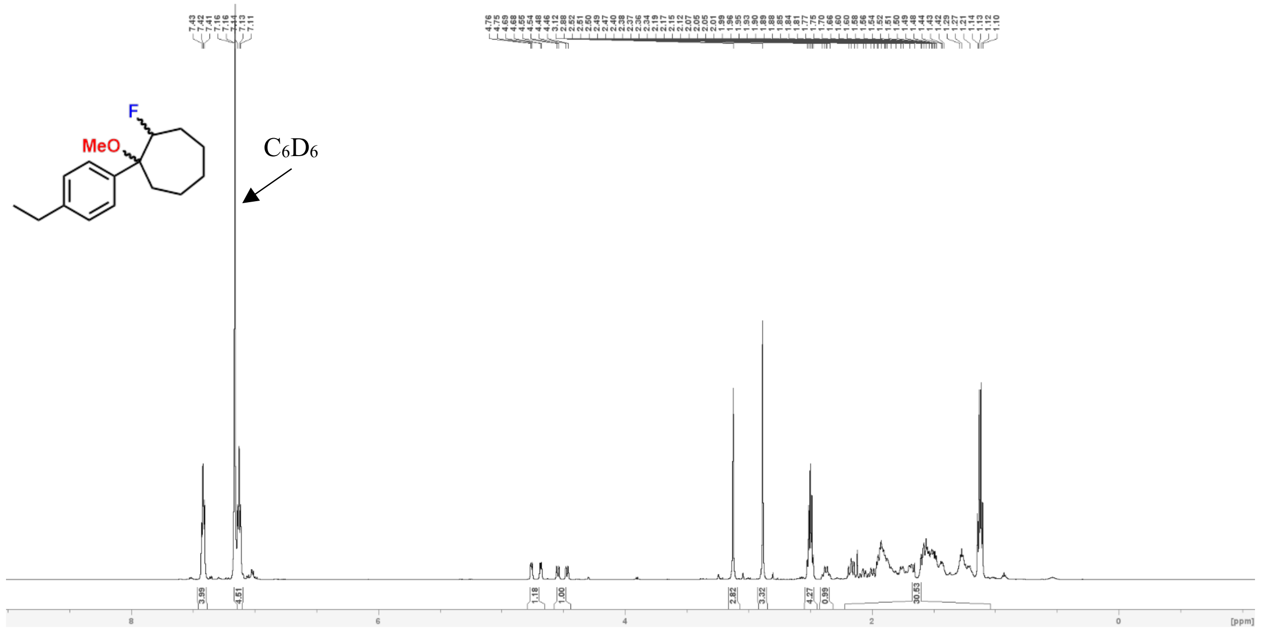

^{1}H NMR (600MHz, C_6D_6) of major isomer 10aa

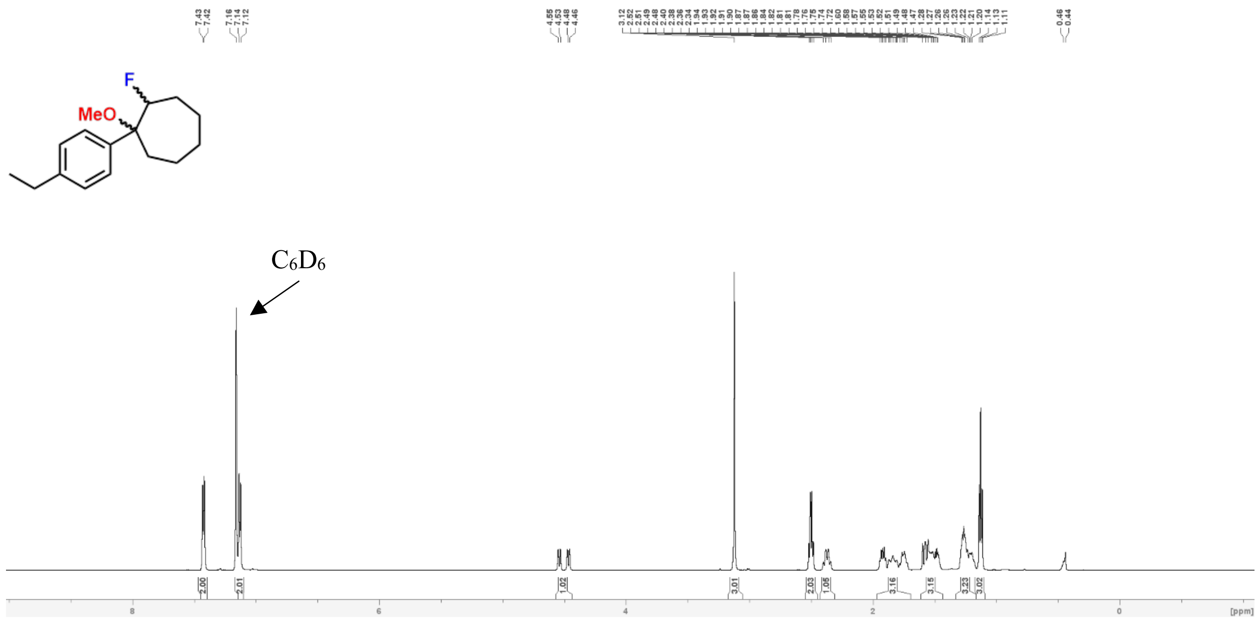

^{13}C{^{1}H} NMR (150MHz, C_6D_6) of major isomer 10aa

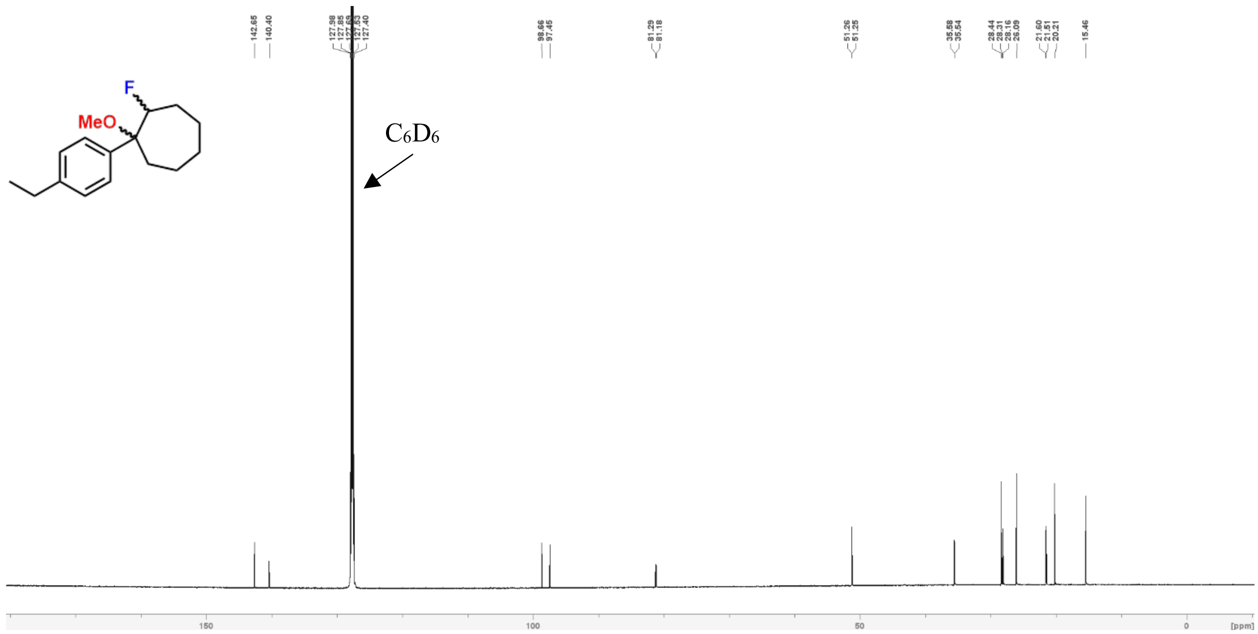

^{19}F NMR (565MHz, C_6D_6) of major isomer 10aa

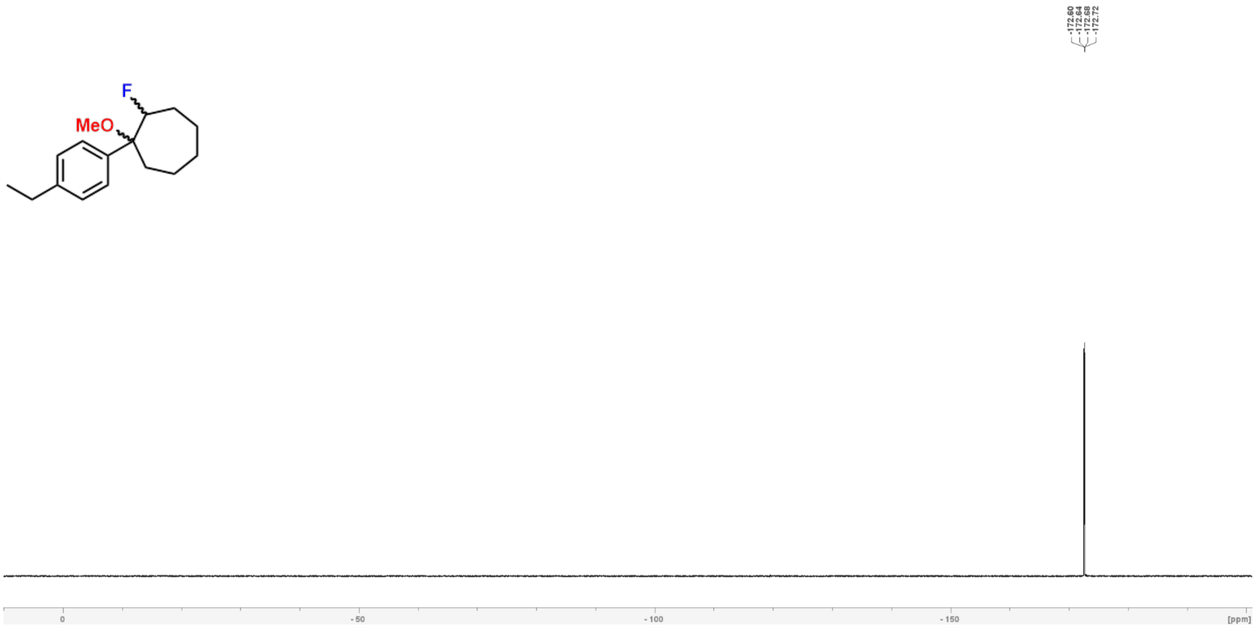

^{1}H NMR (600MHz, C_6D_6) of minor isomer 10ab

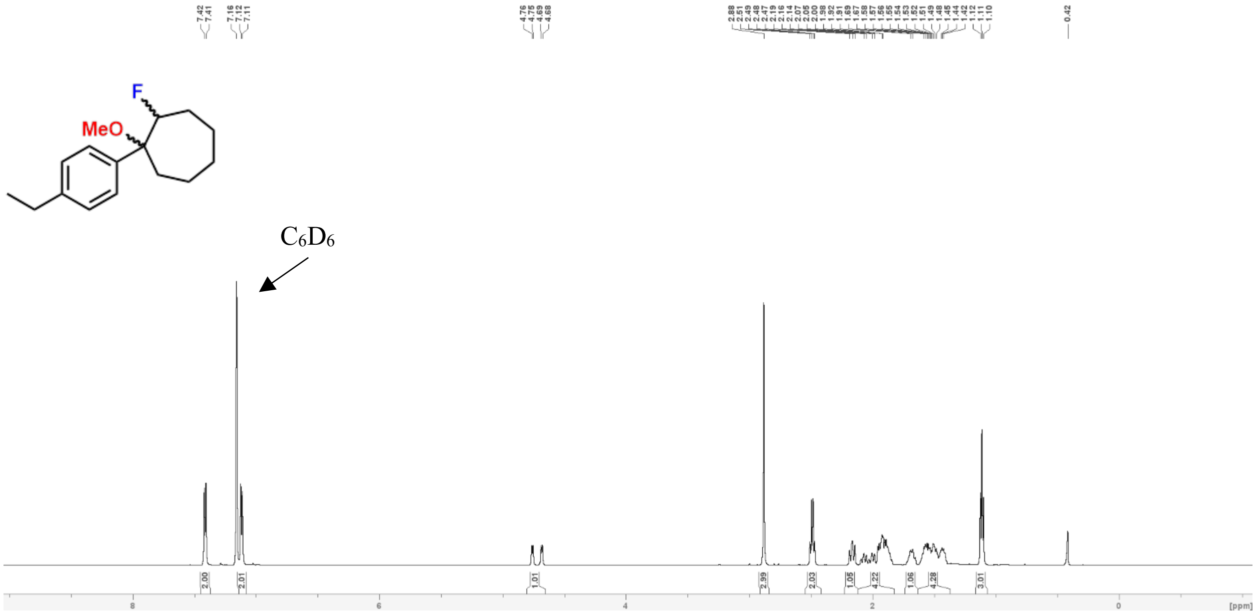

$^{13}C\{^{1}H\}$ NMR (150MHz, C_6D_6) of minor isomer 10ab

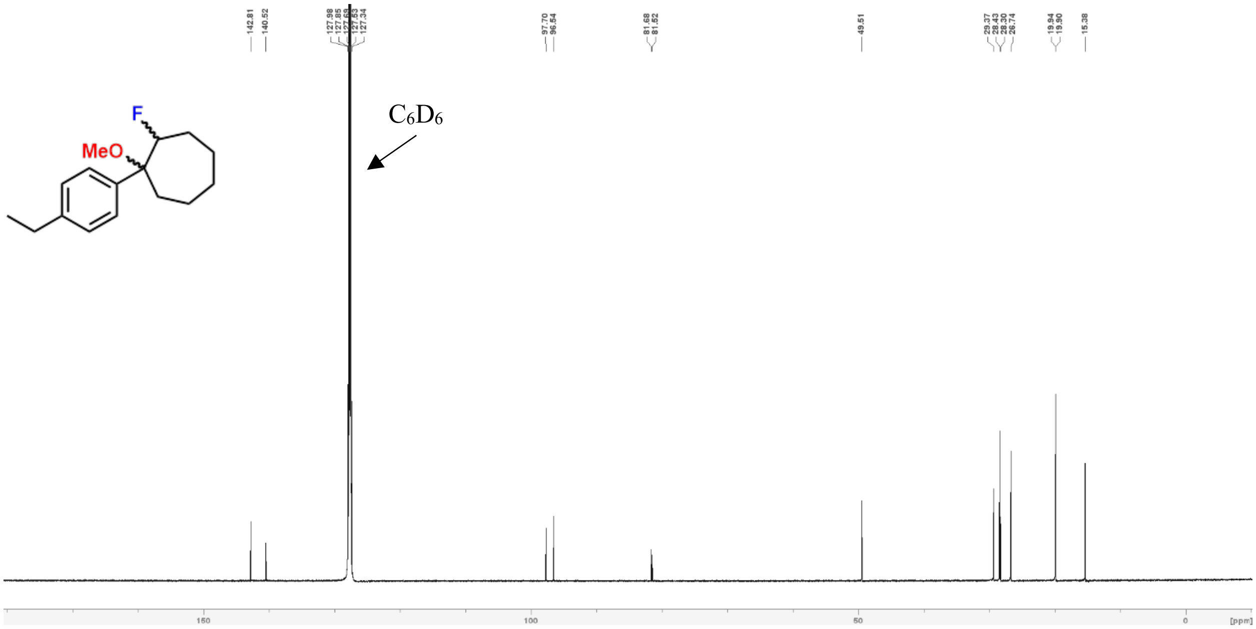

^{19}F NMR (565MHz, C_6D_6) of minor isomer 10ab

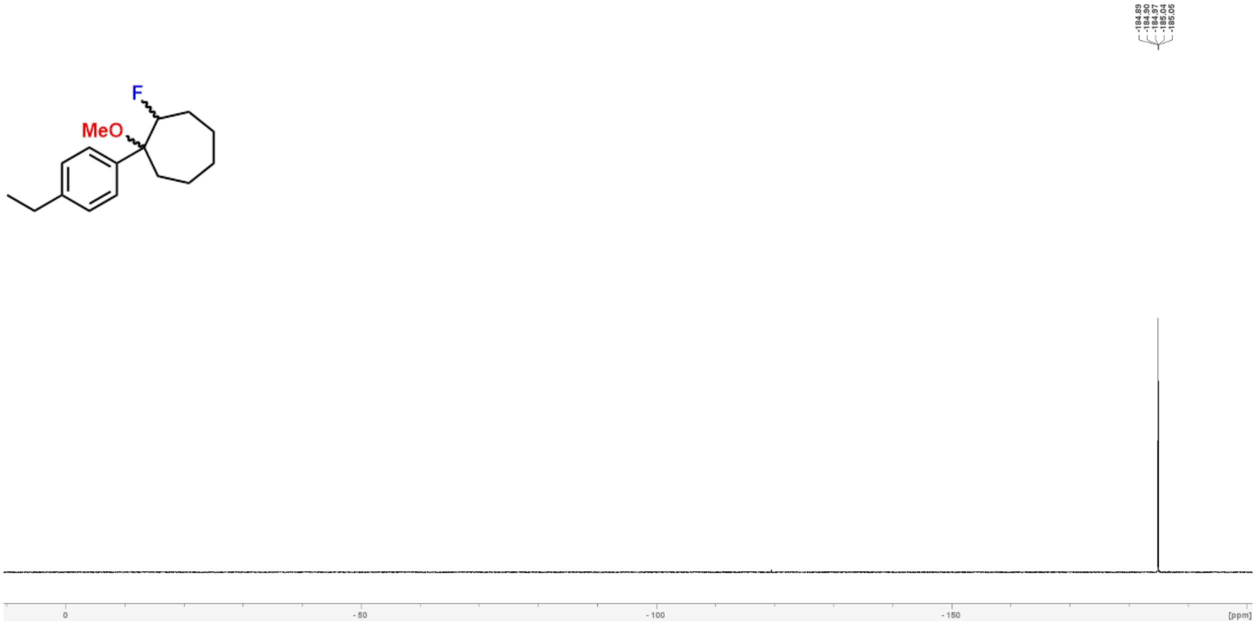

References

1. Sun, R., Xie, F., Zhang, Q., Sun, Y. J. & Dai, W. Ferric Nitrate as a Bifunctional Catalyst for Dehydration and Oxidative Cleavage-Esterification of Tertiary Alcohols. *Journal of Organic Chemistry* **90**, (2025).

2. Wang, J., Huang, B., Shi, C., Yang, C. & Xia, W. Visible-Light-Mediated Ring-Opening Strategy for the Regiospecific Allylation/Formylation of Cycloalkanols. *Journal of Organic Chemistry* **83**, 9696–9706 (2018).

3. Chowdhury, R., Dubey, A. K. & Ghosh, R. Synthesis of Functionalized Organosilicon Compounds/Distal Ketones via Ring-Opening Giese Addition of Cycloalkanols under Organophotocatalytic Conditions. *Journal of Organic Chemistry* **89**, 7187–7200 (2024).

4. Huang, M. *et al.* Selective, Transition Metal-free 1,2-Diboration of Alkyl Halides, Tosylates, and Alcohols. *Chemistry - A European Journal* **28**, e202200480 (2022).

5. Kwiatkowski, P., Beeson, T. D., Conrad, J. C. & MacMillan, D. W. C. Enantioselective organocatalytic α-fluorination of cyclic ketones. *J Am Chem Soc* **133**, 1738–1741 (2011).

6. Zupan, M., Iskra, J. & Stavber, S. The regioselectivity in the fluorination of dibenzofuran, diphenylether and biphenyl with N-F type of reagents. *Tetrahedron* **52**, 11341–11348 (1996).
